# Synthesis of Bis-heteroaryls Using Grignard Reagents and Pyridylsulfonium Salts

**DOI:** 10.1021/acs.orglett.1c03379

**Published:** 2021-11-16

**Authors:** Alexandra M. Horan, Vincent K. Duong, Eoghan M. McGarrigle

**Affiliations:** SSPC, the SFI Research Centre for Pharmaceuticals, Centre for Synthesis & Chemical Biology, UCD School of Chemistry, University College Dublin, Belfield, Dublin 4, Ireland

## Abstract

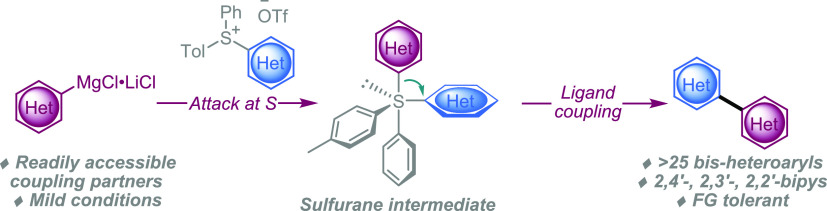

Herein are reported ligand-coupling reactions of Grignard reagents with pyridylsulfonium salts. The method has wide functional group tolerance and enables the formation of bis-heterocycle linkages, including 2,4′-, 2,3′-, and 2,2′-bipyridines, as well as pyridines linked to pyrimidines, pyrazines, isoxazoles, and benzothiophenes. The methodology was successfully applied to the synthesis of the natural products caerulomycin A and E.

Ligand-coupling reactions have recently experienced a resurgence in organic chemistry.^1−8^ They have been shown to be a powerful strategy for forming C(sp^2^)–C(sp^2^) bonds, most notably through phosphorane and sulfurane intermediates, obviating the need for costly transition metals.^1−9^ These hypervalent species can be used to synthesize a wide range of bis-aromatics, but perhaps most notably, access to bis-heterocycles such as bipyridines is enabled.^5−7^ Bis-heterocycles are privileged pharmacophores found in many natural products and therapeutics.^10−13^ Currently, the state-of-the-art for accessing bis-aromatics relies heavily on transition-metal-catalyzed cross-coupling methods. However, although aryl–aryl couplings with transition-metal-catalyzed cross couplings are remarkably efficient, the analogous heteroaryl–heteroaryl couplings are considerably more restricted.^14−16^ Willis and co-workers have addressed some of these issues through the use of pyridyl sulfinates; however, they have also noted that the incorporation of 2-pyridyl groups into compounds still requires much attention.^14−17^

Recently, we demonstrated that pyridylsulfonium salts react with lithiated pyridines to undergo ligand-coupling reactions to form bipyridines.^8^ This methodology is complementary to other recently disclosed ligand-coupling protocols to synthesize bipyridines by McNally^5,6^ and Qin (Scheme 1).^7^ Combined, these protocols offer a robust alternative to the costly transition-metal-catalyzed systems, with similar, if not wider functional group tolerance. Previously, we addressed some of the issues evident in other ligand-coupling protocols by accessing the 2,3′-bipyridine linkage and by demonstrating electron-donating group tolerance. Although our methodology was operationally simple with wide functional group tolerance, it too had limitations. Certain functional groups were not compatible with organolithium reagents, and we were unable to make 2,4′-bipyridine linkages (accessible through McNally and Qin’s methodologies). Organolithiums are inexpensive and highly reactive, which has proven attractive for C(sp^2^)–C(sp^2^) bond formation;^18^ however, their high reactivity is a disadvantage in terms of functional group tolerance.

**Scheme 1:**
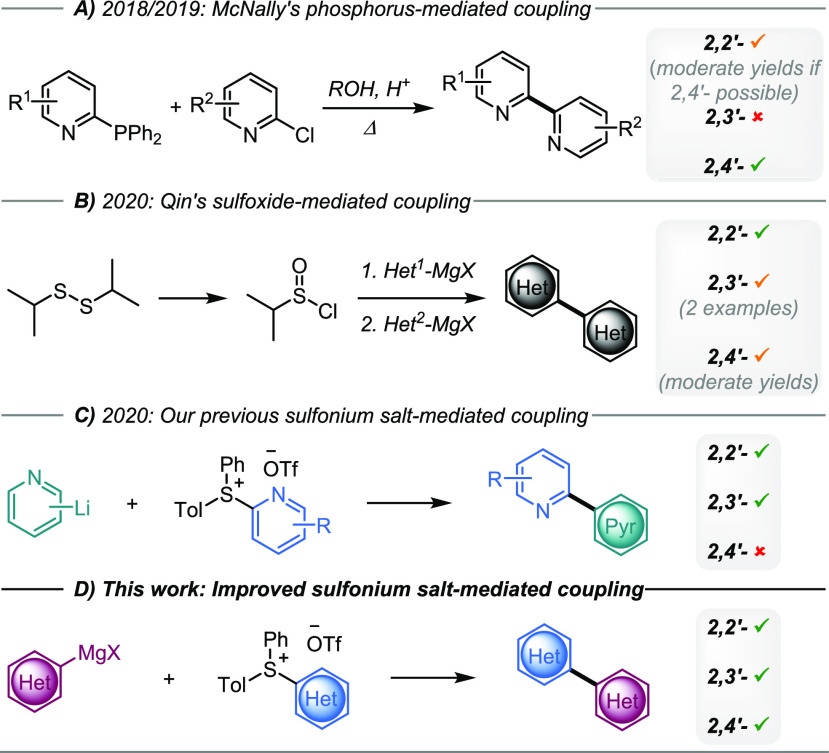
Bis-heteroaryl Syntheses via Ligand-Coupling Reactions to Access 2,2′-, 2,3′-, and 2,4′-Linkages

Herein, we report an improved sulfurane-meditated ligand-coupling protocol, augmenting the ligand-coupling approach further. We envisioned that pyridylsulfonium salts might undergo ligand-coupling reactions with alternative, milder organometallic reagents, enabling use of additional functional groups and possibly new linkages. We began by comparing the use of “turbo” Grignard reagents^19^ with the use of organolithiums in our previously reported methodology (Scheme 2). 2-Iodopyridine, 3-iodopyridine, and 4-iodopyridine were reacted with *i*-PrMgCl·LiCl to form the Grignard reagent *in situ*, followed by reaction with pyridylsulfonium salt **1a**. 2,2′-Bipyridine **2** and 2,3′-bipyridine **3** were synthesized in yields comparable to our previously developed organolithium method; however, most pleasingly, 2,4′-bipyridines could now be accessed with Grignard chemistry, completing the linkages accessible from 2-pyridylsulfonium salt **1a**. Extension to organozincs was also tested; however, initial results were very poor and further exploration was not undertaken. Thus, a single method using pyridylsulfonium salts **1** as a common precursor enables the synthesis of 2,2′-, 2,3′-, and 2,4′-bipyridines.

**Scheme 2:**
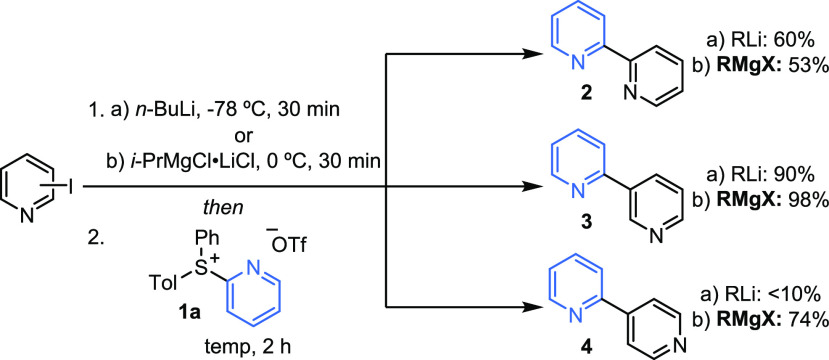
Ligand-Coupling Reactions between Grignard Reagents and Pyridylsulfonium Salt **1a** Reactions performed at 0.3 mmol scale, isolated yields indicated. Ligand-coupling reactions carried out at −78 °C for RLi and rt for RMgX.

We further explored the scope of the reaction, reacting a range of Grignard reagents with pyridylsulfonium salts (Table 1). In addition to known pyridylsulfonium salts **1a**–**g**,^8^ novel salts **1h**–**1l** were synthesized and applied in the ligand-coupling reaction. A new class of reagent, pyrimidinyldiarylsulfonium salt **1m**, was also tested in ligand couplings. Grignard reagents were generated from either halogenated pyridines or from C–H deprotonation (opening up the possibility for use as a late-stage functionalization strategy).^20^

**Table 1:**
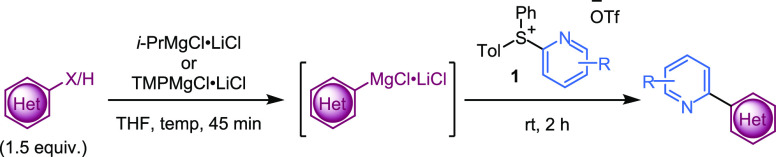

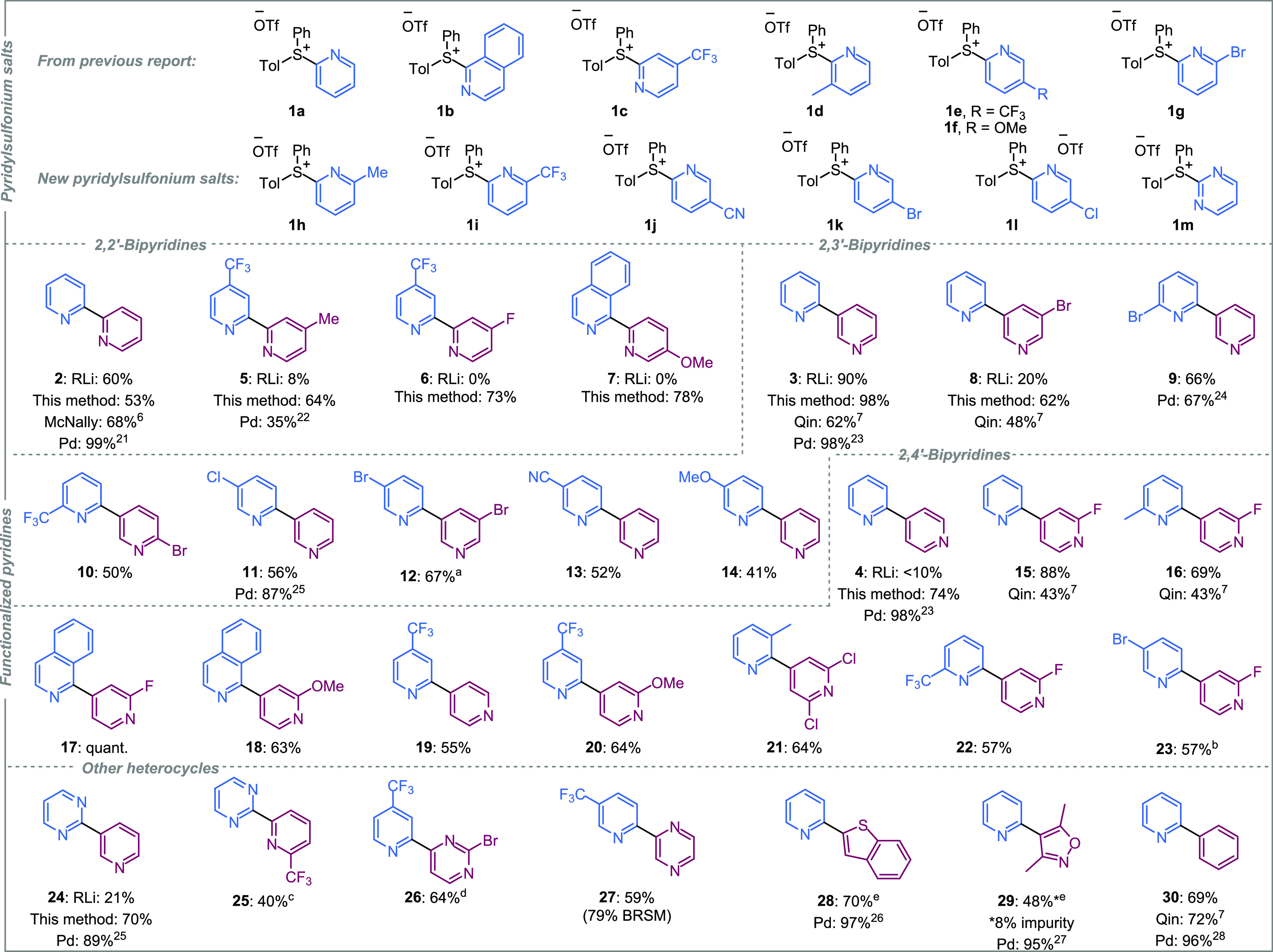
Synthesis of Bis-heterocycles Using Ligand-Coupling Methodology with Grignard Reagents and Pyridylsulfonium Salts **1**^f^

a Ligand-coupling reaction carried out at 45 °C for 3.5 h.

b Ligand-coupling reaction carried out at 45 °C for 4.5 h.

c Ligand-coupling reaction carried out at −78 °C.

d Ligand-coupling reaction carried out with *n*-BuMgCl at −78 °C.

e Ligand-coupling reaction carried out at 45 °C.

f Reactions were performed at the 0.3 mmol scale; isolated yields are indicated. Grignard reagents were formed at temperatures from 0 to 45 °C (see the Supporting Information for details). Grignard reagents were prepared from the corresponding halopyridine, except for compounds **21**, **26**, **27**, and **28** where directed C–H deprotonation was used.

A range of 2,2′-, 2,3′-, and, for the first time using a sulfonium intermediate, 2,4′-linked bipyridines were synthesized using our new protocol. A suite of 2,4′-linked bipyridines could now be accessed with functionalities such as halogens and ethers (**4** and **15**–**23**). These results represent a substantial improvement on sulfur-mediated couplings for this class of bipyridine. More generally, with the exception of parent 2,2′-bipyridine, **2**, the Grignard method gave improved yields of bipyridine versus our organolithium method or Qin’s sulfoxide method.^7,8^ Yields with palladium-catalyzed couplings were superior in 7 of the 10 cases available for comparison.^21−28^ In total, 16 novel bis-heteroaryls were synthesized, demonstrating the potential of ligand-coupling reactions as a complementary approach and enabling access to previously unexplored bis-heteroaryls. Thus, pyridylsulfonium salts represent a common building block for synthesis of a library of 2,2′-, 2,3′-, and 2,4′-pyridine-heteroaryl compounds.

For 2,2′- and 2,3′-bipyridines, we focused on testing Grignard reagents to address some of the limitations with organolithiums noted in our initial report. Functional groups such as boronic esters and phosphines that were incompatible with organolithiums remained challenging substrates for use with Grignard reagents; however, 4-fluorinated/methylated systems are now tolerated (**5** and **6**). Multihalogenated bipyridines (**6**, **10**, **12**, and **21**–**23**) were synthesized successfully, whereas they were low yielding substrates previously, possibly due to the excess organolithium reacting further with the desired products. In the case of **12** and **23**, longer reaction times were necessary for the reaction to go to completion at rt, but warming to 45 °C improved results. We also demonstrated that heterocycle–pyridine couplings could be achieved with our new methodology. Pyrimidine, pyrazine, and isoxazoles were competent coupling partners (**26**–**29**). Higher temperatures of 45 °C were necessary for the more electron-rich benzothiophene and isoxazole substrates to undergo ligand coupling (**28** and **29**). Pyrimidinylsulfonium salt **1m** gave an alternative route to pyridine-pyrimidine products (**24** and **25**). Ligand coupling at −78 °C gave bis-heteroaryl **25** in 40% yield, whereas poorer results were obtained at both rt (not isolated) and 45 °C (35%). The formation of 6,6′-bis(trifluoromethyl)-2,2′-bipyridine was noted, which is the first time we have observed evidence for ligand exchange^29^ with our sulfurane chemistry. At 45 °C, the pyridine–toluene product of a competing coupling reaction was observed also. These competing side reactions were not evident at −78 °C. Finally, 2-phenylpyridine was obtained in 69% yield, comparable to Qin’s method.^7^

With regard to mechanism, it is proposed that the reaction proceeds through a sulfurane intermediate, formed by the active Grignard species attacking the electropositive sulfur center of salt **1**. The first formed sulfurane intermediate may then undergo a series of pseudorotations leading to the active sulfurane intermediate (Scheme 3). A ligand-coupling sequence follows between one apical heterocyclic unit and one equatorial heterocycle to form the desired bis-heterocycle. However, direct S_N_Ar could also lead to the desired product. To test this hypothesis sulfonium salt **1a** was reacted with *i*-PrMgCl·LiCl. If the reaction proceeded through an S_N_Ar type process, the expected product would be the alkylated pyridine **33**, which was not observed with quantitative ^1^H NMR spectroscopic analysis. The expected ligand-coupling products **31** and **32** were obtained in 44% and 38% yield, respectively. Observation of both phenyl and tolyl coupling products is consistent with the reaction proceeding through a sulfurane intermediate such as **34**.

**Scheme 3:**
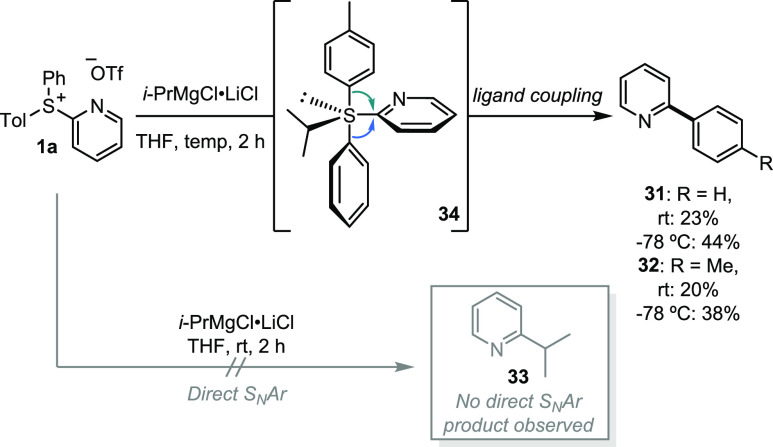
Testing the Possibility of S_N_Ar

Next, we applied our methodology to the synthesis of the natural products caerulomycins A and E (Scheme 4a). Key intermediate **36** was synthesized in 92% yield in a single step from salt **1a** and commercially available halopyridine **35**. This represented a significant improvement compared to our previous attempt using organolithium coupling partner (22% vs 92% yield). From key intermediate **36**, subsequent transformation to caerulomycin E **37** was achieved in 60% yield. Overall, this is a shorter and higher-yielding route (35% over 4 steps) compared to most previous syntheses of caerulomycin E,^30−33^ except for Duan’s synthesis (53% over 4 steps),^34^ proceeding from the arylation of nitropyridine *N*-oxides. Further transformation to caerulomycin A **38** was readily accomplished as previously demonstrated in the literature.^30,31,34^ The halogenated key intermediate **36** could serve as a precursor for the synthesis of caerulomycin analogues. We also successfully synthesized compound **17** on a 1.57 g scale with no deleterious effect on yield (Scheme 4b).

**Scheme 4:**
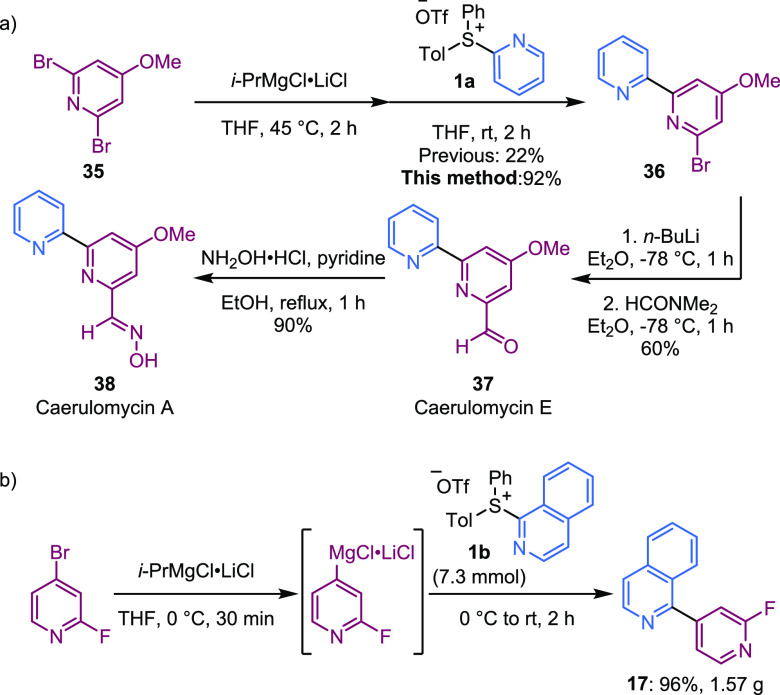
(a) Application of Ligand-Coupling Methodology to the Synthesis of Caerulomycins; (b) Gram-Scale Synthesis of Bis-Heterocycle **17**

In summary, through the use of mild Grignard reagents we have developed a common, modular route for the synthesis of 2,2′-, 2,3′-, and 2,4′-linked bipyridines. Further heteroaryl–pyridine couplings were also demonstrated with electron-rich and -poor heteroaryls. Our transition-metal-free bis-heteroaryl synthesis is a complementary methodology to existing phosphorus- and sulfur-mediated ligand-coupling procedures. Together these protocols offer attractive alternatives to the venerable transition-metal-catalyzed cross-coupling reactions. Indeed, we believe that the further development of these protocols will lead to their establishment as strategic reaction alternatives in the synthetic organic chemist’s toolkit.

## Supporting Information Available

Experimental procedures, characterization, and copies of NMR spectra (PDF)
