# Supplementary material for: Synthesis of Bis-heteroaryls Using Grignard Reagents and Pyridylsulfonium Salts
Source: Org Lett. 2021 Nov 16;23(23):9089–93. doi: 10.1021/acs.orglett.1c03379 (PMC8650099; doi:10.1021/acs.orglett.1c03379)
Supplement: Supplementary file 1 — ol1c03379_si_001.pdf [file ol1c03379_si_001.pdf]

Synthesis of Bis-heteroaryls using Grignard Reagents and Pyridylsulfonium Salts

Alexandra M. Horan, Vincent K. Duong,* Eoghan M. McGarrigle*

SSPC, the SFI Research Centre for Pharmaceuticals, Centre for Synthesis & Chemical Biology, UCD School of Chemistry, University College Dublin, Belfield, Dublin 4, Ireland.

Supporting Information

Contents

1. General experimental **2**

2. Synthesis of sulfides **2**

3. Synthesis of sulfonium salts **7**

4. Synthesis of bipyridines **11**

5. Synthesis of Caerulomycin E and A **27**

6. NMR Spectra **30**

7. References **74**

1. General experimental

Chemicals were purchased and used without further purification unless otherwise stated. Solvents were dried using a Grubbs-type still, a Pure Solv-400-3-MD solvent purification system supplied by Innovative Technology Inc. design and stored in Strauss flasks over activated 4Å molecular sieves. Diphenyliodonium trifluoromethanesulfonate was prepared according to literature procedure.[1] Pyridylsulfides **1ai-1gi** and sulfonium salts **1a-1g** were prepared as previously described.[2] TMPMgCl•LiCl was prepared according to literature procedure.[3] *i*-PrMgCl•LiCl and TMPMgCl•LiCl were titrated prior to use.[4]

Reactions requiring anhydrous conditions were performed under N_2; glassware was flame-dried immediately prior to use and allowed to cool under reduced pressure. Reaction monitoring by TLC was performed on Merck pre-coated Kieselgel 60 F_{254} aluminium plates. Visualization was accomplished under UV light (254 nm). Flash column chromatography (FCC) was performed using either silica gel [Davisil, 230-400 mesh (40-63 µm)] or using a Biotage Isolera™ UV-VIS Flash Purification System Version 2.3.1 with SNAP Ultra (25 µm), SNAP KP-Sil (50 µm) or SNAP KP-NH (50 µm) prepacked silica cartridges. High-resolution mass spectra were run on a Waters Micromass GCT system or on an Agilent 6546 QTOF system in electrospray ionization mode (ESI). Extracts were concentrated *in vacuo* using both a rotary evaporator (bath temperatures up to 55 °C), and a high vacuum line at room temperature. ^1H NMR and ^{13}C NMR spectra were measured in the solvent stated at 300 MHz, 400 MHz or 500 MHz or 100 – 126 MHz, respectively. ^{19}F spectra was measured in the solvent stated at 376 – 470 MHz. Chemical shifts (δ) are quoted in parts per million (ppm) referenced to residual solvent peak (e.g., $CDCl_3$: ^1H – 7.26 ppm and ^{13}C – 77.16 ppm) or TMS (^1H – 0.00 ppm) and coupling constants (*J*) are given in Hertz. Multiplicities are abbreviated as: b (broad), s (singlet), d (doublet), t (triplet), q (quartet), m (multiplet) or combinations thereof. Assignments were made, where necessary, with the aid of COSY, HSQC, HMBC and NOESY NMR experiments.

2. Synthesis of sulfides

General procedure A: 4-methylbenzenethiol (1 equiv.) was added to a round bottomed flask, followed by halopyridine (1 equiv.) and H_2O (0.5 M). The reaction was heated under reflux for 24 h (the flask sitting in an aluminium block placed on a stirrer hotplate). Then, the reaction was allowed to cool, followed by extraction with EtOAc. The combined organic layers were washed with water and brine and dried over Na_2SO_4. The solvent was evaporated, and the product was isolated by FCC.

General procedure B: 4-methylbenzenethiol (1 equiv.) was added to a crimptop vial followed by halopyridine (1 equiv.) and K_2CO_3 (1.1 equiv.) and closed. The vial was evacuated and purged with N_2 three times. Anhydrous DMF (0.8–1 M wrt thiol/halopyridine) was added. The reaction was heated to 100 °C for 24 h (the flask sitting in an aluminium block placed on a stirrer hotplate). Then the reaction

was allowed to cool, followed by the addition of water and extraction with EtOAc. The combined organic layers were washed with water and brine and dried over Na_2SO_4. Details of purification are given below for each compound.

2-Methyl-6-(*p*-tolylthio)pyridine 1hi

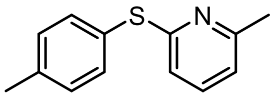

Prepared from General procedure B with 4-methylbenzenethiol (0.62 g, 5.0 mmol), 2-bromo-6-methylpyridine (0.57 mL, 5.0 mmol), K_2CO_3 (0.88 g, 6.0 mmol) and DMF (6 mL). The reaction was extracted with EtOAc (3 x 15 mL). The combined organic layers were washed with water (20 mL) and brine (20 mL) and dried over Na_2SO_4. The solvent was evaporated to give pyridylsulfide **1hi** as a clear oil (1.05 g, 98%).

TLC: R_f = 0.3(10% Et_2O in pentane).

^{1}H NMR (500 MHz, Chloroform-*d*) δ 7.50 – 7.46 (m, 2H, ArH), 7.28 (t, *J* = 7.8 Hz, 1H, ArH), 7.24 – 7.18 (m, 2H, ArH), 6.81 (d, *J* = 7.6 Hz, 1H, ArH), 6.55 (d, *J* = 8.0 Hz, 1H, ArH), 2.49 (s, 3H, CH_3), 2.38 (s, 3H, CH_3) ppm.

^{13}C NMR (126 MHz, Chloroform-*d*) δ 161.9 (C), 158.5 (C), 139.5 (C), 137.0 (CH), 135.4 (CH), 130.5 (CH), 127.6 (C), 119.2 (CH), 117.7 (CH), 24.4 (CH_3), 21.4 (CH_3) ppm.

HRMS (ESI-TOF) *m/z*: $[M+H]^+$ Calcd for $C_{13}H_{14}NS$: 216.0841; Found: 216.0844.

2-(*p*-Tolylthio)-6-(trifluoromethyl)pyridine 1ii

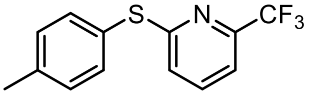

Prepared from General procedure B with slight modifications, with 4-methylbenzenethiol (0.56 g, 4.5 mmol), 2-bromo-6-(trifluoromethyl)pyridine (0.68 g, 3.0 mmol), K_2CO_3 (0.50 g, 3.6 mmol) and DMF (1.9 mL). The reaction was heated to 140 °C for 7 h. The reaction was extracted with EtOAc (3 x 20 mL). The combined organic layers were washed with water (3 x 30 mL) and brine (3 x 30 mL) and dried over Na_2SO_4. The solvent was evaporated and purification by FCC (5% Et_2O in pentane) gave pyridylsulfide **1ii** as a white solid (0.52 g, 64%).

TLC: R_f = 0.50 (5% Et_2O in pentane).

^{1}H NMR (500 MHz, Chloroform-*d*) δ 7.57 (t, *J* = 7.9 Hz, 1H), 7.52 (d, *J* = 8.2 Hz, 2H), 7.33 (d, *J* = 7.7 Hz, 1H), 7.28 (d, *J* = 7.8 Hz, 2H), 6.93 (d, *J* = 8.1 Hz, 1H), 2.43 (s, 3H, CH_3).

^{13}C NMR (101 MHz, Chloroform-*d*) δ 164.3 (C), 147.9 (q, J = 34.8 Hz, *C*CF_3), 140.3 (C), 137.7 (CH), 135.7 (CH), 130.9 (CH), 126.1 (C), 123.2 (CH), 121.3 (d, J = 274.3 Hz, CF_3), 116.1 (q, J = 2.7 Hz, CH), 21.5 (CH_3).

^{19}F NMR (282 MHz, Chloroform-*d*) δ -68.2.

HRMS (ESI-TOF) *m/z*: $[M+H]^+$ Calcd for $C_{13}H_{11}F_3NS$: 270.0559; Found: 270.0561.

6-(*p*-Tolylthio)nicotinonitrile 1ji

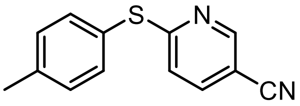

Prepared from General procedure B with slight modifications, with 4-methylbenzenethiol (1.65 g, 13.3 mmol), 2-bromo-5-cyanopyridine (2.03 g, 11.1 mmol), K_2CO_3 (2.31 g, 16.7 mmol) and DMF (5.6 mL). The reaction was heated to 140 °C for 16 h. The reaction was extracted with EtOAc (3 x 20 mL). The combined organic layers were washed with water (3 x 30 mL) and brine (3 x 30 mL) and dried over Na_2SO_4. The solvent was evaporated and purification by FCC (20% Et_2O in pentane) gave pyridylsulfide **1ji** as a white solid (1.60 g, 64%).

TLC: R_f = 0.37 (20% Et_2O in pentane).

^{1}H NMR (400 MHz, Chloroform-*d*) δ 8.62 (d, J = 2.1 Hz, 1H), 7.61 (dd, J = 8.5, 2.3 Hz, 1H), 7.48 (d, J = 8.0 Hz, 2H), 7.29 (d, J = 7.8 Hz, 2H), 6.87 (d, J = 8.4 Hz, 1H), 2.42 (s, 3H, CH_3).

^{13}C NMR (101 MHz, Chloroform-*d*) δ 168.7, 152.2, 140.9, 138.9, 135.7, 131.0, 125.0, 120.0, 117.0, 105.0, 21.5 (CH_3).

Spectra were consistent with literature data.[5]

5-Bromo-2-(*p*-tolylthio)pyridine 1ki

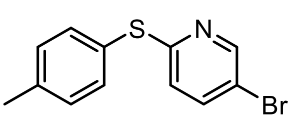

Prepared from General procedure B with slight modifications, with 4-methylbenzenethiol (0.37 g, 3.0 mmol), 2,5-dibromopyridine (0.71 g, 3.0 mmol), K_2CO_3 (0.5 g, 3.6 mmol) and DMF (1.9 mL). The reaction was heated to 140 °C for 7 h. The reaction was extracted with EtOAc (3 x 20 mL). The combined organic layers were washed with water (3 x 30 mL) and brine (3 x 30 mL) and dried over Na_2SO_4. The solvent was evaporated and purification by FCC (5% Et_2O in pentane) gave pyridylsulfide **1ki** as a white solid (0.36 g, 43%).

TLC: R_f = 0.36 (5% Et_2O in pentane).

^{1}H NMR (400 MHz, Chloroform-*d*) δ 8.45 (d, *J* = 2.4 Hz, 1H), 7.52 (dd, *J* = 8.6, 2.4 Hz, 1H), 7.47 (d, *J* = 7.9 Hz, 2H), 7.24 (d, *J* = 7.8 Hz, 2H), 6.73 (dd, *J* = 8.6, 0.6 Hz, 1H), 2.40 (s, 3H, CH_3).

^{13}C NMR (101 MHz, Chloroform-*d*) δ 161.1 (C), 150.4 (CH), 140.0 (C), 139.2 (CH), 135.4 (CH), 130.7 (CH), 126.9 (C), 122.3 (CH), 116.3 (C), 21.5 (CH_3).

HRMS (ESI-TOF) *m/z*: $[M+H]^+$ Calcd for $C_{12}H_{11}BrNS$: 279.9790, 281.9770; Found: 279.9792, 281.9771.

5-Chloro-2-(*p*-tolylthio)pyridine 1li

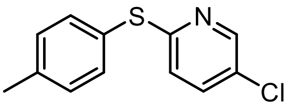

Prepared from General procedure B with slight modifications, with 4-methylbenzenethiol (0.5 g, 400 mmol), 2-bromo-5-chloropyridine (0.77 g, 4.0 mmol), K_2CO_3 (0.66 g, 4.8 mmol) and DMF (2.5 mL). The reaction was heated to 140 °C for 7 h. The reaction was extracted with EtOAc (3 x 20 mL). The combined organic layers were washed with water (3 x 30 mL) and brine (3 x 30 mL) and dried over Na_2SO_4. The solvent was evaporated and purification by FCC (5% Et_2O in pentane) gave pyridylsulfide **1li** as a white solid (0.75 g, 79%).

TLC: R_f = 0.23 (5% Et_2O in pentane).

^{1}H NMR (400 MHz, Chloroform-*d*) δ 8.37 – 8.36 (m, 1H), 7.47 (d, *J* = 8.1 Hz, 2H), 7.39 (dd, *J* = 8.6, 2.7 Hz, 1H), 7.24 (d, *J* = 8.2 Hz, 2H), 6.79 (dd, *J* = 8.6, 0.6 Hz, 1H), 2.40 (s, 3H, CH_3).

^{13}C NMR (101 MHz, Chloroform-*d*) δ 160.5 (C), 148.3 (CH), 139.9 (C), 136.5 (CH), 135.3 (CH), 130.7 (CH), 128.1 (C), 127.0 (C), 121.8 (CH), 21.5 (CH_3).

HRMS (ESI-TOF) *m/z*: $[M+H]^+$ Calcd for $C_{12}H_{11}ClNS$: 236.0295, 238.0266; Found: 236.0297, 238.0265.

2-(*p*-Tolylthio)pyrimidine 1mi

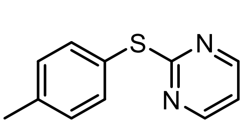

Prepared from General procedure B with slight modifications, with 4-methylbenzenethiol (1.91 g, 15.4 mmol), 2-bromopyrimidine (2.04 g, 12.8 mmol), K_2CO_3 (2.65 g, 19.2 mmol) and DMF (6.4 mL). The reaction was heated to 140 °C for 7 h. The reaction was extracted with EtOAc (3 x 20 mL). The combined organic layers were washed with water (3 x 30 mL) and brine (3 x 30 mL) and dried over Na_2SO_4. The solvent was evaporated and purification by FCC (20% Et_2O in pentane) gave pyridylsulfide **1mi** as a white solid (1.81 g, 70%).

TLC: R_f = 0.25 (20% Et_2O in pentane).

^{1}H NMR (400 MHz, Chloroform-*d*) δ 8.46 (d, *J* = 5.0 Hz, 2H), 7.50 (d, *J* = 8.1 Hz, 2H), 7.23 (d, *J* = 7.7 Hz, 2H), 6.93 (t, *J* = 4.9 Hz, 1H), 2.38 (s, 3H, CH_3).

^{13}C NMR (101 MHz, Chloroform-*d*) δ 173.4 (C), 157.7 (CH), 139.7 (C), 135.4 (CH), 130.3 (CH), 125.9 (C), 117.0 (CH), 21.5 (CH_3).

HRMS (ESI-TOF) *m/z*: $[M+H]^+$ Calcd for $C_{11}H_{11}N_2S$: 203.0637; Found: 203.0638.

3. Synthesis of sulfonium salts

General procedure C: Sulfide (1.1 equiv.), Ph_2IOTf (1.0 equiv.) and $Cu(OTf)_2$ (5 mol%) were added to a crimptop vial and sealed. The vial was evacuated and purged with N_2 three times. Dry DCE (0.6 M) was added to the vial and the reaction was heated to 95 °C for 16 h (the vial sitting in an aluminium block placed on a stirrer hotplate). Then the reaction was allowed to cool and sat. aq. NH_4Cl (20 mL) was added. The product was extracted using CH_2Cl_2 (3 x 20 mL) and washed with H_2O (2 x 20 mL) and brine (20 mL). The solvent was evaporated and product was isolated by FCC.

(6-Methylpyridin-2-yl)(phenyl)(*p*-tolyl)sulfonium trifluoromethanesulfonate 1h

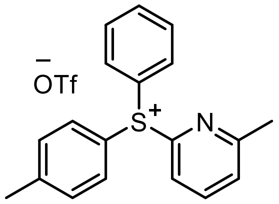

Product **1h** was synthesised *via* General procedure C with sulfide **1hi** (1.0 g, 4.6 mmol), Ph_2IOTf (1.81 g, 4.2 mmol) and $Cu(OTf)_2$ (76 mg, 0.21 mmol). Purification by FCC (5% MeOH in CH_2Cl_2) gave sulfonium salt **1h** as a yellow oil (575 mg, 31%).

^1H NMR (500 MHz, Chloroform-*d*) δ 8.13 – 8.09 (m, 1H), 7.97 (t, *J* = 7.8 Hz, 1H), 7.91 – 7.85 (m, 2H), 7.84 – 7.79 (m, 2H), 7.77 – 7.69 (m, 1H), 7.69 – 7.63 (m, 2H), 7.52 – 7.44 (m, 3H), 2.60 (s, 3H), 2.46 (s, 3H).

^{13}C NMR (126 MHz, Chloroform-*d*) δ 162.8 (C), 146.4 (C), 146.1 (CH), 140.7 (CH), 134.5 (CH), 132.1 (CH), 132.0 (CH), 131.6 (CH), 131.3 (CH), 128.5 (C), 126.9 (CH), 125.4 (C), 121.0 (C), 24.5 (CH_3), 21.9 (CH_3). Peak for SO_3CF_3 not observed.

HRMS (ESI$^+$): [M-OTf]$^+$ Calcd for $C_{19}H_{18}NS^+$: 292.1154; found: 292.1156.

Phenyl(*p*-tolyl)(6-(trifluoromethyl)pyridin-2-yl)sulfonium trifluoromethanesulfonate 1i

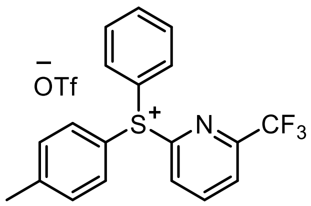

Product **1i** was synthesised *via* General procedure C with sulfide **1ii** (0.65 g, 2.42 mmol), Ph_2IOTf (1.04 g, 2.42 mmol) and $Cu(OTf)_2$ (43 mg, 0.12 mmol). Purification by FCC (10% MeOH in CH_2Cl_2) gave sulfonium salt **1i** as a brown oil (1.03 g, 86%).

TLC: R_f = 0.30 (10% MeOH in CH_2Cl_2).

^{1}H NMR (400 MHz, Chloroform-*d*) δ 8.50 (d, *J* = 8.1 Hz, 1H), 8.37 (t, *J* = 8.0 Hz, 1H), 8.02 (d, *J* = 7.8 Hz, 1H), 7.86 (dd, *J* = 7.7, 1.7 Hz, 2H), 7.81 (d, *J* = 8.4 Hz, 2H), 7.78 – 7.69 (m, 1H), 7.69 – 7.61 (m, 2H), 7.47 (d, *J* = 8.2 Hz, 2H), 2.43 (s, 3H, CH_3).

^{13}C NMR (101 MHz, Chloroform-*d*) δ 150.2 (q, *J* = 37.0 Hz, *C*CF_3), 147.6 (C), 147.1 (C), 143.3 (CH), 135.0 (CH), 132.3 (CH), 132.0 (2 x CH), 131.6 (CH), 131.5 (CH), 125.2 (q, *J* = 2.4 Hz, CH), 124.4 (C), 120.8 (q, *J* = 320.8 Hz, OTf), 120.2 (q, *J* = 274.9 Hz, CF_3), 119.9 (C), 21.7 (CH_3).

^{19}F NMR (376 MHz, Chloroform-*d*) δ -68.1, -78.3.

HRMS (ESI-TOF) *m/z*: $[M\text{-}OTf]^+$ Calcd for $C_{19}H_{15}F_3NS$: 346.0872; Found: 346.0874.

(5-Cyanopyridin-2-yl)(phenyl)(*p*-tolyl)sulfonium trifluoromethanesulfonate 1j

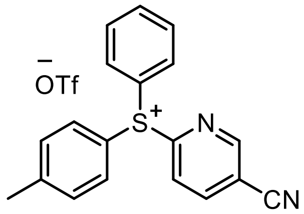

Product **1j** was synthesised *via* General procedure C with sulfide **1ji** (1.52 g, 6.73 mmol), Ph_2IOTf (2.90 g, 6.73 mmol) and $Cu(OTf)_2$ (122 mg, 0.34 mmol). Purification by FCC (10% MeOH in CH_2Cl_2) gave sulfonium salt **1j** as a brown oil (1.63 g, 53%).

TLC: R_f = 0.36 (10% MeOH in CH_2Cl_2).

^{1}H NMR (500 MHz, Chloroform-*d*) δ 8.92 (d, *J* = 2.1 Hz, 1H), 8.51 (d, *J* = 8.2 Hz, 1H), 8.35 (dd, *J* = 8.3, 2.3 Hz, 1H), 7.89 (d, *J* = 8.0 Hz, 2H), 7.83 (d, *J* = 8.3 Hz, 2H), 7.76 (t, *J* = 7.5 Hz, 1H), 7.68 (t, *J* = 7.8 Hz, 2H), 7.50 (d, *J* = 8.2 Hz, 2H), 2.47 (s, 3H, CH_3).

^{13}C NMR (126 MHz, Chloroform-*d*) δ 153.8 (CH), 151.0 (C), 147.3 (C), 144.1 (CH), 135.1 (CH), 132.5 (CH), 132.3 (CH), 132.0 (CH), 131.7 (CH), 129.5 (CH), 124.0 (C), 120.8 (q, *J* = 320.3 Hz, OTf), 119.6 (C), 114.7 (CN), 114.5 (*C*CN), 21.9 (CH_3).

^{19}F NMR (470 MHz, Chloroform-*d*) δ -78.3.

HRMS (ESI-TOF) *m/z*: $[M\text{-}OTf]^+$ Calcd for $C_{19}H_{15}N_2S$: 303.0950; Found: 303.0954.

(5-Bromopyridin-2-yl)(phenyl)(*p*-tolyl)sulfonium trifluoromethanesulfonate 1k

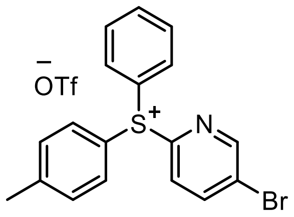

Product **1k** was synthesised *via* General procedure C with sulfide **1ki** (2.48 g, 8.85 mmol), Ph_2IOTf (3.17 g, 7.38 mmol) and $Cu(OTf)_2$ (133 mg, 0.37 mmol). Purification by FCC (10% MeOH in CH_2Cl_2) gave sulfonium salt **1k** as a brown oil (2.87 g, 77%).

TLC: R_f = 0.36 (10% MeOH in CH_2Cl_2).

^{1}H NMR (500 MHz, Chloroform-*d*) δ 8.77 (d, *J* = 2.1 Hz, 1H), 8.55 – 8.47 (m, 1H), 8.25 – 8.18 (m, 1H), 7.90 (d, *J* = 7.9 Hz, 2H), 7.85 (d, *J* = 8.3 Hz, 2H), 7.73 (t, *J* = 7.4 Hz, 1H), 7.65 (t, *J* = 7.5 Hz, 2H), 7.47 (d, *J* = 7.5 Hz, 2H), 2.45 (s, 3H, CH_3).

^{13}C NMR (126 MHz, Chloroform-*d*) δ 153.4 (CH), 146.7 (C), 145.5 (C), 143.2 (CH), 134.7 (CH), 132.19 (CH), 132.17 (CH), 131.8 (CH), 131.4 (CH), 131.2 (CH), 126.9 (C), 124.9 (C), 120.9 (q, *J* = 320.5 Hz, OTf), 120.6 (C), 21.8 (CH_3).

^{19}F NMR (470 MHz, Chloroform-*d*) δ -78.2.

HRMS (ESI-TOF) *m/z*: $[M-OTf]^+$ Calcd for $C_{18}H_{15}BrNS$: 356.0103, 358.0083; Found: 356.0104, 358.0083.

(5-Chloropyridin-2-yl)(phenyl)(*p*-tolyl)sulfonium trifluoromethanesulfonate 1l

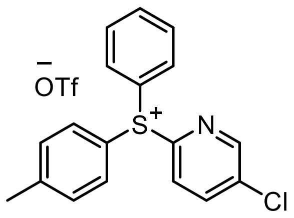

Product **1l** was synthesised *via* General procedure C with sulfide **1li** (0.32 g, 1.35 mmol), Ph_2IOTf (0.58 g, 1.35 mmol) and $Cu(OTf)_2$ (25 mg, 0.07 mmol). Purification by FCC (10% MeOH in CH_2Cl_2) gave sulfonium salt **1l** as a brown oil (0.410 g, 66%).

TLC: R_f = 0.30 (10% MeOH in CH_2Cl_2).

^{1}H NMR (500 MHz, Chloroform-*d*) δ 8.67 (d, *J* = 2.2 Hz, 1H), 8.37 (d, *J* = 8.4 Hz, 1H), 8.05 (dd, *J* = 8.3, 2.5 Hz, 1H), 7.88 – 7.82 (m, 2H), 7.80 (d, *J* = 8.4 Hz, 2H), 7.76 – 7.70 (m, 1H), 7.69 – 7.62 (m, 2H), 7.46 (d, *J* = 8.3 Hz, 2H), 2.44 (s, 3H, CH_3).

^{13}C NMR (101 MHz, Chloroform-*d*) δ 151.3 (CH), 146.8 (C), 144.7 (C), 140.2 (CH), 137.9 (C), 134.7 (CH), 132.2 (CH), 131.9 (CH), 131.6 (CH), 131.4 (CH), 130.6 (CH), 124.8 (C), 120.9 (q, *J* = 320.6 Hz, OTf), 120.4 (C), 21.8 (CH_3).

^{19}F NMR (376 MHz, Chloroform-*d*) δ -78.2.

HRMS (ESI-TOF) *m/z*: $[M\text{-}OTf]^+$ Calcd for $C_{18}H_{15}ClNS$: 312.0608, 314.0579; Found: 312.0610, 314.0578.

Phenyl(pyrimidin-2-yl)(*p*-tolyl)sulfonium trifluoromethanesulfonate 1m

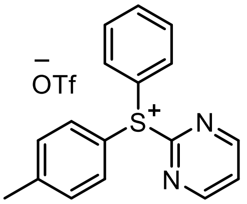

Product **1m** was synthesised *via* General procedure C with sulfide **1mi** (1.71 g, 8.45 mmol), Ph_2IOTf (3.64 g, 8.45 mmol) and $Cu(OTf)_2$ (153 mg, 0.42 mmol). Purification by FCC (10% MeOH in CH_2Cl_2) gave sulfonium salt **1m** as a brown solid (2.50 g, 69%).

TLC: R_f = 0.32 (10% MeOH in CH_2Cl_2).

^1H NMR (500 MHz, Chloroform-*d*) δ 8.89 (d, *J* = 4.8 Hz, 2H), 7.87 (d, *J* = 7.6 Hz, 2H), 7.78 (d, *J* = 8.5 Hz, 2H), 7.77 – 7.75 (m, 1H), 7.72 (t, *J* = 7.4 Hz, 1H), 7.68 – 7.63 (m, 2H), 7.47 (d, *J* = 8.3 Hz, 2H), 2.42 (s, 3H, CH_3).

^{13}C NMR (126 MHz, Chloroform-*d*) δ 161.0 (CH), 160.1 (C), 147.0 (C), 135.0 (CH), 132.5 (CH), 132.3 (CH), 132.2 (CH), 131.5 (CH), 124.9 (CH), 124.4 (C), 120.8 (q, *J* = 320.8 Hz, OTf), 120.0 (C), 21.8 (CH_3).

^{19}F NMR (376 MHz, Chloroform-*d*) δ -78.2.

HRMS (ESI-TOF) *m/z*: $[M\text{-}OTf]^+$ Calcd for $C_{17}H_{15}N_2S$: 279.0950; Found: 279.0953.

4. Synthesis of bis-heteroaryls

General procedure D: Halopyridine (0.45 mmol, 1.5 equiv.) was added to an oven-dried crimptop vial and sealed. The vial was evacuated and purged with N_2 three times. The halopyridine was dissolved in dry THF (1.5 mL) and heated/cooled to a given temperature. *i*-PrMgCl•LiCl (0.38 mL, 0.45 mmol, 1.5 equiv., 1.2 M in THF) was added dropwise to the stirring solution over 2 min. The reaction was allowed to stir for a given time at the specified temperature. Sulfonium salt (0.3 mmol, 1.0 equiv.) was added to a separate oven-dried crimptop vial and dissolved in dry THF (1.5 mL). The solution of sulfonium salt was added dropwise down the side of the vial to the Grignard reagent solution over 2 min. The reaction was allowed to stir for 2 h. Sat. aq. NH_4Cl (3 mL) was added slowly to quench any excess Grignard reagent. The product was extracted with EtOAc (3 x 10 mL), the combined organic layers were washed with H_2O (20 mL) and brine (20 mL). The solvent was evaporated, and the product was isolated by FCC.

General procedure E: Pyridine (0.45 mmol, 1.5 equiv.) was added to an oven-dried crimptop vial and sealed. The vial was evacuated and purged with N_2 three times. The pyridine was dissolved in dry THF (1.5 mL) and heated/cooled to a given temperature. TMPMgCl•LiCl (0.41 mL, 0.45 mmol, 1.5 equiv., 1.1 M in THF) was added dropwise to the stirring solution over 2 min. The reaction was allowed to stir for a given time at the specified temperature. Sulfonium salt (0.3 mmol, 1.0 equiv.) was added to a separate oven-dried crimptop vial and dissolved in dry THF (1.5 mL). The solution of sulfonium salt was added dropwise down the side of the vial to the Grignard reagent solution over 2 min. The reaction was allowed to stir for 2 h. Sat. aq. NH_4Cl (3 mL) was added slowly to quench any excess Grignard reagent. The product was extracted with EtOAc (3 x 10 mL), the combined organic layers were washed with H_2O (20 mL) and brine (20 mL). The solvent was evaporated, and the product was isolated by FCC.

2,2'-Bipyridine 2

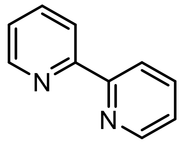

Product **2** was synthesised *via* General procedure D using 2-iodopyridine (32 µL, 0.45 mmol) and sulfonium salt **1a** (128.2 mg, 0.3 mmol). The Grignard reagent was formed at 0 °C for 30 min and the ligand coupling reaction was stirred at 0 °C. Purification by FCC (80% Et_2O in pentane) gave bipyridine **2** as a white solid (24.8 mg, 53%).

^{1}H NMR (400 MHz, Chloroform-*d*) δ 8.74 – 8.61 (m, 2H), 8.40 (d, *J* = 8.0 Hz, 2H), 7.82 (td, *J* = 7.7, 1.8 Hz, 2H), 7.31 (ddd, *J* = 7.6, 4.8, 1.2 Hz, 2H) ppm.

^{13}C NMR (101 MHz, Chloroform-*d*) δ 156.3, 149.4, 137.1, 123.9, 121.2 ppm.

Spectra are consistent with literature data.[6]

2,3'-Bipyridine 3

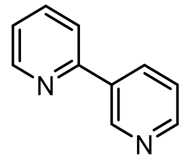

Product **3** was synthesised *via* General procedure D using 3-iodopyridine (93 mg, 0.45 mmol) and sulfonium salt **1a** (128.2 mg, 0.3 mmol). The Grignard reagent was formed at rt for 30 min and the ligand coupling reaction was stirred at rt. Purification by FCC (80% Et_2O in pentane) gave bipyridine **3** as a yellow oil (46.0 mg, 98%).

^{1}H NMR (400 MHz, Chloroform-*d*) δ 9.20 (d, *J* = 2.3 Hz, 1H), 8.76 – 8.70 (m, 1H), 8.66 (dd, *J* = 4.9, 1.7 Hz, 1H), 8.33 (dt, *J* = 8.1, 2.0 Hz, 1H), 7.85 – 7.72 (m, 2H), 7.41 (dd, *J* = 7.9, 4.9 Hz, 1H), 7.33 – 7.26 (m, 1H) ppm.

^{13}C NMR (101 MHz, Chloroform-*d*) δ 154.9, 150.2, 150.0, 148.3, 137.1, 135.0, 134.5, 123.7, 123.0, 120.7 ppm.

Spectra are consistent with literature data.[7]

2,4'-Bipyridine 4

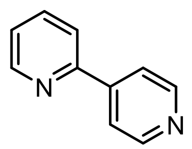

Product **4** was synthesised *via* General procedure D using 4-iodopyridine (93 mg, 0.45 mmol) and sulfonium salt **1a** (128.2 mg, 0.3 mmol). The Grignard reagent was formed at rt for 30 min and the ligand coupling reaction was stirred at rt. Purification by FCC (70% Et_2O in pentane) gave bipyridine **4** as a yellow oil (34.6 mg, 74%).

^{1}H NMR (500 MHz, Chloroform-*d*) δ 8.80 – 8.61 (m, 3H), 7.95 – 7.86 (m, 2H), 7.85 – 7.78 (m, 2H), 7.34 (ddd, *J* = 6.4, 4.8, 2.3 Hz, 1H) ppm.

^{13}C NMR (126 MHz, Chloroform-*d*) δ 154.8, 150.6, 150.3, 146.5, 137.2, 123.9, 121.2, 121.0 ppm.

Spectra are consistent with literature data.[8]

4-Methyl-4'-(trifluoromethyl)-2,2'-bipyridine 5

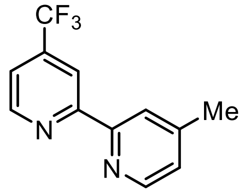

Product **5** was synthesised *via* General procedure D using 2-bromo-4-methylpyridine (50.1 µL, 0.45 mmol) and sulfonium salt **1c** (148.8 mg, 0.3 mmol). The Grignard reagent was formed at 30 °C for 2 h and the ligand coupling reaction was stirred at rt. Purification by FCC (5% Et_2O in pentane) gave bipyridine **5** as a white solid (46.2 mg, 64%).

TLC: R_f = 0.18 (10% Et_2O in pentane).

^{1}H NMR (500 MHz, Chloroform-*d*) δ 8.84 (dt, *J* = 5.0, 0.7 Hz, 1H, NC*H*CHCCF$_3$), 8.68 (dt, *J* = 1.7, 0.8 Hz, 1H, CF$_3$CCHC), 8.56 (dd, *J* = 4.9, 0.8 Hz, 1H, CH$_3$CCHC*H*), 8.27 (dt, *J* = 1.7, 0.8 Hz, 1H, CH$_3$CC*H*), 7.51 (ddd, *J* = 5.0, 1.8, 0.8 Hz, 1H, NCHC*H*CCF$_3$), 7.19 (ddd, *J* = 5.0, 1.7, 0.8 Hz, 1H, CH$_3$CC*H*CH), 2.46 (s, 3H, CH$_3$) ppm.

^{13}C NMR (126 MHz, Chloroform-*d*) δ 157.9 (C), 154.3 (C), 150.1 (N*C*HCHCCF$_3$), 149.3 (CH$_3$CCH*C*H), 148.6 (CH$_3$*C*), 139.5 (q, *J* = 34.2 Hz, CF$_3$*C*), 125.6 (CH$_3$C*C*HCH), 123.1 (q, *J* = 273.2 Hz, CF$_3$), 122.3 (CH$_3$C*C*H), 119.2 (q, *J* = 3.5 Hz, CF$_3$C*C*HCH), 117.2 (q, *J* = 3.6 Hz, CF$_3$C*C*HC), 21.4 ppm.

^{19}F NMR (470 MHz, CDCl$_3$) δ -64.8 ppm.

Spectra are consistent with literature data.[9]

4-Fluoro-4'-(trifluoromethyl)-2,2'-bipyridine 6

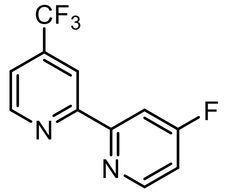

Product **6** was synthesised *via* General procedure D using 2-bromo-4-fluoropyridine (46.6 µL, 0.45 mmol) and sulfonium salt **1c** (148.8 mg, 0.3 mmol). The Grignard reagent was formed at rt for 1 h and the ligand coupling reaction was stirred at rt. Purification by FCC (7.5% Et_2O in pentane) gave bipyridine **6** as a yellow oil (52.8 mg, 73%).

TLC: R_f = 0.55 (10% Et_2O in pentane).

^{1}H NMR (500 MHz, Chloroform-*d*) δ 8.84 (dt, *J* = 5.0, 0.7 Hz, 1H, NC*H*CHCCF$_3$), 8.69 (dt, *J* = 1.7, 0.8 Hz, 1H, F$_3$CCCH), 8.66 (dd, *J* = 8.4, 5.5 Hz, 1H, NC*H*CHCF), 8.20 (dd, *J* = 10.2, 2.5 Hz, 1H, FCCHC), 7.59 – 7.51 (m, 1H, NCHC*H*CCF$_3$), 7.10 (ddd, *J* = 8.1, 5.5, 2.5 Hz, 1H, FCC*H*CH) ppm.

^{13}C NMR (126 MHz, Chloroform-*d*) δ 169.7 (d, *J* = 262.3 Hz, CF), 158.2 (d, *J* = 7.4 Hz, FCCH*C*), 156.5 (d, *J* = 3.8 Hz, N*C*CHCCF$_3$), 151.8 (d, *J* = 6.9 Hz, FCCH*C*HN), 150.3 (N*C*HCHCCF$_3$), 139.6 (q, *J* = 34.2 Hz, CF$_3$*C*), 123.0 (q, *J* = 273.4 Hz, CF$_3$), 119.8 (q, *J* = 3.4 Hz, CF$_3$C*C*H), 117.3 (q, *J* = 3.7 Hz, CF$_3$C*C*H), 112.4 (d, *J* = 16.8 Hz, FC*C*HC), 109.3 (d, *J* = 18.7 Hz, FC*C*HCH) ppm.

^{19}F NMR (470 MHz, Chloroform-*d*) δ -64.9 (CF$_3$), -101.3 (app. dt, *J* = 10.3, 8.2 Hz, CF) ppm.

HRMS (ESI-TOF) *m/z*: $[M+H]^+$ Calcd for $C_{11}H_7F_4N_2$: 243.0540; found: 243.0539 ppm.

1-(5-Methoxypyridin-2-yl)isoquinoline 7

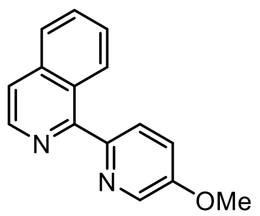

Product **7** was synthesised *via* General procedure D using 2-bromo-5-methoxypyridine (56 µL, 0.45 mmol) and sulfonium salt **1b** (143.3 mg, 0.3 mmol). The Grignard reagent was formed at rt for 3 h and the ligand coupling reaction was stirred at rt. Purification by FCC (50% Et$_2$O in pentane) gave bipyridine **7** as a white solid (55.3 mg, 78%).

TLC: R_f = 0.1 (50% Et$_2$O in pentane).

^{1}H NMR (500 MHz, Chloroform-*d*) δ 8.67 (dd, *J* = 8.6, 1.0 Hz, 1H, NCCCH), 8.60 (d, *J* = 5.6 Hz, 1H, NCH), 8.49 (d, *J* = 2.9 Hz, 1H, NC*H*COCH$_3$), 7.99 (dd, *J* = 8.6, 0.7 Hz, 1H, CH$_3$OCCHC*H*), 7.88 – 7.83 (m, 1H, NCCCHC*H*), 7.72 – 7.65 (m, 2H, ArH), 7.59 (ddd, *J* = 8.3, 6.8, 1.3 Hz, 1H, NCCCHCHC*H*), 7.41 (dd, *J* = 8.6, 3.0 Hz, 1H, CH$_3$OCC*H*), 3.97 (s, 3H. OCH$_3$) ppm.

^{13}C NMR (126 MHz, Chloroform-*d*) δ 157.4 (N*C*C), 155.7 (*C*OCH$_3$), 151.1 (C), 142.0 (NCH), 137.4 (C), 136.4 (N*C*HCO), 130.1 (CH), 128.1 (NCH*C*H), 127.6 (NCCCHCH*C*H*)*, 127.0 (NCCCH*C*H*)*, 126.8 (C), 125.9 (CH$_3$OCCH*C*H), 121.4 (CH$_3$OC*C*H), 120.9 (CH), 55.9 (OCH$_3$) ppm.

HRMS (ESI-TOF) *m/z*: $[M+H]^+$ Calcd for $C_{15}H_{13}N_2O$: 237.1022; found: 237.1023.

5'-Bromo-2,3'-bipyridine 8

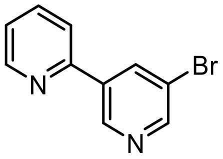

Product **8** was synthesised *via* General procedure D using 3,5-dibromopyridine (106.6 mg, 0.45 mmol) and sulfonium salt **1a** (128.2 mg, 0.3 mmol). The Grignard reagent was formed at rt for 1 h and the ligand coupling reaction was stirred at rt. Purification by FCC (40% Et_2O in pentane) gave bipyridine **8** as a yellow solid (43.7 mg, 62%).

TLC: R_f = 0.22 (40% Et_2O in pentane)

^{1}H NMR (500 MHz, Chloroform-*d*) δ 9.09 (d, *J* = 1.9 Hz, 1H), 8.72 (dt, *J* = 4.8, 1.3 Hz, 1H), 8.70 (d, *J* = 2.2 Hz, 1H), 8.51 (t, *J* = 2.0 Hz, 1H), 7.81 (dd, *J* = 7.7, 1.8 Hz, 1H), 7.74 (d, *J* = 7.7 Hz, 1H), 7.32 (ddd, *J* = 7.3, 4.7, 0.8 Hz, 1H).

^{13}C NMR (126 MHz, Chloroform-*d*) δ 153.4, 151.0, 150.4, 146.3, 137.3, 137.1, 136.5, 123.5, 121.3, 120.9.

Spectra were consistent with literature data.[10]

6-Bromo-2,3'-bipyridine 9

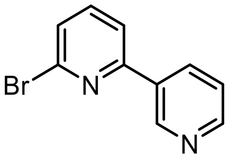

Product **9** was synthesised *via* General procedure D using 3-iodopyridine (92.3 mg, 0.45 mmol) and sulfonium salt **1g** (151.9 mg, 0.3 mmol). The Grignard reagent was formed at rt for 45 min and the ligand coupling reaction was stirred at rt. Purification by FCC (80% Et_2O in pentane) gave bipyridine **9** as a yellow solid (46.8 mg, 66%).

TLC: R_f = 0.19 (80% Et_2O in pentane)

^{1}H NMR (500 MHz, Chloroform-*d*) δ 9.15 (d, *J* = 2.3 Hz, 1H), 8.65 (dd, *J* = 4.9, 1.6 Hz, 1H), 8.32 (dt, *J* = 7.9, 2.1 Hz, 1H), 7.70 (d, *J* = 7.7 Hz, 1H), 7.63 (t, *J* = 7.8 Hz, 1H), 7.46 (d, *J* = 7.7 Hz, 1H), 7.39 (dd, *J* = 8.0, 4.8 Hz, 1H).

^{13}C NMR (126 MHz, Chloroform-*d*) δ 156.0, 150.6, 148.2, 142.7, 139.3, 134.6, 133.4, 127.3, 123.8, 119.2.

Spectra were consistent with literature data.[11]

6'-Bromo-6-(trifluoromethyl)-2,3'-bipyridine 10

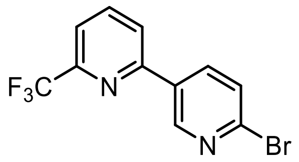

Product **10** was synthesised *via* General procedure D using 2,5-dibromopyridine (106.6 mg, 0.45 mmol) and sulfonium salt **1i** (148.6 mg, 0.3 mmol). The Grignard reagent was formed at rt for 1 h and the ligand coupling reaction was stirred at rt. Purification by FCC (20% Et_2O in pentane) gave bipyridine **10** as a yellow solid (45.0 mg, 50%).

TLC: R_f = 0.19 (20% Et_2O in pentane)

^{1}H NMR (400 MHz, Chloroform-*d*) δ 8.96 (d, *J* = 2.5 Hz, 1H, CHN), 8.29 (dd, *J* = 8.3, 2.7 Hz, 1H), 7.99 (t, *J* = 7.8 Hz, 1H), 7.93 (d, *J* = 7.6 Hz, 1H), 7.69 (dd, *J* = 7.5, 1.1 Hz, 1H), 7.61 (dd, *J* = 8.3, 0.5 Hz, 1H).^{13}C NMR (101 MHz, Chloroform-*d*) δ 154.2 (C), 148.9 (q, *J* = 35.0 Hz, *C*CF_3), 148.6 (CH), 143.7 (CBr), 138.8 (CH), 137.3 (CH), 132.8 (C), 128.5 (CH), 122.8 (CH), 121.4 (q, *J* = 276.6 Hz, CF_3), 119.8 (q, *J* = 2.7 Hz, CH).

^{19}F NMR (282 MHz, Chloroform-*d*) δ -68.2.

HRMS (ESI-TOF) *m/z*: $[M+H]^+$ Calcd for $C_{11}H_7BrF_3N_2$: 302.9739, 304.9719; Found: 302.9741, 304.9721.

5-Chloro-2,3'-bipyridine 11

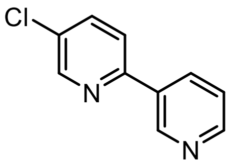

Product **11** was synthesised *via* General procedure D using 3-iodopyridine (92.3 mg, 0.45 mmol) and sulfonium salt **1l** (138.6 mg, 0.3 mmol). The Grignard reagent was formed at rt for 45 min and the ligand coupling reaction was stirred at rt. Purification by FCC (80% Et_2O in pentane) gave bipyridine **11** as a white solid (31.9 mg, 56%).

TLC: R_f = 0.27 (80% Et_2O in pentane)

^{1}H NMR (400 MHz, Chloroform-*d*) δ 9.17 – 9.14 (m, 1H), 8.67 – 8.64 (m, 2H), 8.28 (dt, *J* = 7.9, 2.0 Hz, 1H), 7.76 (dd, *J* = 8.4, 2.5 Hz, 1H), 7.70 (dd, *J* = 8.6, 0.6 Hz, 1H), 7.40 (ddd, *J* = 7.8, 4.7, 0.6 Hz, 1H).

^{13}C NMR (101 MHz, Chloroform-*d*) δ 153.0, 150.3, 149.1, 148.2, 136.8, 134.3, 133.9, 131.6, 123.8, 121.2.

Spectra were consistent with literature data.[12]

5,5'-Dibromo-2,3'-bipyridine 12

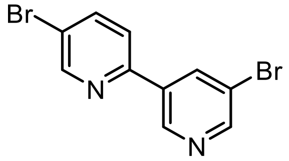

Product **12** was synthesised *via* General procedure D using 3,5-dibromopyridine (106.6 mg, 0.45 mmol) and sulfonium salt **1k** (151.9 mg, 0.3 mmol). The Grignard reagent was formed at rt for 1 h and the ligand coupling reaction was stirred at 45 °C for 3.5 h. Purification by FCC (40% Et_2O in pentane) gave bipyridine **12** as a white solid (63.5 mg, 67%).

TLC: R_f = 0.23 (40% Et_2O in pentane)

^1H NMR (400 MHz, Chloroform-*d*) δ 9.07 (d, *J* = 1.9 Hz, 1H), 8.77 (d, *J* = 2.3 Hz, 1H), 8.72 (d, *J* = 2.3 Hz, 1H), 8.49 (t, *J* = 2.1 Hz, 1H), 7.93 (dd, *J* = 8.5, 2.4 Hz, 1H), 7.65 (d, *J* = 8.3 Hz, 1H).

^{13}C NMR (101 MHz, Chloroform-*d*) δ 151.8 (C), 151.5 (CH), 151.3 (CH), 146.0 (CH), 139.9 (CH), 136.9 (CH), 135.4 (C), 121.8 (CH), 121.4 (C), 121.0 (C).

HRMS (ESI-TOF) *m/z*: $[M+H]^+$ Calcd for $C_{10}H_7Br_2N_2$: 312.8970, 314.8950, 316.8931; Found: 312.8971, 314.8952, 316.8930.

[2,3'-Bipyridine]-5-carbonitrile 13

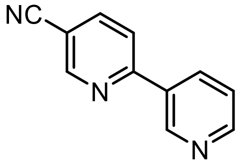

Product **13** was synthesised *via* General procedure D using 3-iodopyridine (92.3 mg, 0.45 mmol) and sulfonium salt **1j** (135.7 mg, 0.3 mmol). The Grignard reagent was formed at rt for 45 min and the ligand coupling reaction was stirred at rt. Purification by FCC (80% Et_2O in pentane) gave bipyridine **13** as a yellow solid (28.4 mg, 52%).

TLC: R_f = 0.14 (80% Et_2O in pentane)

^1H NMR (500 MHz, Chloroform-*d*) δ 9.25 (s, 1H), 8.97 (d, *J* = 2.0 Hz, 1H), 8.73 (d, *J* = 4.8 Hz, 1H), 8.37 (dt, *J* = 7.9, 1.9 Hz, 1H), 8.06 (dd, *J* = 8.3, 2.0 Hz, 1H), 7.89 (d, *J* = 8.4 Hz, 1H), 7.46 (dd, *J* = 8.0, 4.8 Hz, 1H).

^{13}C NMR (126 MHz, Chloroform-*d*) δ 158.1 (C), 152.8 (CH), 151.5 (CH), 148.7 (CH), 140.3 (CH), 134.9 (CH), 133.1 (C), 124.0 (CH), 120.2 (CH), 116.7 (CN), 108.9 (*C*CN).

HRMS (ESI-TOF) *m/z*: $[M+H]^+$ Calcd for $C_{11}H_8N_3$: 182.0713; Found: 182.0714.

5-Methoxy-2,3'-bipyridine 14

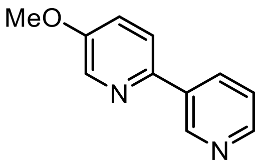

Product **14** was synthesised *via* General procedure D using 3-iodopyridine (92.3 mg, 0.45 mmol) and sulfonium salt **1f** (137.2 mg, 0.3 mmol). The Grignard reagent was formed at rt for 45 min and the ligand coupling reaction was stirred at rt. Purification by FCC (80% Et_2O in pentane) gave bipyridine **14** as a white solid (23.1 mg, 41%).

TLC: R_f = 0.10 (80% Et_2O in pentane)

^{1}H NMR (500 MHz, Chloroform-*d*) δ 9.12 (d, *J* = 2.3 Hz, 1H), 8.59 (dd, *J* = 4.8, 1.6 Hz, 1H), 8.41 (d, *J* = 2.9 Hz, 1H), 8.25 (dt, *J* = 8.1, 2.1 Hz, 1H), 7.68 (d, *J* = 8.5 Hz, 1H), 7.36 (dd, *J* = 7.9, 4.7 Hz, 1H), 7.29 (dd, *J* = 8.7, 3.0 Hz, 1H), 3.90 (s, 3H, OMe).

^{13}C NMR (126 MHz, Chloroform-*d*) δ 155.5 (C), 149.3 (CH), 147.9 (CH), 147.4 (C), 137.9 (CH), 134.7 (C), 133.9 (CH), 123.7 (CH), 121.3 (CH), 121.0 (CH), 55.8 (OMe).

HRMS (ESI-TOF) *m/z*: $[M+H]^+$ Calcd for $C_{11}H_{11}N_2O$: 187.0866; Found: 187.0867.

2'-Fluoro-2,4'-bipyridine 15

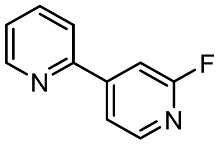

Product **15** was synthesised *via* General procedure D using 4-bromo-2-fluoropyridine (46.2 µL, 0.45 mmol) and sulfonium salt **1a** (128.2 mg, 0.3 mmol). The Grignard reagent was formed at rt for 30 min and the ligand coupling reaction was stirred at rt. Purification by FCC (70% Et_2O in pentane) gave bipyridine **15** as a yellow oil (46.0 mg, 88%).

^{1}H NMR (500 MHz, Chloroform-*d*) δ 8.74 (ddd, *J* = 4.8, 1.8, 1.0 Hz, 1H), 8.31 (dt, *J* = 5.3, 0.7 Hz, 1H), 7.83 (td, *J* = 7.7, 1.8 Hz, 1H), 7.80 – 7.75 (m, 2H), 7.62 – 7.52 (m, 1H), 7.37 (ddd, *J* = 7.4, 4.8, 1.3 Hz, 1H) ppm.

^{13}C NMR (126 MHz, Chloroform-*d*) δ 164.9 (d, *J* = 237.9 Hz, CF), 153.5 (d, *J* = 3.8 Hz), 152.2 (d, *J* = 8.1 Hz), 150.3, 148.3 (d, *J* = 15.2 Hz), 137.3, 124.5, 121.1, 119.0 (d, *J* = 4.2 Hz), 107.1 (d, *J* = 39.1 Hz) ppm.

^{19}F NMR (470 MHz, Chloroform-*d*) δ -67.8 ppm.

Spectra are consistent with literature data.[10]

2'-Fluoro-6-methyl-2,4'-bipyridine 16

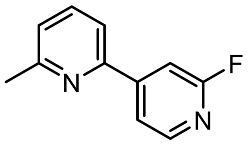

Product **16** was synthesised *via* General procedure D using 4-bromo-2-fluoropyridine (46.2 µL, 0.45 mmol) and sulfonium salt **1h** (132.4 mg, 0.3 mmol). The Grignard reagent was formed at 0 °C for 30 min and the ligand coupling reaction was stirred at 0 °C. Purification by FCC (20% Et_2O in pentane) gave bipyridine **16** as a white solid (38.7 mg, 69%).

^{1}H NMR (500 MHz, Chloroform-*d*) δ 8.30 (d, *J* = 5.2 Hz, 1H), 7.77 (dt, *J* = 5.3, 1.7 Hz, 1H), 7.71 (t, *J* = 7.7 Hz, 1H), 7.61 – 7.55 (m, 2H), 7.23 (d, *J* = 7.6 Hz, 1H), 2.64 (s, 3H) ppm.

^{13}C NMR (126 MHz, Chloroform-*d*) δ 164.9 (d, *J* = 237.9 Hz, CF), 159.3, 152.9 (d, *J* = 3.8 Hz), 152.6 (d, *J* = 8.2 Hz), 148.2 (d, *J* = 15.2 Hz), 137.4, 124.1, 119.1 (d, *J* = 4.2 Hz), 118.2, 107.2 (d, *J* = 38.9 Hz), 24.8 ppm.

^{19}F NMR (376 MHz, Chloroform-*d*) δ -68.1 ppm.

Spectra are consistent with literature data.[10]

1-(2-Fluoropyridin-4-yl)isoquinoline 17

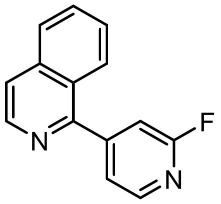

Product **17** was synthesised *via* General procedure D using 4-bromo-2-fluoropyridine (46.2 µL, 0.45 mmol) and sulfonium salt **1b** (143.3 mg, 0.3 mmol). The Grignard reagent was formed at 0 °C for 30 min and the ligand coupling reaction was stirred at 0 °C. Purification by FCC (50% Et_2O in pentane) gave bipyridine **17** as a white solid (67.2 mg, quant. yield).

The reaction was scaled up and product **17** was synthesized via General procedure D using 4-bromo-2-fluoropyridine (1.13 mL, 11.0 mmol) and sulfonium salt **1b** (3.50 g, 7.3 mmol). Purification by FCC (50% Et2O in pentane) gave bipyridine **17** as a white solid (1.57 g, 96%).

TLC: R_f = 0.33 (35% Et_2O in pentane).

^{1}H NMR (500 MHz, Chloroform-*d*) δ 8.64 (d, *J* = 5.6 Hz, 1H, NCH), 8.40 (dt, *J* = 5.1, 0.8 Hz, 1H, FCNCH), 8.02 (dt, *J* = 8.6, 1.0 Hz, 1H, NCHCHCC*H*), 7.97 – 7.92 (m, 1H, ArH), 7.79 – 7.72 (m, 2H, ArH), 7.62 (ddd, *J* = 8.2, 6.8, 1.2 Hz, 1H, NCCCHC*H*), 7.53 (dd, *J* = 5.1, 2.0 Hz, 1H, NCCC*H*CHN), 7.27 (d, *J* = 1.9 Hz, FCCH) ppm.

^{13}C NMR (126 MHz, Chloroform-*d*) δ 163.9 (d, *J* = 239.4 Hz, CF), 156.7 (d, *J* = 3.3 Hz, FCCHC*C*), 152.8 (d, *J* = 8.1 Hz, FCCH*C*), 147.9 (d, *J* = 15.1 Hz, FCN*C*H), 142.5 (NCH), 136.9 (C), 130.6 (CH), 128.1 (NCCCHC*H*), 127.4 (CH), 126.11 (NCC*C*H), 126.10 (C), 122.5 (d, *J* = 4.3 Hz, FCNCHC*H*), 121.5 (CH), 110.7 (d, *J* = 38.1 Hz, FC*C*H) ppm.

^{19}F NMR (470 MHz, Chloroform-*d*) δ -67.4 ppm.

HRMS (ESI-TOF) *m/z*: $[M+H]^+$ Calcd for $C_{14}H_{10}FN_2$: 225.0823; found: 225.0820.

1-(2-Methoxypyridin-4-yl)isoquinoline 18

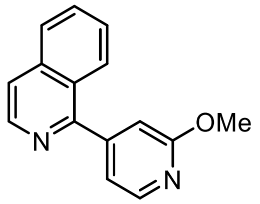

Product **18** was synthesised *via* General procedure D using 4-bromo-2-methoxypyridine (84.6 mg, 0.45 mmol) and sulfonium salt **1b** (143.3 mg, 0.3 mmol). The Grignard reagent was formed at 40 °C for 2 h and the ligand coupling reaction was stirred at rt. Purification by FCC (25% Et_2O in pentane) gave bipyridine **18** as a white solid (44.7 mg, 63%).

TLC: R_f = 0.11 (20% Et_2O in pentane).

^{1}H NMR (500 MHz, Chloroform-*d*) δ 8.61 (d, *J* = 5.7 Hz, 1H, NCH), 8.33 (dd, *J* = 5.2, 0.8 Hz, 1H, CH_3OCNC*H*), 8.05 (dd, *J* = 8.6, 1.0 Hz, 1H, NCCCH), 7.89 (dt, *J* = 8.2, 1.0 Hz, 1H, NCHCHCC*H*), 7.79 – 7.65 (m, 2H, ArH), 7.55 (ddd, *J* = 8.3, 6.9, 1.3 Hz, 1H, NCHCHCCHC*H*), 7.19 (dd, *J* = 5.2, 1.4 Hz, 1H, OCNCHC*H*), 7.05 (dd, *J* = 1.4, 0.8 Hz, 1H, OCCH), 4.02 (s, 3H, OCH_3) ppm.

^{13}C NMR (126 MHz, Chloroform-*d*) δ 164.5 (C), 158.2 (C), 150.0 (C), 147.2 (OCN*C*H), 142.4 (NCH), 136.9 (app. d, *J* = 1.3 Hz, CH_3O*C*), 130.4 (CH), 127.8 (NCHCHCCHC*H*), 127.3 (NCHCHC*C*H), 126.8 (NCC*C*H), 126.4 (C), 121.0 (CH), 118.1 (OCNCHC*H*), 111.9 (OC*C*H), 53.8 (OCH_3) ppm.

HRMS (ESI-TOF) *m/z*: $[M+H]^+$ Calcd for $C_{15}H_{14}N_2O$: 237.1022; found: 237.1020.

4-(Trifluoromethyl)-2,4'-bipyridine 19

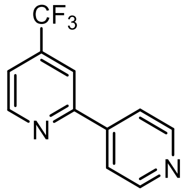

Product **19** was synthesised *via* General procedure D using 4-iodopyridine (93 mg, 0.45 mmol) and sulfonium salt **1c** (148.8 mg, 0.3 mmol). The Grignard reagent was formed at 0 °C for 30 min and the ligand coupling reaction was stirred at 0 °C warming to rt. Purification by FCC (50% Et_2O in pentane) gave bipyridine **19** as a yellow oil (37.0 mg, 55%).

TLC: R_f = 0.27 (25% Et_2O in pentane).

^{1}H NMR (500 MHz, Chloroform-*d*) δ 8.93 (dt, *J* = 5.0, 0.7 Hz, 1H, NC*H*CHCCF$_3$), 8.83 – 8.71 (m, 2H, ArH), 7.99 (dt, *J* = 1.6, 0.7 Hz, 1H, CF$_3$CCH), 7.96 – 7.91 (m, 2H, ArH), 7.56 (ddd, *J* = 5.0, 1.6, 0.7 Hz, 1H, NCHC*H*CCF$_3$) ppm.

^{13}C NMR (126 MHz, Chloroform-*d*) δ 156.3 (N*C*C), 151.3 (N*C*HCHCCF$_3$), 150.8 (CH), 145.1 (NC*C*), 139.7 (q, *J* = 34.2 Hz, CF$_3$*C*), 122.8 (q, *J* = 273.2 Hz, CF$_3$), 121.2 (CH), 119.4 (q, *J* = 3.5 Hz, CF$_3$C*C*H), 116.6 (q, *J* = 3.7 Hz, CF$_3$C*C*H) ppm.

^{19}F NMR (470 MHz, $CDCl_3$) δ -64.8 ppm.

HRMS (ESI-TOF) *m/z*: $[M+H]^+$ Calcd for $C_{11}H_8F_3N_2$: 225.0634; found: 225.0635.

2'-Methoxy-4-(trifluoromethyl)-2,4'-bipyridine 20

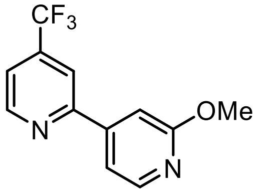

Product **20** was synthesised *via* General procedure D using 4-bromo-2-methoxypyridine (84.6 mg, 0.45 mmol) and sulfonium salt **1c** (148.8 mg, 0.3 mmol). The Grignard was formed at 40 °C for 2 h and the ligand coupling reaction was stirred at rt. Purification by FCC (15% Et_2O in pentane) gave bipyridine **20** as a white solid (49.0 mg, 64%).

TLC: R_f = 0.46 (20% Et_2O in pentane).

^{1}H NMR (500 MHz, Chloroform-*d*) δ 8.91 (dt, *J* = 5.0, 0.8 Hz, 1H, NC*H*CHCCF$_3$), 8.31 (dd, *J* = 5.4, 0.8 Hz, 1H, OCNCH), 7.94 (dt, *J* = 1.6, 0.8 Hz, 1H, F$_3$CCCH), 7.54 (ddd, J = 5.0, 1.7, 0.8 Hz,

NCHC*H*CCF$_3$), 7.50 (dd, J = 5.4, 1.5 Hz, OCNCHC*H*), 7.37 (dd, *J* = 1.5, 0.7 Hz, 1H, OCCH), 4.00 (s, 3H, OCH$_3$) ppm.

^{13}C NMR (126 MHz, Chloroform-*d*) δ 165.4 (H$_3$CO*C*), 156.4 (F$_3$CCCHC*N*), 151.1 (N*C*HCHCCF$_3$), 148.0 (OCN*C*H), 147.98 (NC*C*), 139.6 (q, *J* = 34.3 Hz, CF$_3$*C*), 122.9 (q, *J* = 273.2 Hz, CF$_3$), 119.3 (q, *J* = 3.6 Hz, CF$_3$C*C*HCH), 116.7 (q, *J* = 3.7 Hz, CF$_3$C*C*HC), 114.6 (OCNCHC*H*), 108.7 (OC*C*H), 53.9 (OCH$_3$) ppm.

HRMS (ESI-TOF) *m/z*: [M+H]$^+$ Calcd for $C_{12}H_{10}F_3N_2O$: 254.0661; found: 254.0676.

2',6'-Dichloro-3-methyl-2,4'-bipyridine 21

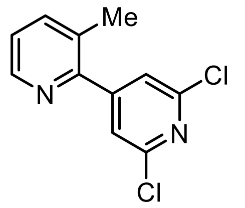

Product **21** was synthesised *via* General procedure E using 2,6-dichloropyridine (50.1 μL, 0.45 mmol) and sulfonium salt **1d** (132.4 mg, 0.3 mmol). The Grignard reagent was formed at rt for 10 min and the ligand coupling reaction was stirred at rt. Purification by FCC (5% Et$_2$O in pentane) gave bipyridine **21** as a white solid (46.2 mg, 64%).

TLC: R_f = 0.35 (20% Et$_2$O in pentane).

^{1}H NMR (500 MHz, Chloroform-*d*) δ 8.54 (ddd, *J* = 4.7, 1.6, 0.7 Hz, 1H, NCH), 7.64 (ddd, *J* = 7.8, 1.7, 0.8 Hz, 1H, NCHCHC*H*), 7.45 (s, 2H, ArH), 7.29 (dd, *J* = 7.8, 4.7 Hz, 1H, NCHC*H*), 2.39 (s, 3H, CH$_3$) ppm.

^{13}C NMR (126 MHz, Chloroform-*d*) δ 153.5 (C), 153.4 (C), 150.7 (CH$_3$*C*), 147.8 (NCH), 139.4 (NCHCH*C*H), 131.4 (NCCl), 124.1 (NCH*C*H), 123.2 (NCCl*C*H), 19.8 (CH$_3$) ppm.

HRMS (ESI-TOF) *m/z*: [M+H]$^+$ Calcd for $C_{11}H_9Cl_2N_2$: 239.0137, 240.0167, 241.0109, 242.0138 and 243.0082; found: 239.0140, 240.0173, 241.0110, 242.0142 and 243.0080.

2'-Fluoro-6-(trifluoromethyl)-2,4'-bipyridine 22

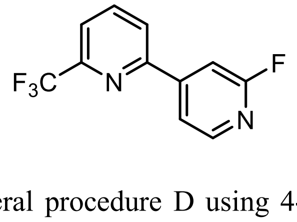

Product **22** was synthesised *via* General procedure D using 4-bromo-2-fluoropyridine (46 μL, 0.45 mmol) and sulfonium salt **1i** (148.6 mg, 0.3 mmol). The Grignard reagent was formed at rt for 1 h and

the ligand coupling reaction was stirred at rt. Purification by FCC (20% Et_2O in pentane) gave bipyridine **22** as a white solid (41.1 mg, 57%).

TLC: R_f = 0.14 (20% Et_2O in pentane).

^{1}H NMR (500 MHz, Chloroform-*d*) δ 8.35 (d, *J* = 5.3 Hz, 1H), 8.04 (t, *J* = 7.8 Hz, 1H), 7.99 (d, *J* = 8.0 Hz, 1H), 7.85 – 7.82 (m, 1H), 7.76 (d, *J* = 7.7 Hz, 1H), 7.62 (s, 1H).

^{13}C NMR (126 MHz, Chloroform-*d*) δ 164.9 (d, *J* = 238.7 Hz, CF), 153.9 (d, *J* = 3.3 Hz, C), 150.5 (d, *J* = 8.3 Hz, C), 148.9 (q, *J* = 35.0 Hz, *C*CF$_3$), 148.7 (d, *J* = 15.2 Hz, CH), 139.0 (CH), 123.5 (CH), 121.3 (q, *J* = 274.4 Hz, CF$_3$), 121.0 (q, *J* = 2.8 Hz, CH), 119.0 (d, *J* = 3.9 Hz, CH), 107.4 (d, *J* = 39.1 Hz, CH).

^{19}F NMR (470 MHz, Chloroform-*d*) δ -67.2, -68.2.

HRMS (ESI-TOF) *m/z*: $[M+H]^+$ Calcd for $C_{11}H_7F_4N_2$: 243.0540; Found: 243.0542.

5-Bromo-2'-fluoro-2,4'-bipyridine 23

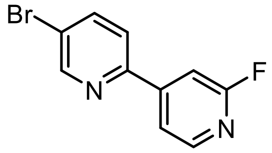

Product **23** was synthesised *via* General procedure D using 4-bromo-2-fluoropyridine (46 µL, 0.45 mmol) and sulfonium salt **1k** (151.9 mg, 0.3 mmol). The Grignard reagent was formed at 45 °C for 1 h and the ligand coupling reaction was stirred at 45 °C for 4.5 h. Purification by FCC (40% Et_2O in pentane) gave bipyridine **23** as a white solid (43.3 mg, 57%).

TLC: R_f = 0.44 (40% Et_2O in pentane).

^{1}H NMR (400 MHz, Chloroform-*d*) δ 8.80 (dd, *J* = 2.3, 0.6 Hz, 1H), 8.33 (d, *J* = 5.3 Hz, 1H), 7.97 (dd, *J* = 8.6, 2.3 Hz, 1H), 7.75 (dt, *J* = 5.4, 1.7 Hz, 1H), 7.69 (d, *J* = 8.3 Hz, 1H), 7.54 (t, *J* = 1.7 Hz, 1H).

^{13}C NMR (126 MHz, Chloroform-*d*) δ 164.9 (d, *J* = 238.4 Hz, CF), 151.9 (d, *J* = 3.8 Hz, C), 151.5 (CH), 151.0 (d, *J* = 8.5 Hz, C), 148.6 (d, *J* = 15.2 Hz, CH), 139.9 (CH), 122.2 (CH), 122.0 (CBr), 118.7 (d, *J* = 3.8 Hz, CH), 107.0 (d, *J* = 39.1 Hz, CH).

^{19}F NMR (376 MHz, Chloroform-*d*) δ -67.4.

HRMS (ESI-TOF) *m/z*: $[M+H]^+$ Calcd for $C_{10}H_7BrFN_2$: 252.9771, 254.9751; Found: 252.9772, 254.9751.

2-(Pyridin-3-yl)pyrimidine 24

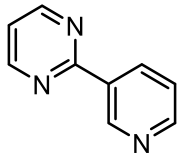

Product **24** was synthesised *via* General procedure D using 3-iodopyridine (92.3 mg, 0.45 mmol) and sulfonium salt **1m** (128.5 mg, 0.3 mmol). The Grignard reagent was formed at rt for 45 min and the ligand coupling reaction was stirred at rt. Purification by FCC (80% Et_2O in pentane) gave bis-heteroaryl **24** as a brown solid (33.1 mg, 70%).

TLC: R_f = 0.24 (80% Et_2O in pentane)

^{1}H NMR (400 MHz, Chloroform-*d*) δ 9.65 (d, *J* = 2.2 Hz, 1H), 8.84 (d, *J* = 4.8 Hz, 2H), 8.74 – 8.66 (m, 2H), 7.45 – 7.40 (m, 1H), 7.26 (t, *J* = 4.9 Hz, 1H).

^{13}C NMR (101 MHz, Chloroform-*d*) δ 163.2, 157.5, 151.5, 150.0, 135.6, 133.2, 123.5, 119.9.

Spectra were consistent with literature data.[13]

2-(6-(Trifluoromethyl)pyridin-2-yl)pyrimidine 25

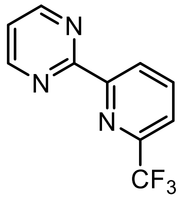

Product **25** was synthesised *via* General procedure D using 2-bromo-6-(trifluoromethyl)pyridine (101.7 mg, 0.45 mmol) and sulfonium salt **1m** (128.5 mg, 0.3 mmol). The Grignard reagent was formed at 45 °C for 2 h and the ligand coupling reaction was stirred at -78 °C. Purification by FCC (60% Et_2O in pentane) gave bis-heteroaryl **25** as an orange solid (27.2 mg, 40%).

TLC: R_f = 0.16 (60% Et_2O in pentane)

^{1}H NMR (400 MHz, Chloroform-*d*) δ 8.97 (d, *J* = 4.8 Hz, 2H), 8.70 (d, *J* = 8.0 Hz, 1H), 8.06 (t, *J* = 7.9 Hz, 1H), 7.81 (d, *J* = 7.8 Hz, 1H), 7.37 (t, *J* = 4.8 Hz, 1H).

^{13}C NMR (101 MHz, Chloroform-*d*) δ 162.8 (C), 158.0 (CH), 155.6 (C), 148.8 (q, *J* = 34.8 Hz, *C*CF_3), 138.6 (CH), 126.5 (CH), 121.7 (q, *J* = 3.0 Hz, CH), 121.6 (d, *J* = 274.6 Hz, CF_3), 121.0 (CH).

^{19}F NMR (376 MHz, Chloroform-*d*) δ -67.5.

HRMS (ESI-TOF) *m/z*: $[M+H]^+$ Calcd for $C_{10}H_7F_3N_3$: 226.0587; Found: 226.0589.

2-Bromo-4-(4-(trifluoromethyl)pyridin-2-yl)pyrimidine 26

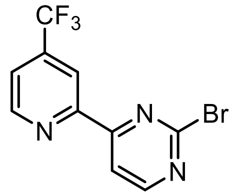

Product **26** was synthesised via General procedure A using 2,4-dibromopyrimidine (71.5 mg, 0.45 mmol) and sulfonium salt **1c** (148.8 mg, 0.3 mmol). The Grignard reagent was formed at -45 °C for 2 h. Purification by FCC (10% Et_2O in pentane) gave bis-heteroaryl **26** as a white solid (58.1 mg, 64%).

TLC: R_f = 0.35 (20% Et_2O in pentane).

^{1}H NMR (500 MHz, Chloroform-*d*) δ 8.90 (dt, *J* = 5.0, 0.7 Hz, 1H), 8.73 (d, *J* = 5.1 Hz, 1H), 8.72 – 8.70 (m, 1H), 8.39 (d, *J* = 5.0 Hz, 1H), 7.67 (ddd, *J* = 5.0, 1.7, 0.8 Hz, 1H).

^{13}C NMR (126 MHz, Chloroform-*d*) δ 164.5 (CBr), 160.8 (CH), 154.0 (C), 153.4 (C), 150.8 (CH), 140.05 (q, *J* = 34.8 Hz, *C*CF_3), 122.7 (t, *J* = 273.8 Hz, CF_3), 121.7 (q, *J* = 3.4 Hz, CH), 118.1 (q, *J* = 3.5 Hz, CH), 116.5 (CH).

^{19}F NMR (470 MHz, Chloroform-*d*) δ -64.8.

HRMS (ESI$^+$): $[M+H]^+$ Calcd for $C_{10}H_5BrF_3N_3$: 303.9692 and 305.9672; found: 303.9694 and 305.9676.

2-(5-(Trifluoromethyl)pyridin-2-yl)pyrazine 27

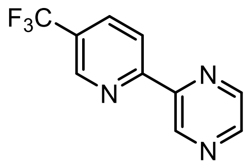

Product **27** was synthesised *via* General procedure E with a slight modification using iodopyrazine (44.4 µL, 0.45 mmol), sulfonium salt **1e** (148.8 mg, 0.3 mmol) and *n*-butylmagnesium chloride (0.23 mL, 2.0 M in ether). The Grignard reagent was formed at -78 °C for 30 min and the ligand coupling reaction was stirred at -78 °C for 8 h. Purification by FCC (25% Et_2O in pentane) gave bis-heteroaryl **27** as a white solid (39.6 mg, 59%).

TLC: R_f = (25% Et_2O in pentane).

^{1}H NMR (500 MHz, Chloroform-*d*) δ 9.69 (d, *J* = 1.4 Hz, 1H, NCHCN), 8.97 (dt, *J* = 2.0, 0.9 Hz, 1H, NC*H*CCF_3), 8.73 – 8.58 (m, 2H, ArH), 8.52 (dt, *J* = 8.3, 0.8 Hz, 1H, F_3CCCHC*H*), 8.15 – 8.03 (m, 1H, F_3CCC*H*CH) ppm.

^{13}C NMR (126 MHz, Chloroform-*d*) δ 157.6 (app. d, *J* = 2.4 Hz, NCCCH), 149.9 (NC*C*CH), 146.6 (q, *J* = 3.9 Hz, N*C*HCCF$_3$), 145.6 (CH), 143.92 (CH), 143.91(N*C*HCN), 134.4 (q, *J* = 3.5 Hz, F$_3$CCHCH), 127.1 (q, *J* = 33.0 Hz, CF$_3$*C*), 123.6 (q, *J* = 272.4 Hz, CF$_3$), 121.2 (CN*C*HCHN) ppm.

^{19}F NMR (470 MHz, Chloroform-*d*) δ -62.5 ppm.

HRMS (ESI-TOF) *m/z*: $[M+H]^+$ Calcd for $C_{10}H_7F_3N_3$: 226.0587; found: 226.0588.

2-(Benzo[*b*]thiophen-2-yl)pyridine 28

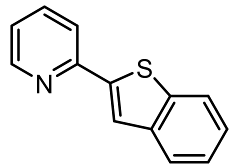

Product **28** was synthesised *via* General procedure E using benzothiophene (60.0 mg, 0.45 mmol) and sulfonium salt **1a** (128.2 mg, 0.3 mmol). The Grignard reagent was formed at rt for 24 h and the ligand coupling reaction was stirred at 45 °C. Purification by FCC (100% Et_2O) gave bis-heteroaryl **28** as a yellow solid (44.3 mg, 70%).

TLC: R_f = 0.05 (100% Et_2O)

^1H NMR (400 MHz, Chloroform-*d*) δ 8.64 (dd, *J* = 4.5, 1.6 Hz, 1H), 7.90 – 7.85 (m, 1H), 7.83 (s, 1H), 7.82 – 7.77 (m, 2H), 7.72 (td, *J* = 7.7, 1.9 Hz, 1H), 7.39 – 7.32 (m, 2H), 7.20 (dd, *J* = 7.4, 4.8 Hz, 1H).

^{13}C NMR (101 MHz, Chloroform-*d*) δ 152.7, 149.8, 144.9, 140.8, 140.6, 136.7, 125.2, 124.6, 124.2, 122.7, 121.2, 119.7.

Spectra were consistent with literature data.[14]

3,5-Dimethyl-4-(pyridin-2-yl)isoxazole 29

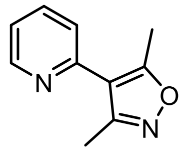

Product **29** was synthesised *via* General procedure D using 4-iodo-3,5-dimethylisoxazole (100.4 mg, 0.45 mmol) and sulfonium salt **1a** (128.2 mg, 0.3 mmol). The Grignard reagent was formed at 45 °C for 2 h and the ligand coupling reaction was stirred at 45 °C. Purification by FCC (30% Et_2O in pentane) gave bis-heteroaryl **29** contaminated with an impurity (8%) as a yellow oil (27.0 mg, 48%).

TLC: R_f = 0.24 (30% Et_2O in pentane).

^1H NMR (400 MHz, Chloroform-*d*) δ 8.69 – 8.65 (m, 1H), 7.74 (td, *J* = 7.8, 1.9 Hz, 1H), 7.32 (d, *J* = 7.9 Hz, 1H), 7.22 (dd, *J* = 7.6, 4.9 Hz, 1H), 2.56 (s, 3H, CH_3), 2.42 (s, 3H, CH_3).

^{13}C NMR (101 MHz, Chloroform-*d*) δ 167.5, 158.8, 151.0, 150.1, 136.6, 123.0, 121.9, 116.2, 12.5 (CH_3), 11.6 (CH_3).

Spectra were consistent with literature data.[15]

2-Phenylpyridine 30

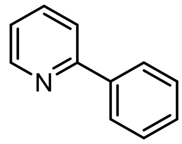

Product **30** was synthesised *via* General procedure D using phenylmagnesium bromide (2.6 M in diethyl ether, 0.17 mL, 0.45 mmol) and sulfonium salt **1a** (128.2 mg, 0.3 mmol). The ligand coupling reaction was stirred at rt. Purification by FCC (80% Et_2O in pentane) gave product **30** as a yellow oil (32.1 mg, 69%).

TLC: R_f = 0.45 (20% Et_2O in pentane).

^{1}H NMR (400 MHz, Chloroform-*d*) δ 8.70 (dt, *J* = 4.7, 1.4 Hz, 1H), 8.02 – 7.98 (m, 2H), 7.78 – 7.70 (m, 2H), 7.52 – 7.45 (m, 2H), 7.45 – 7.39 (m, 1H), 7.23 (ddd, *J* = 6.0, 4.7, 2.4 Hz, 1H).

^{13}C NMR (101 MHz, Chloroform-*d*) δ 157.6, 149.8, 139.5, 136.9, 129.1, 128.9, 127.0, 122.2, 120.7.

Spectra were consistent with literature data.[16]

5. Synthesis of caerulomycin E and A

6-Bromo-4-methoxy-2,2'-bipyridine 36

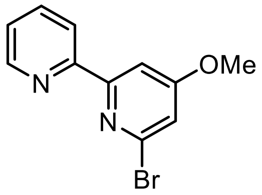

Product **36** was synthesised *via* General procedure D using 2,6-dibromo-4-methoxypyridine (88.0 mg, 0.33 mmol) and sulfonium salt **1a** (128.2 mg, 0.3 mmol). The Grignard reagent was formed at 45 °C for 2 h and the ligand coupling reaction was stirred at rt for 1 h and then at 30 °C for 1 h. Purification by FCC (50% Et_2O in pentane) gave bipyridine **36** as a white solid (73.3 mg, 92%).

TLC: R_f = 0.50 (50% Et_2O in pentane).

^{1}H NMR (400 MHz, Chloroform-*d*) δ 8.64 (dd, *J* = 4.7, 1.7 Hz, 1H), 8.39 (dt, *J* = 8.1, 1.3 Hz, 1H), 7.94 (d, *J* = 2.2 Hz, 1H), 7.80 (td, *J* = 7.7, 1.8 Hz, 1H), 7.31 (ddd, *J* = 7.6, 4.8, 1.2 Hz, 1H), 7.01 (d, *J* = 2.3 Hz, 1H), 3.93 (s, 3H, OMe).

^{13}C NMR (101 MHz, Chloroform-*d*) δ 167.9, 158.4, 154.5, 149.2, 142.3, 137.1, 124.5, 121.8, 114.1, 106.2, 56.0 (OMe).

Spectra were consistent with literature data.[17]

4-Methoxy-[2,2'-bipyridine]-6-carbaldehyde, caerulomycin E 37

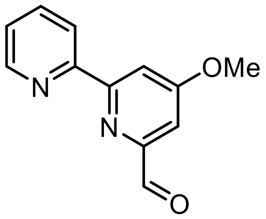

An oven-dried crimptop vial was evacuated and purged with N_2 three times. A solution of *n*-BuLi (0.15 mL, 0.31 mmol, 2.1 M in hexanes) in dry Et_2O (1.0 mL) was prepared and cooled to -78 °C. A solution of 6-bromo-4-methoxy-2,2'-bipyridine (73 mg, 0.28 mmol) in dry Et_2O (0.5 mL) was prepared in a separate oven-dried crimptop vial and added dropwise to the *n*-BuLi solution. Stirring was continued at -78 °C for 30 min. Dimethylformamide (42 µL, 0.54 mmol) in dry Et_2O (0.3 mL) was prepared in a separate oven-dried crimptop vial and then added dropwise to the reaction flask and stirred for 90 min. The reaction was quenched at -78 °C with sat. NH_4Cl (2 mL). The reaction mixture was extracted with Et_2O (3 x 10 mL), the combined organic layers were washed with water (20 mL) and brine (20 mL), and then dried over anhydrous Na_2SO_4. Purification by FCC (10% MeOH in CH_2Cl_2) gave caerulomycin E as a yellow solid (35.6 mg, 60%).

TLC: R_f = 0.08 (10% MeOH in CH_2Cl_2).

^{1}H NMR (400 MHz, Chloroform-*d*) δ 10.13 (s, 1H, CHO), 8.70 (dt, *J* = 4.9, 1.5 Hz, 1H), 8.58 – 8.50 (m, 1H), 8.19 (d, *J* = 2.6 Hz, 1H), 7.86 (td, *J* = 7.7, 1.8 Hz, 1H), 7.49 (d, *J* = 2.4 Hz, 1H), 7.36 (ddd, *J* = 7.5, 4.9, 1.2 Hz, 1H), 4.00 (s, 3H, OMe).

^{13}C NMR (101 MHz, Chloroform-*d*) δ 193.8 (CHO), 167.8, 158.6, 155.1, 154.3, 149.4, 137.2, 124.5, 121.6, 110.7, 107.8, 56.0 (OMe).

Spectra were consistent with literature data.[17]

(*E*)-4-Methoxy-[2,2'-bipyridine]-6-carbaldehyde oxime, caerulomycin A 38

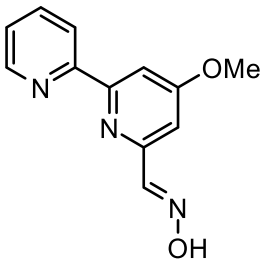

Caerulomycin A was prepared following a procedure adapted from Quéguiner and co-workers.[17] Caerulomycin E (0.025 g, 0.12 mmol), hydroxylamine hydrochloride (0.041 g, 0.59 mmol), pyridine (0.041 mL, 0.50 mmol) and EtOH (0.84 mL) were added to a crimptop vial, which was sealed and heated under reflux for 1 h. Solvent was removed under vacuum and purification by FCC (10% MeOH in CH_2Cl_2) gave caerulomycin A as a white solid (24.1 mg, 90%).

TLC: R_f = 0.07 (10% MeOH in CH_2Cl_2).

^{1}H NMR (400 MHz, DMSO-d_6) δ 11.72 (s, 1H), 8.69 (ddd, J = 4.8, 1.8, 0.9 Hz, 1H), 8.39 (dt, J = 8.0, 1.1 Hz, 1H), 8.15 (s, 1H), 7.96 (td, J = 7.7, 1.8 Hz, 1H), 7.91 (d, J = 2.5 Hz, 1H), 7.48 (ddd, J = 7.5, 4.8, 1.2 Hz, 1H), 7.33 (d, J = 2.5 Hz, 1H), 3.95 (s, 3H, OMe).

^{13}C NMR (101 MHz, DMSO-d_6) δ 166.6, 156.9, 154.5, 153.4, 149.3, 148.8, 137.3, 124.5, 120.7, 106.5, 105.6, 55.6.

Spectra were consistent with literature data.[17]

6. NMR Spectra

^{1}H NMR (500 MHz, $CDCl_3$): 1hi

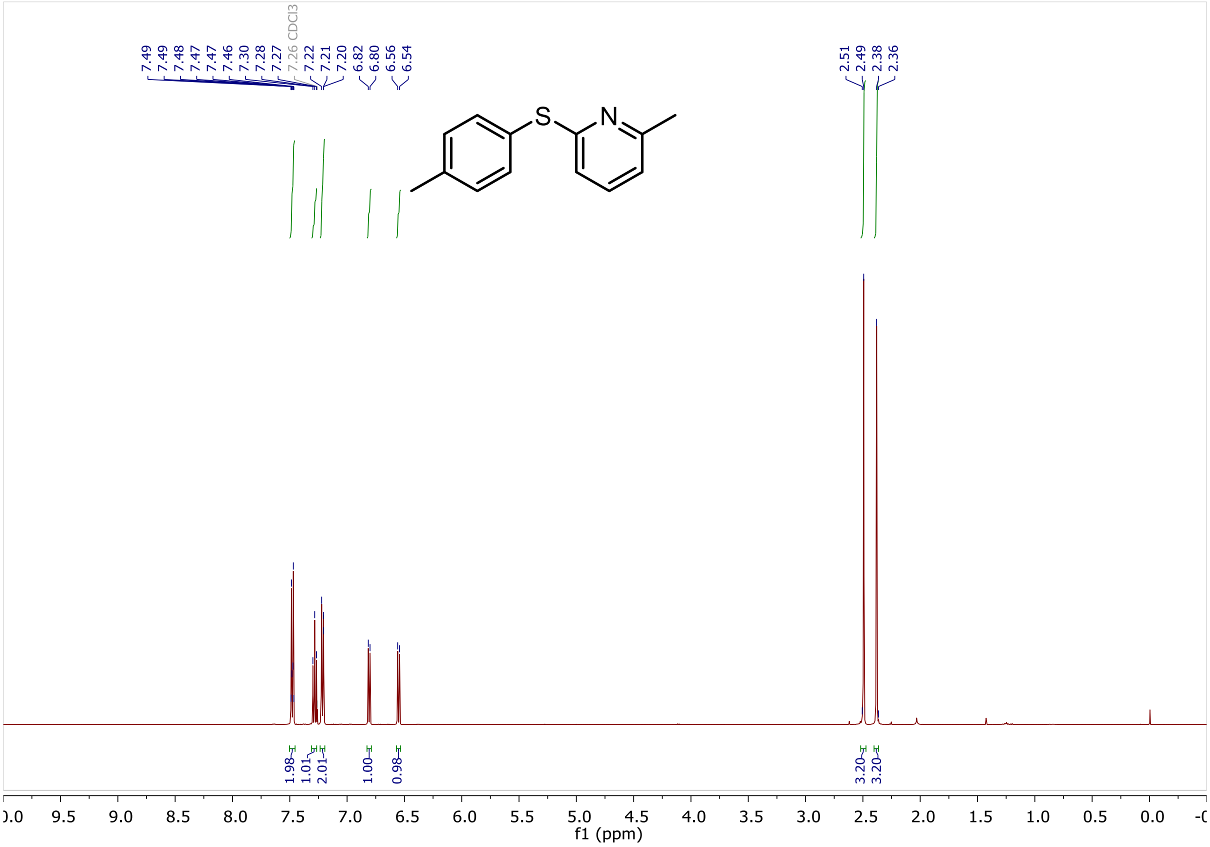

^{13}C NMR (101 MHz, $CDCl_3$): 1hi

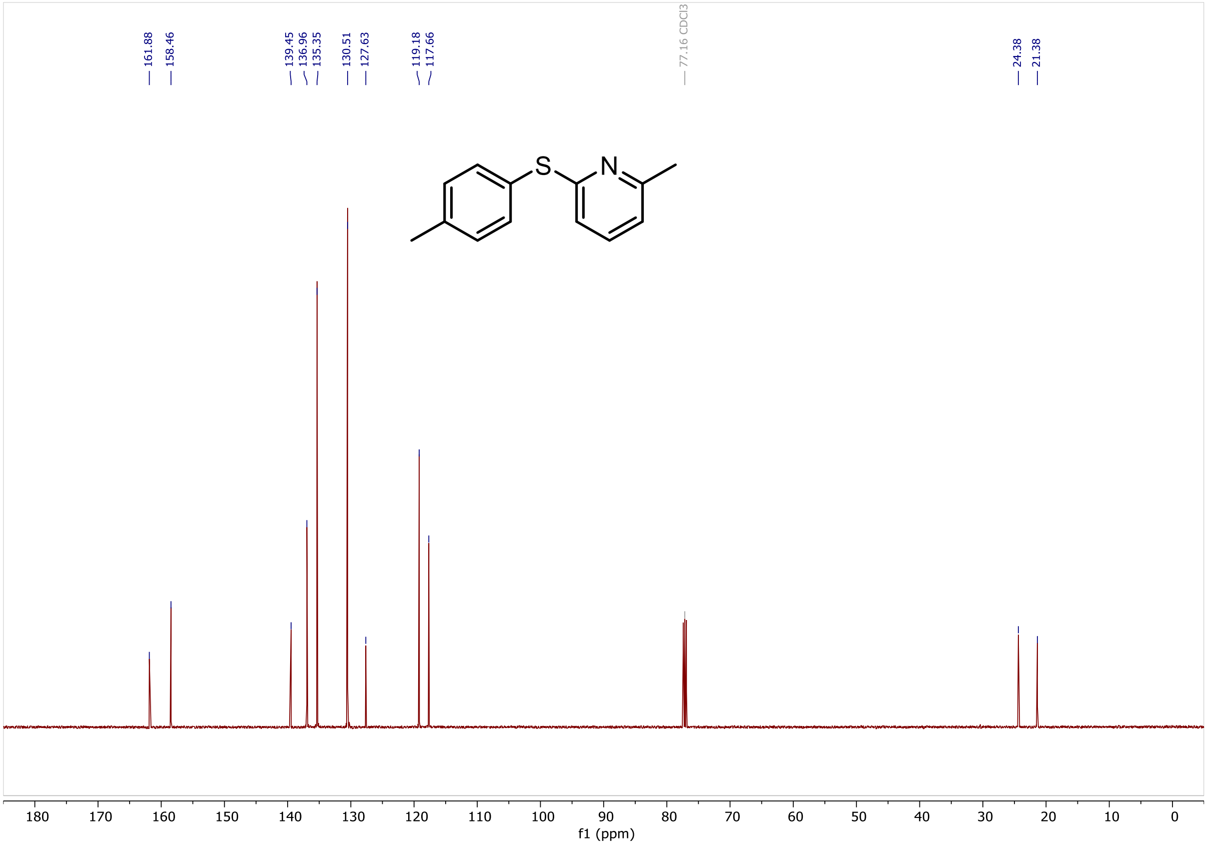

^{1}H NMR (500 MHz, CDCl$_3$): 1ii

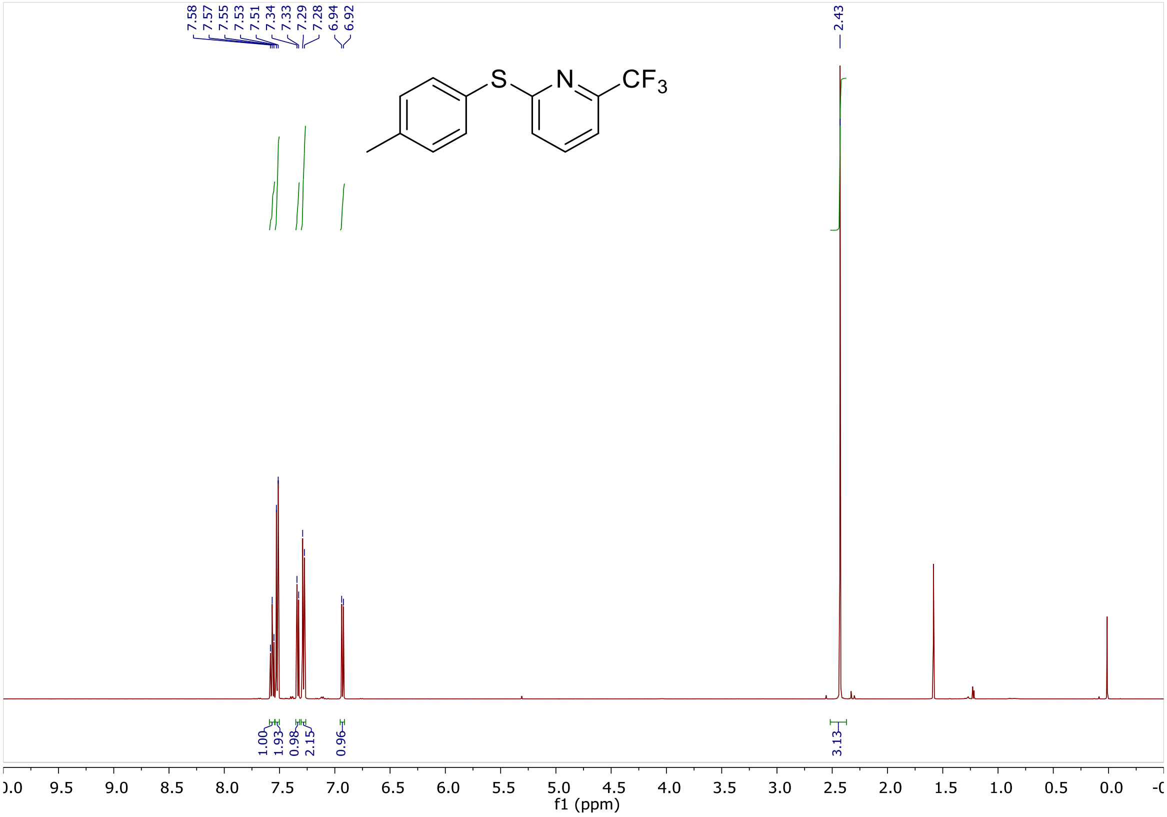

^{13}C NMR (101 MHz, CDCl$_3$): 1ii

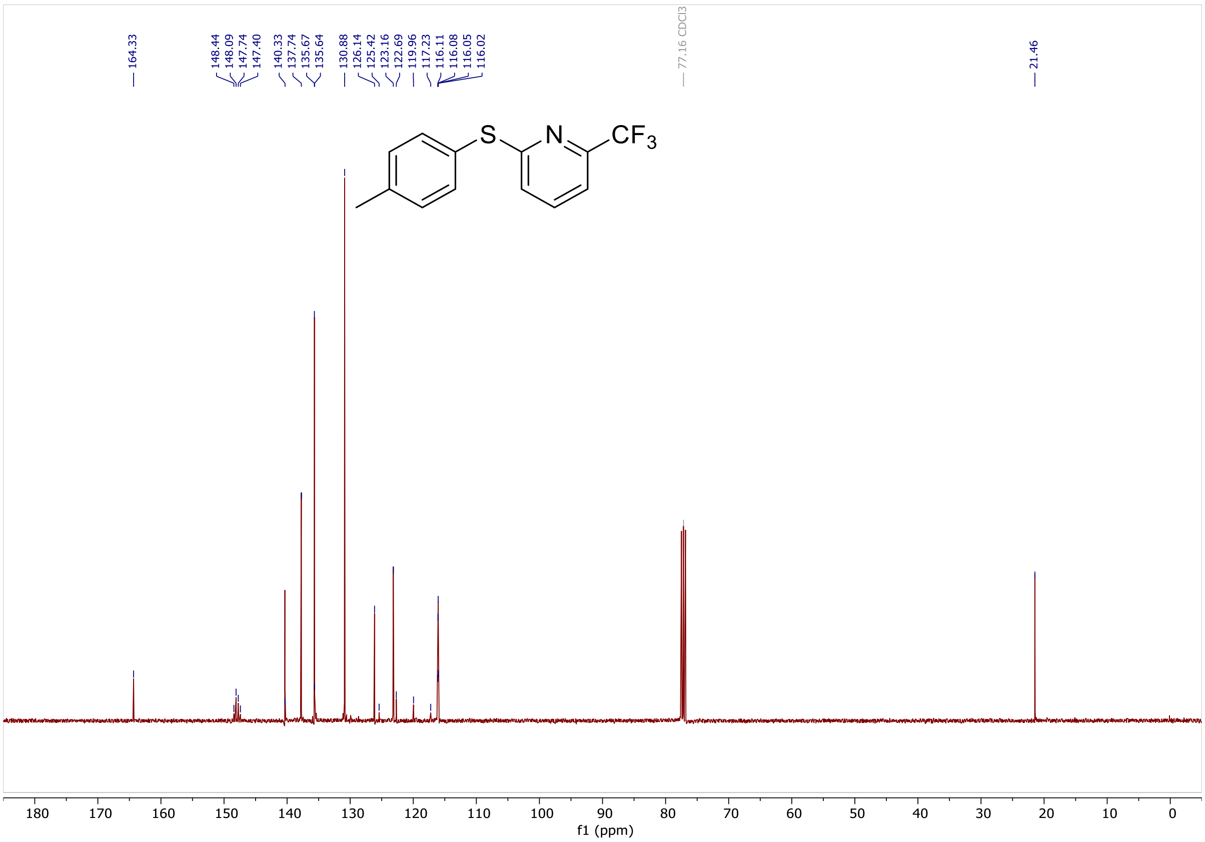

^{1}H NMR (400 MHz, $CDCl_3$): 1ji

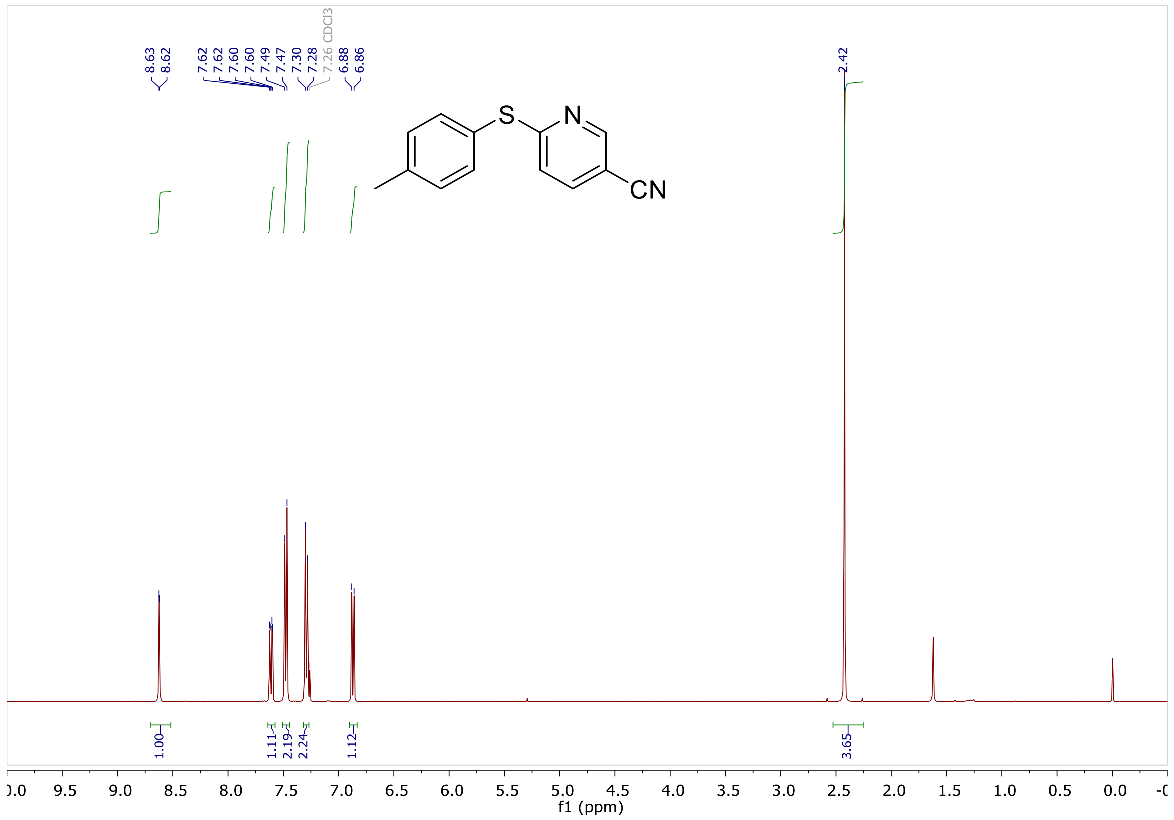

^{13}C NMR (101 MHz, $CDCl_3$): 1ji

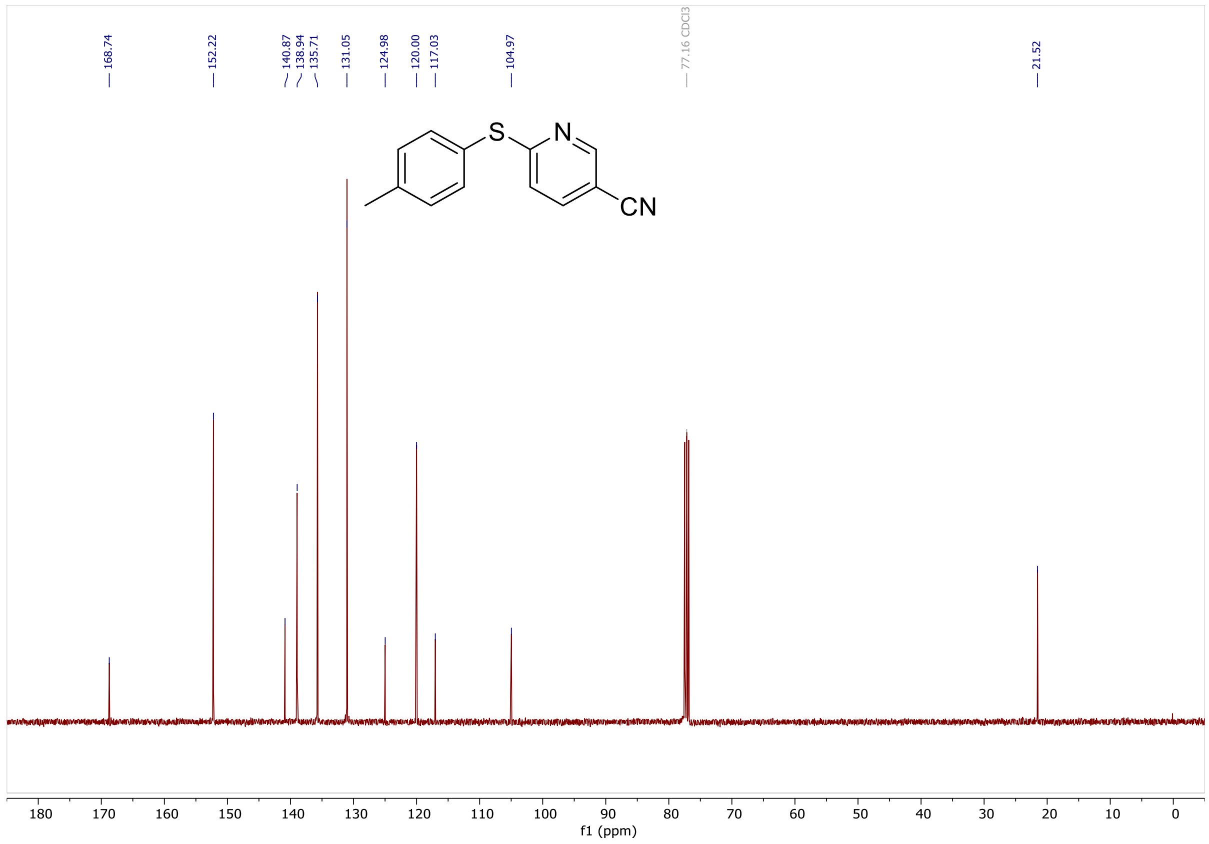

^{1}H NMR (400 MHz, $CDCl_3$): 1ki

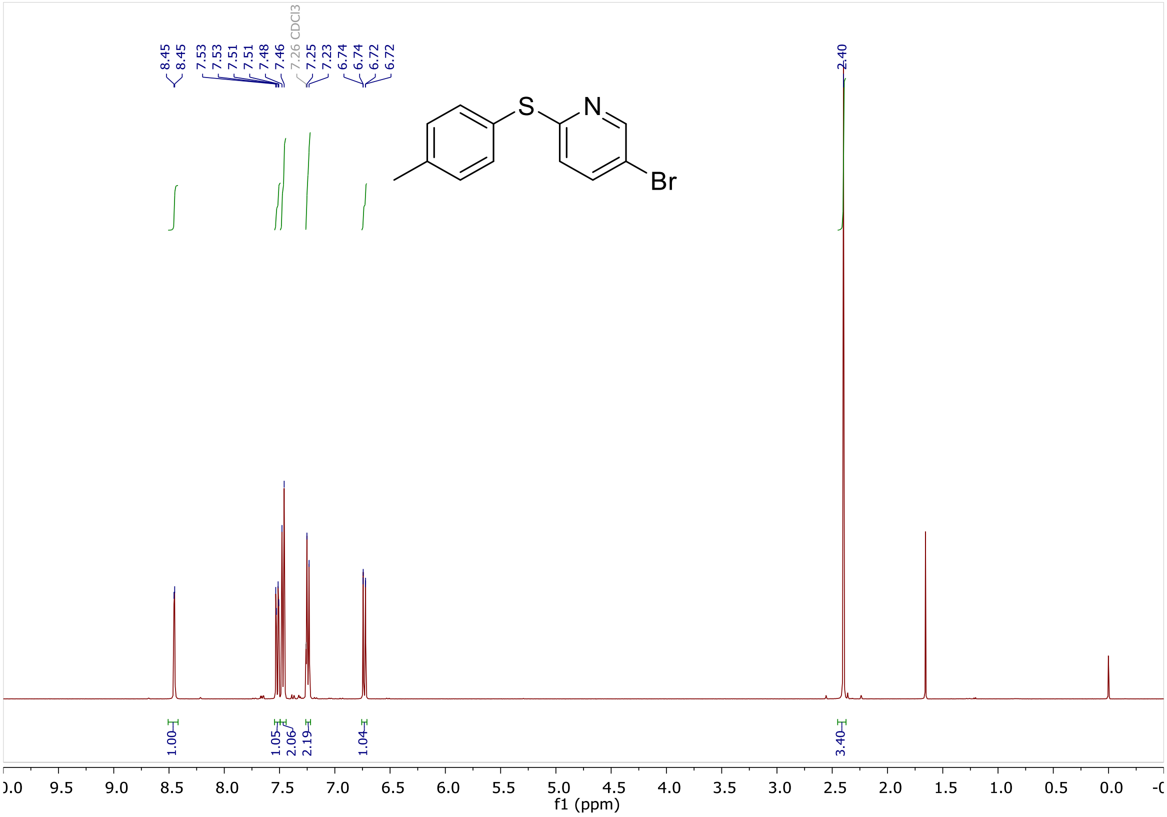

^{13}C NMR (101 MHz, $CDCl_3$): 1ki

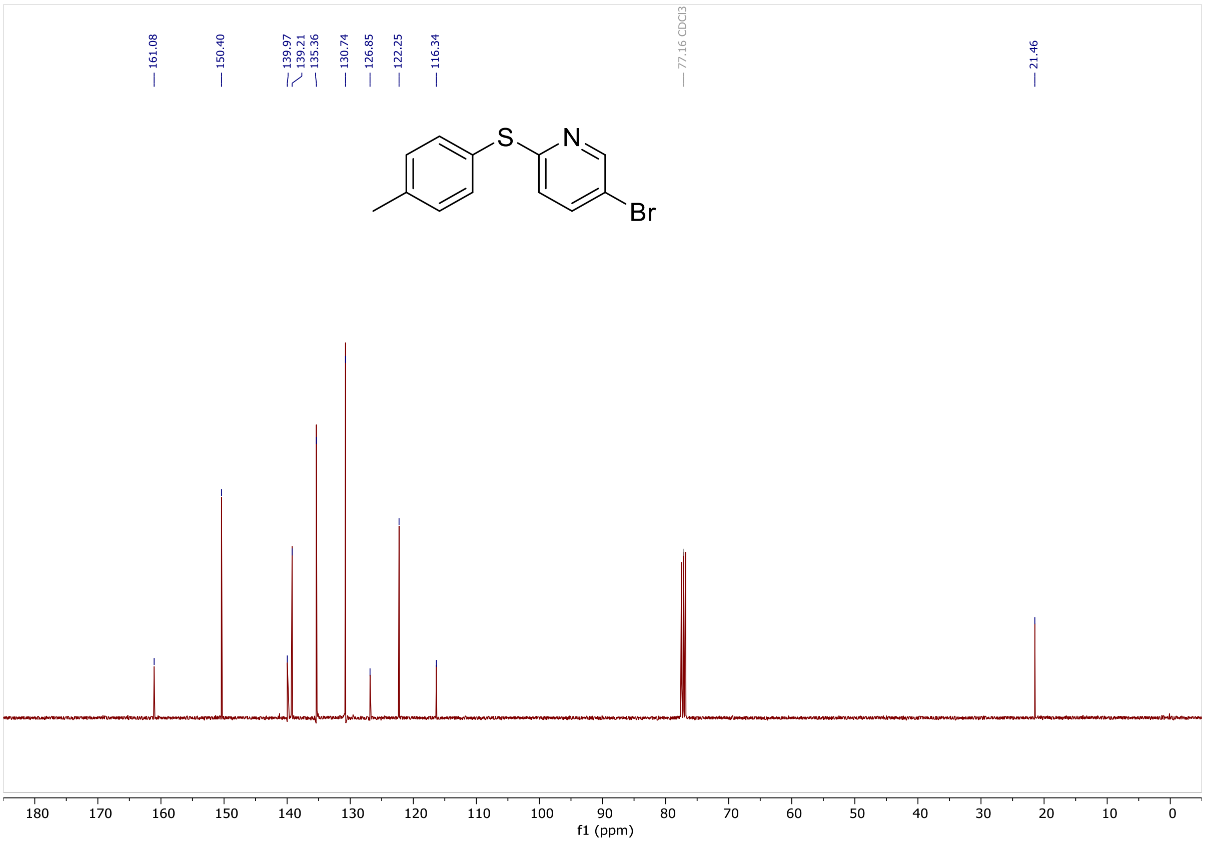

^{1}H NMR (400 MHz, $CDCl_3$): 1li

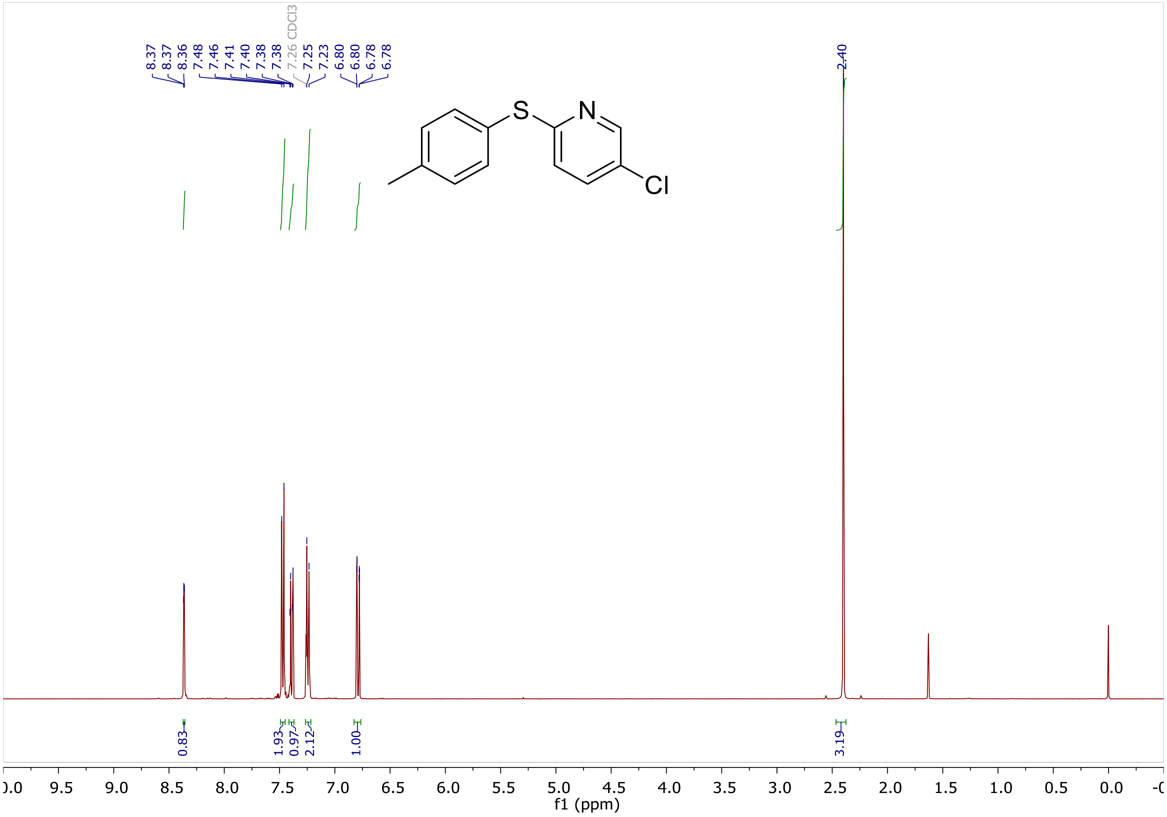

^{13}C NMR (101 MHz, $CDCl_3$): 1li

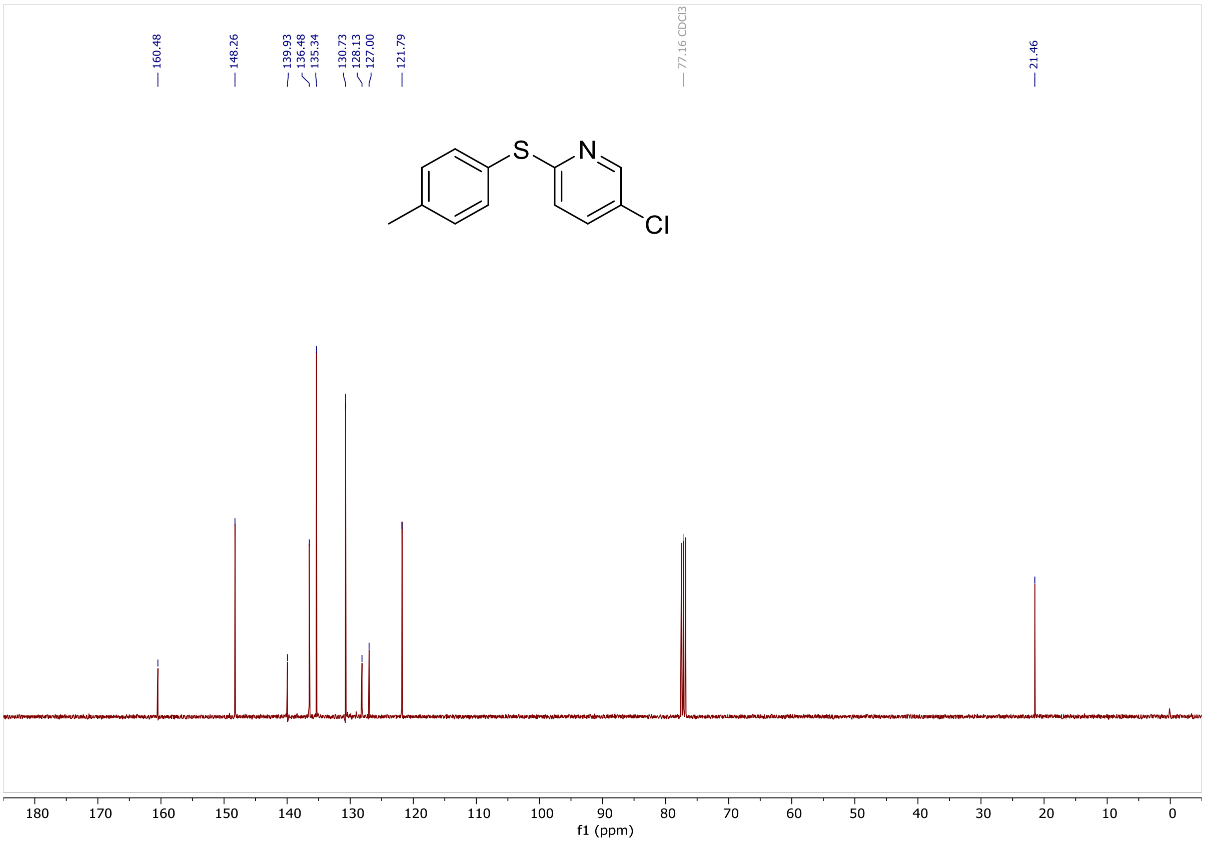

^{1}H NMR (400 MHz, CDCl$_3$): 1mi

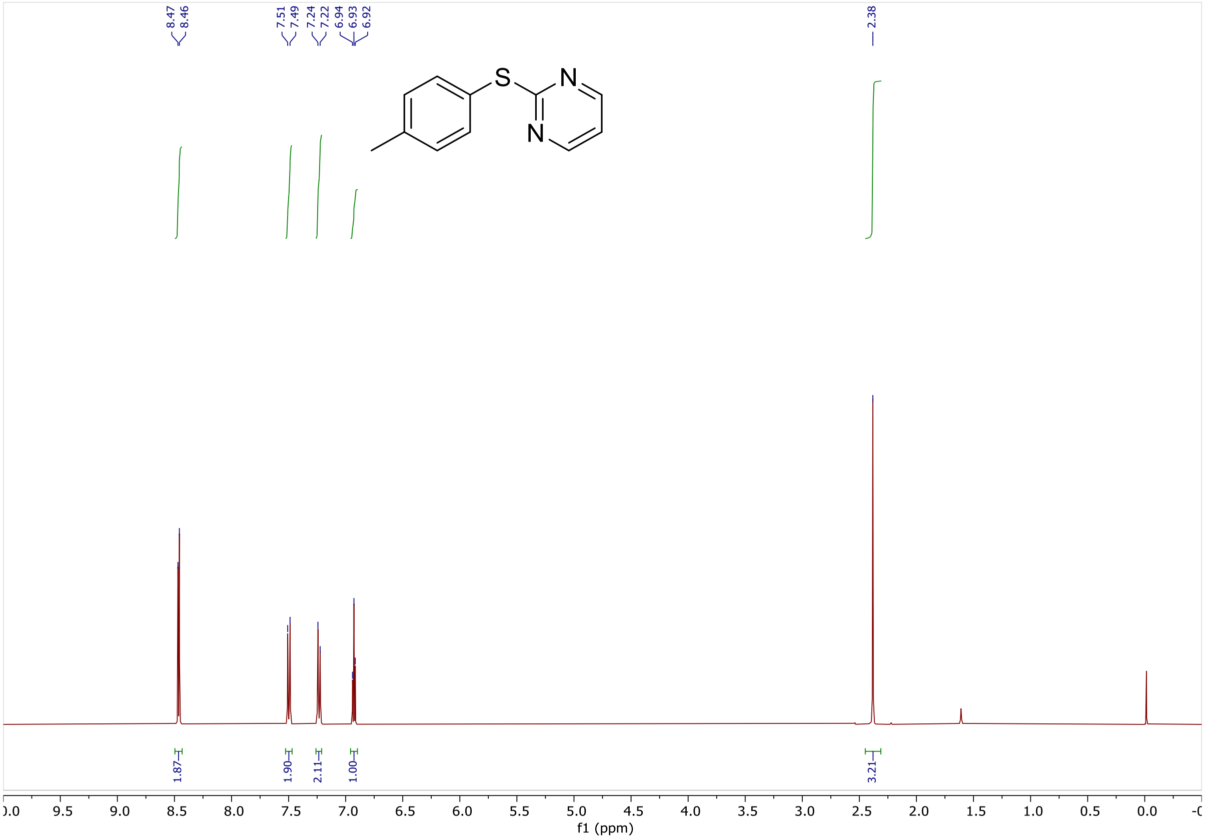

^{13}C NMR (101 MHz, CDCl$_3$): 1mi

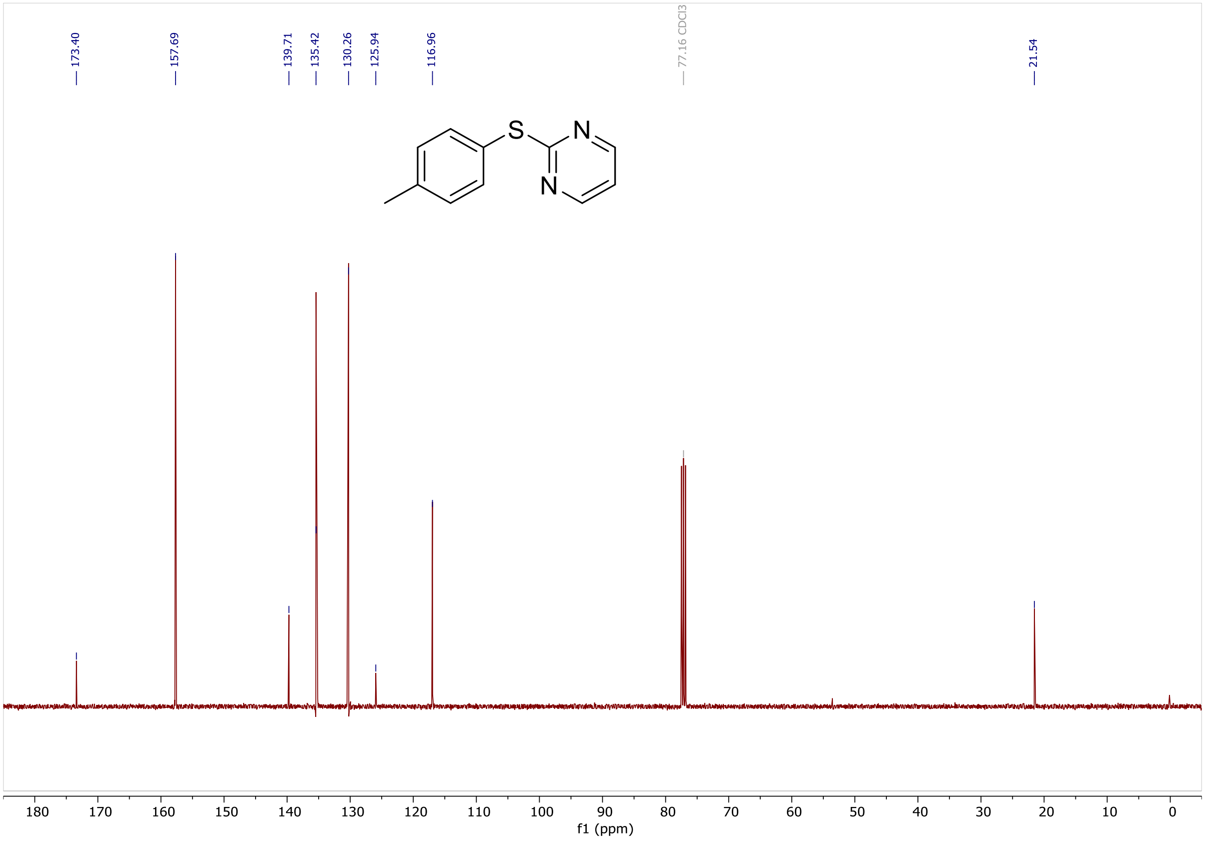

^{1}H NMR (500 MHz, $CDCl_3$): 1h

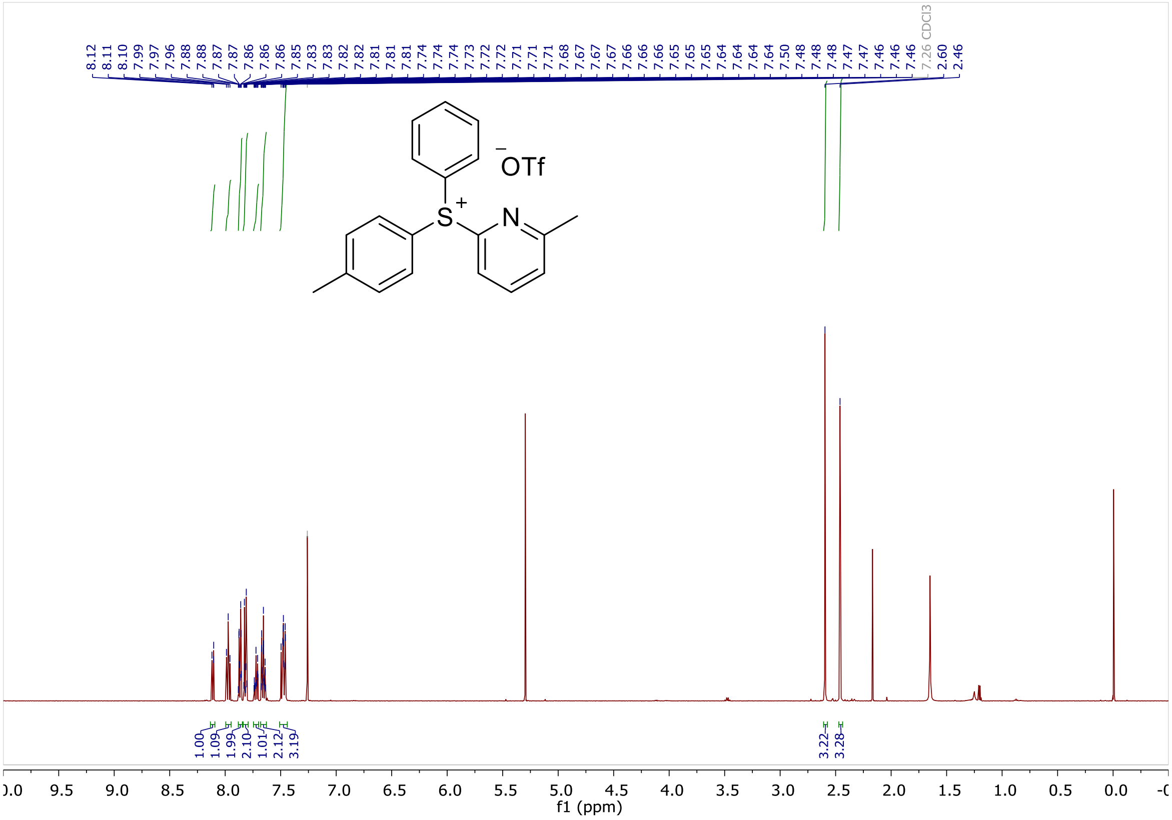

^{13}C NMR (126 MHz, $CDCl_3$): 1h

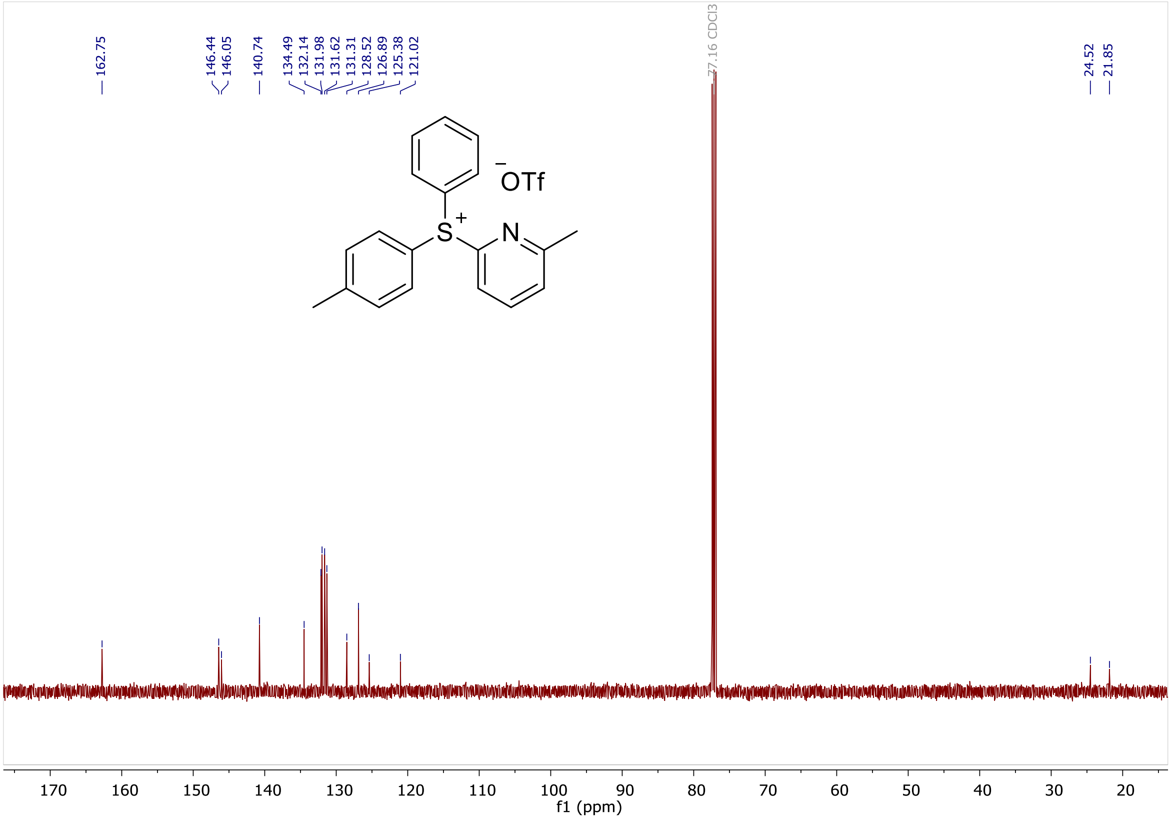

^{1}H NMR (400 MHz, $CDCl_3$): 1i

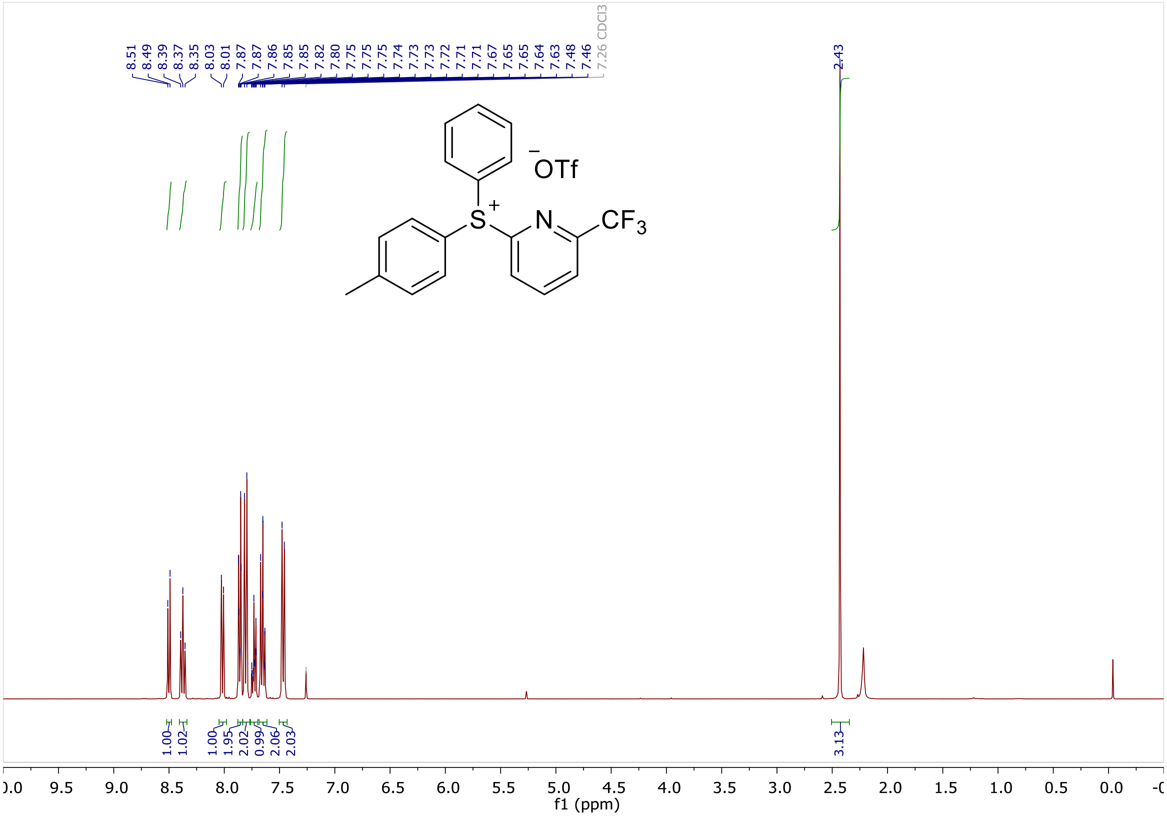

^{13}C NMR (101 MHz, $CDCl_3$): 1i

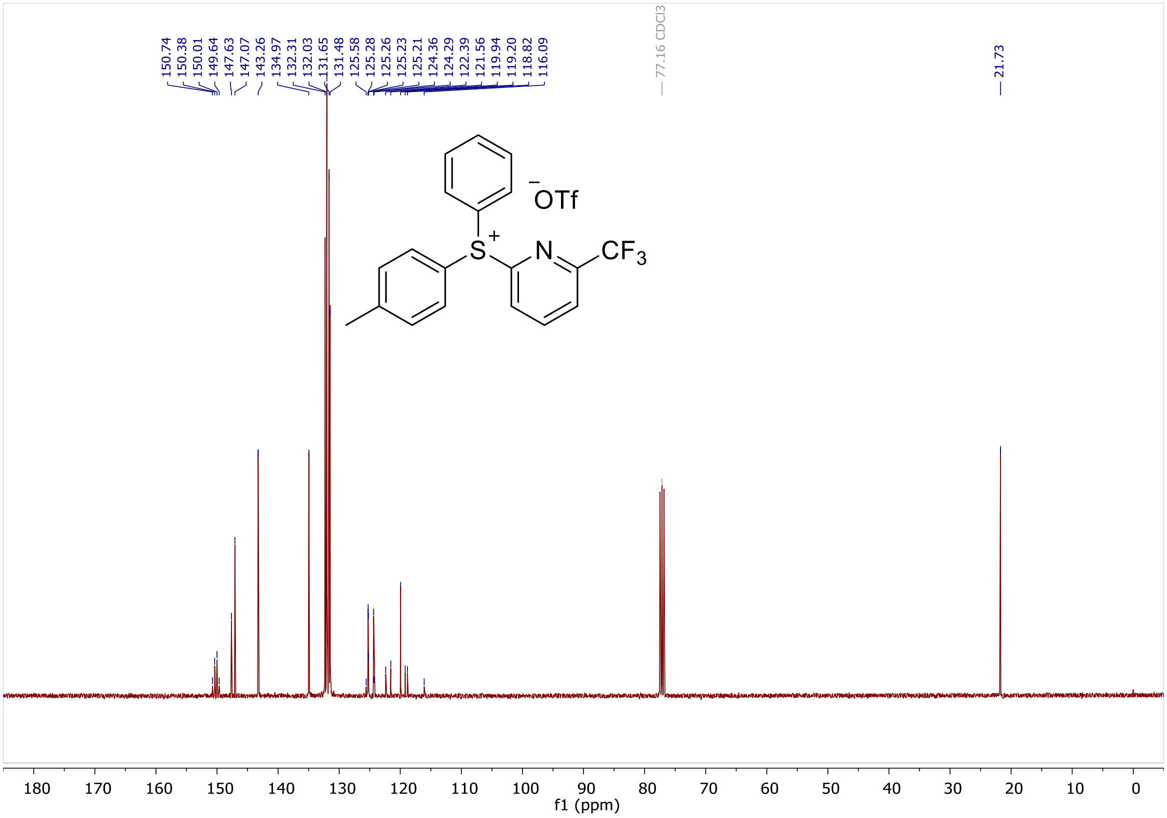

^{1}H NMR (500 MHz, $CDCl_3$): 1j

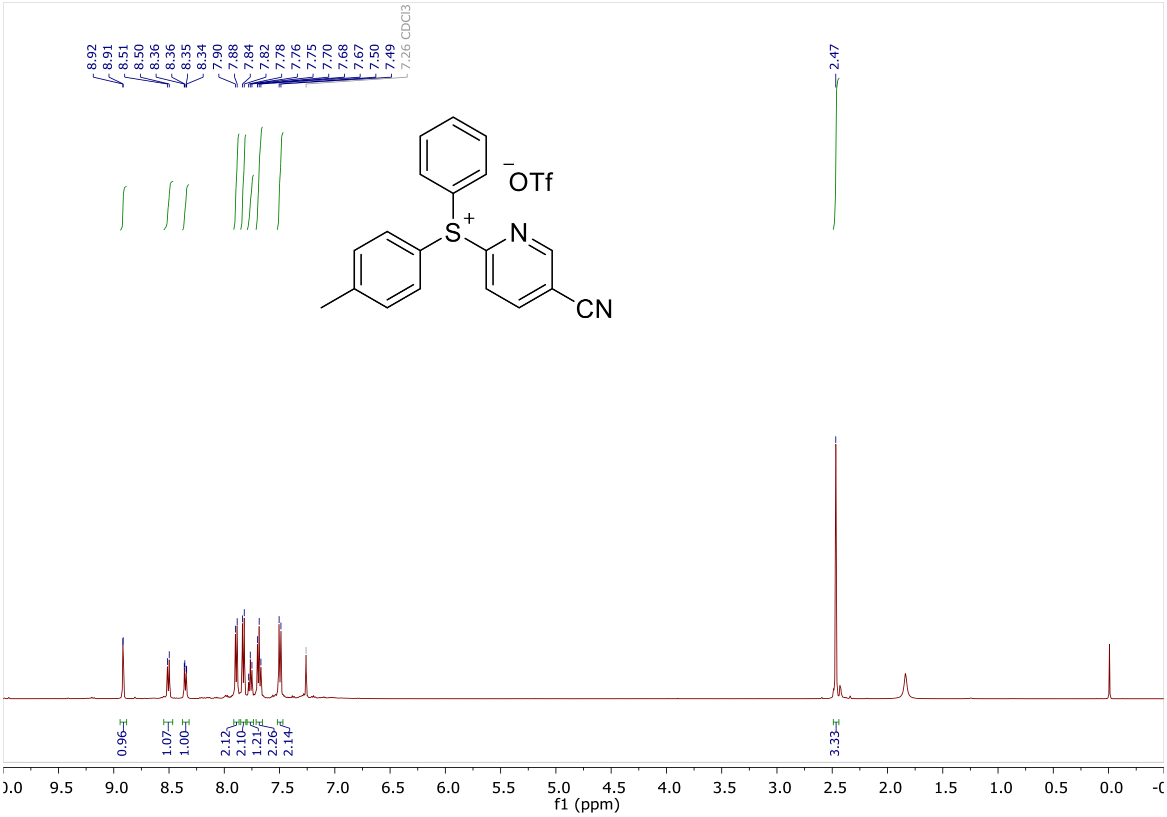

^{13}C NMR (126 MHz, $CDCl_3$): 1j

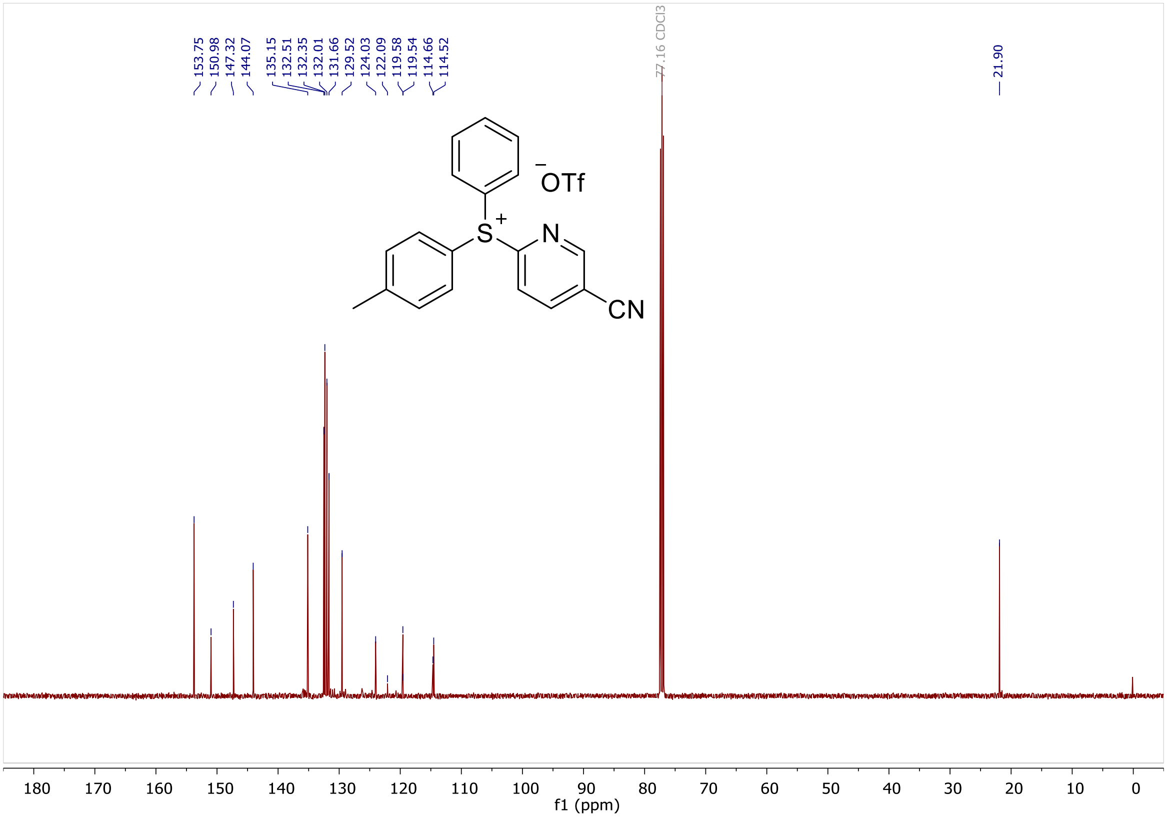

^{1}H NMR (500 MHz, CDCl$_3$): 1k

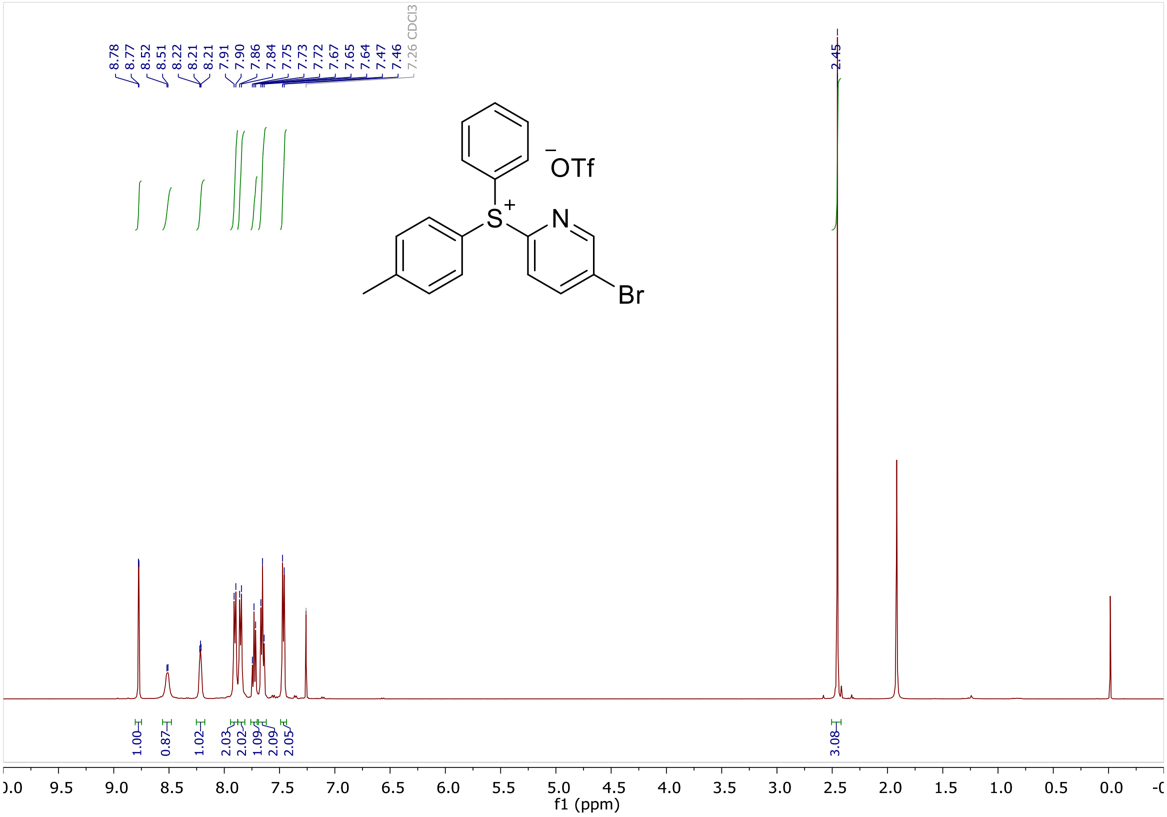

^{13}C NMR (126 MHz, CDCl$_3$): 1k

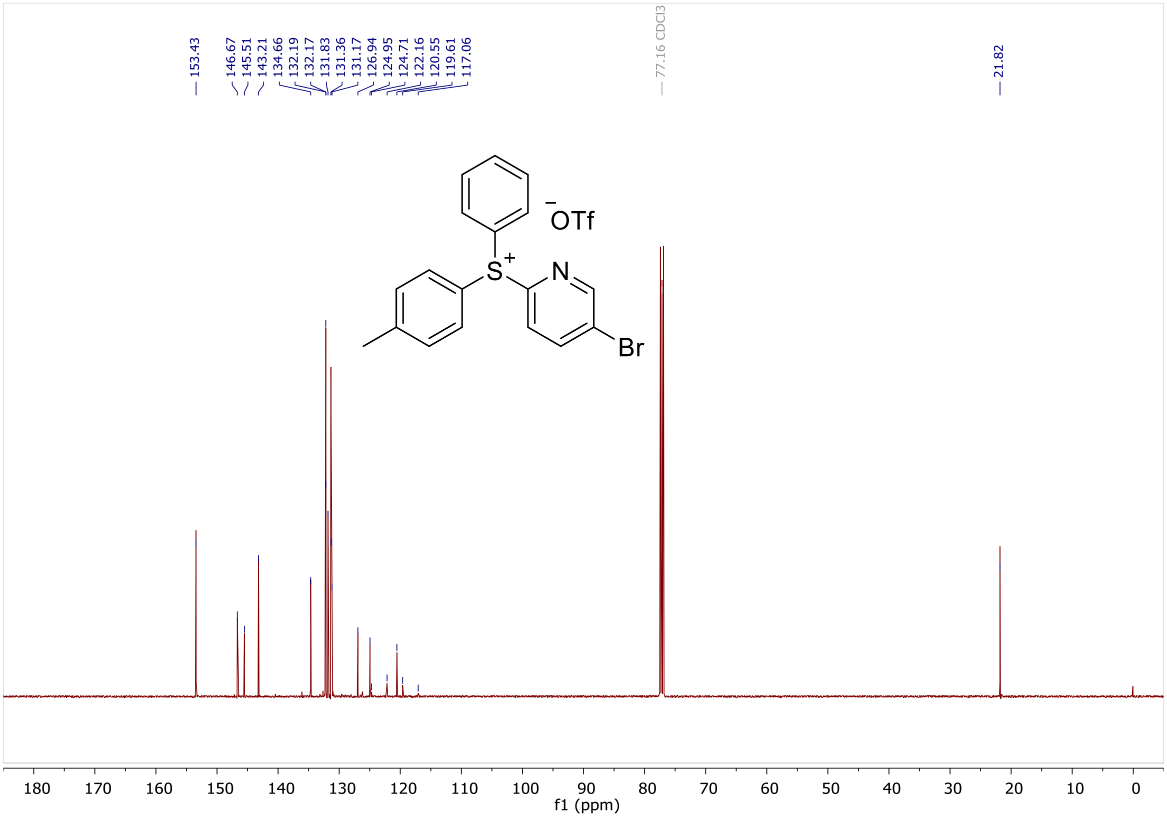

^{1}H NMR (500 MHz, $CDCl_3$): 1l

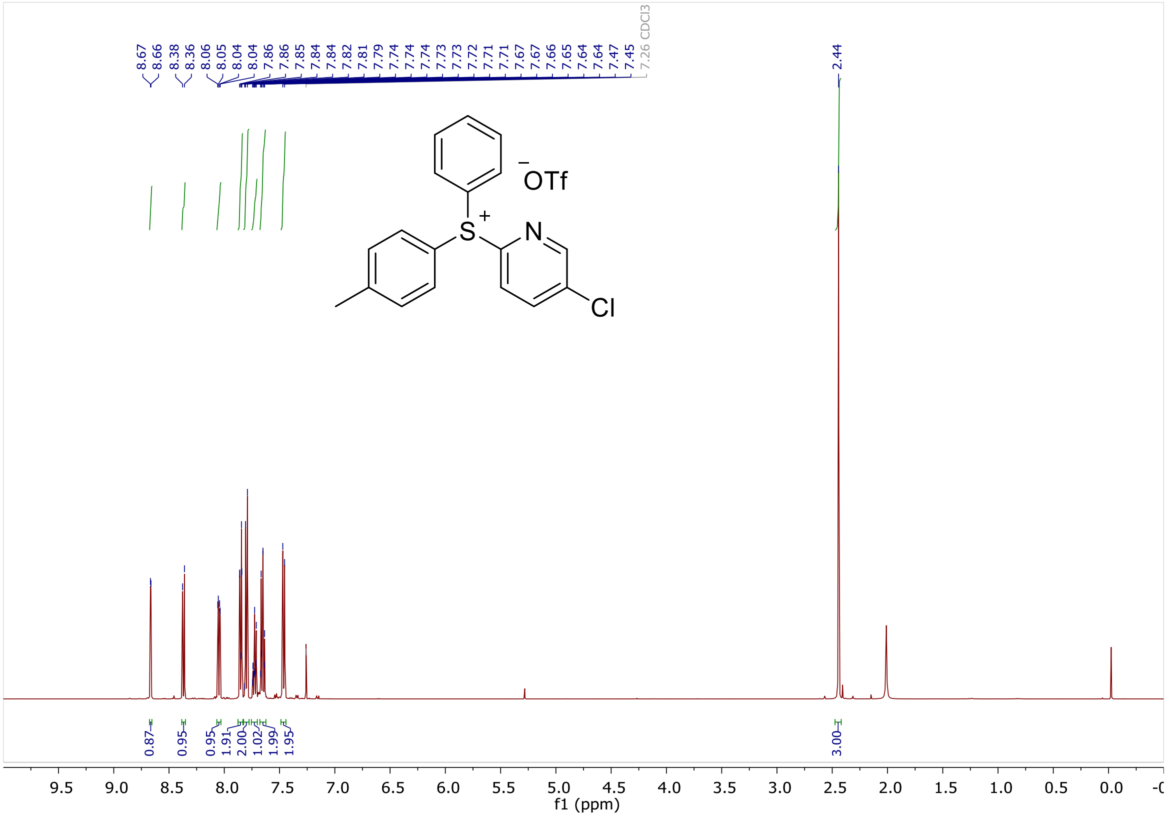

^{13}C NMR (101 MHz, $CDCl_3$): 1l

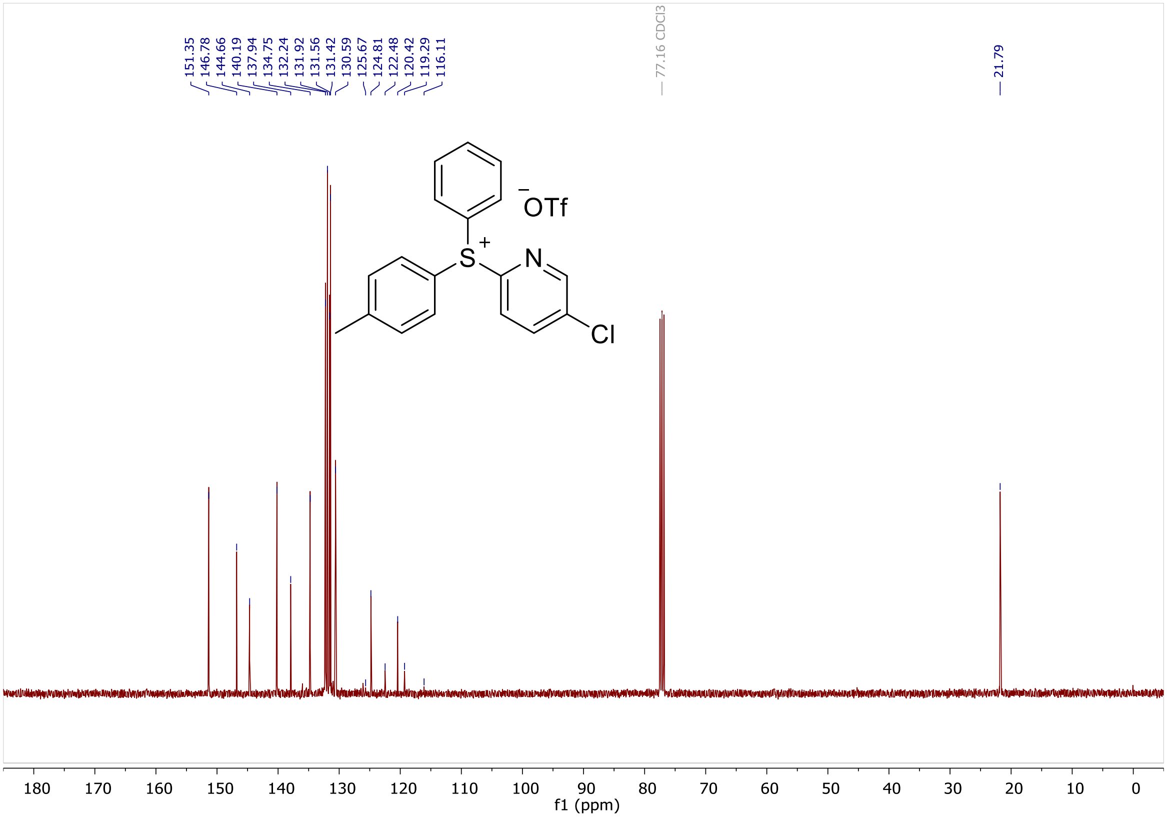

^{1}H NMR (500 MHz, $CDCl_3$): 1m

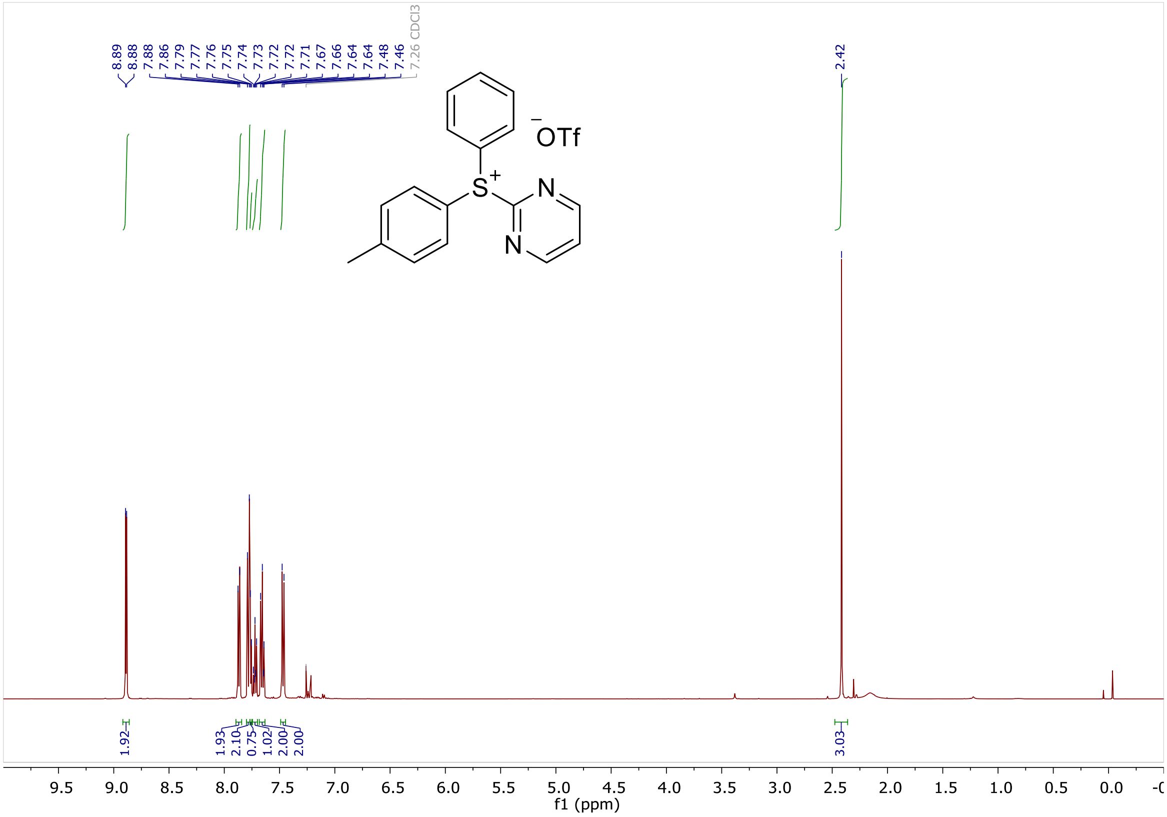

^{13}C NMR (126 MHz, $CDCl_3$): 1m

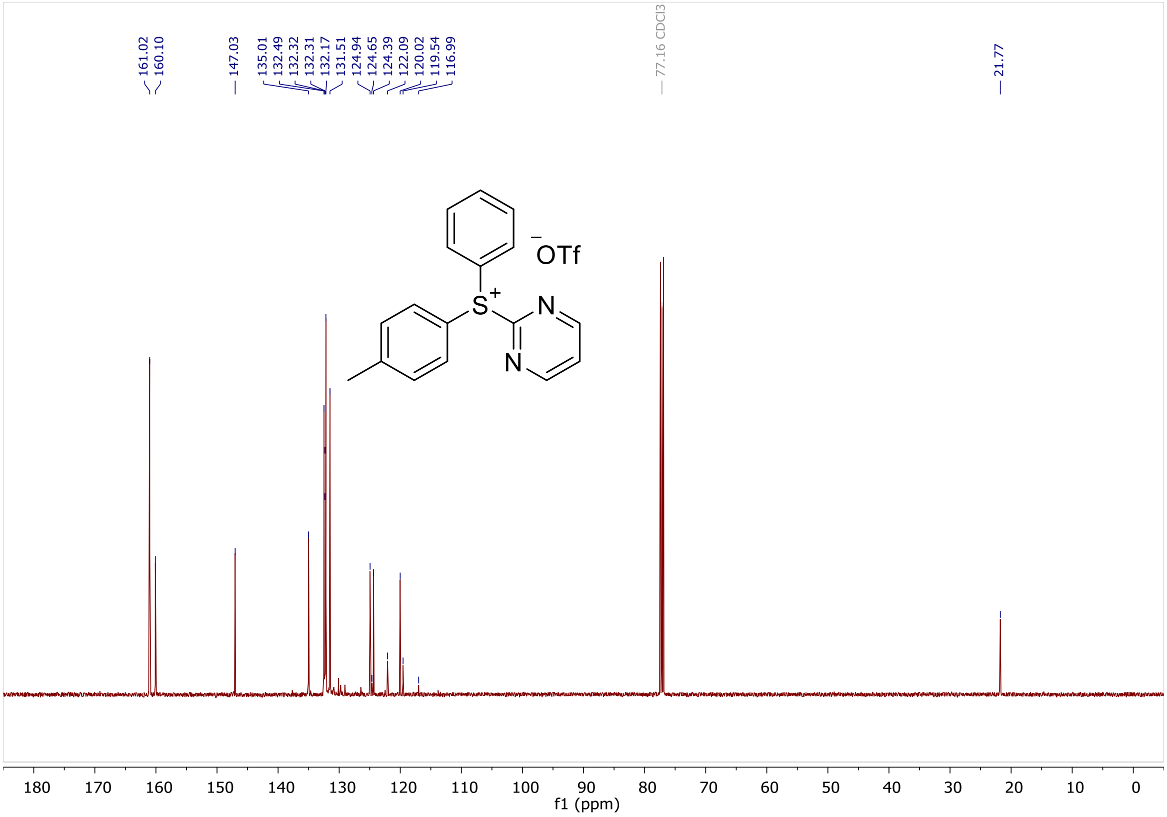

^{1}H NMR (400 MHz, $CDCl_3$): 2

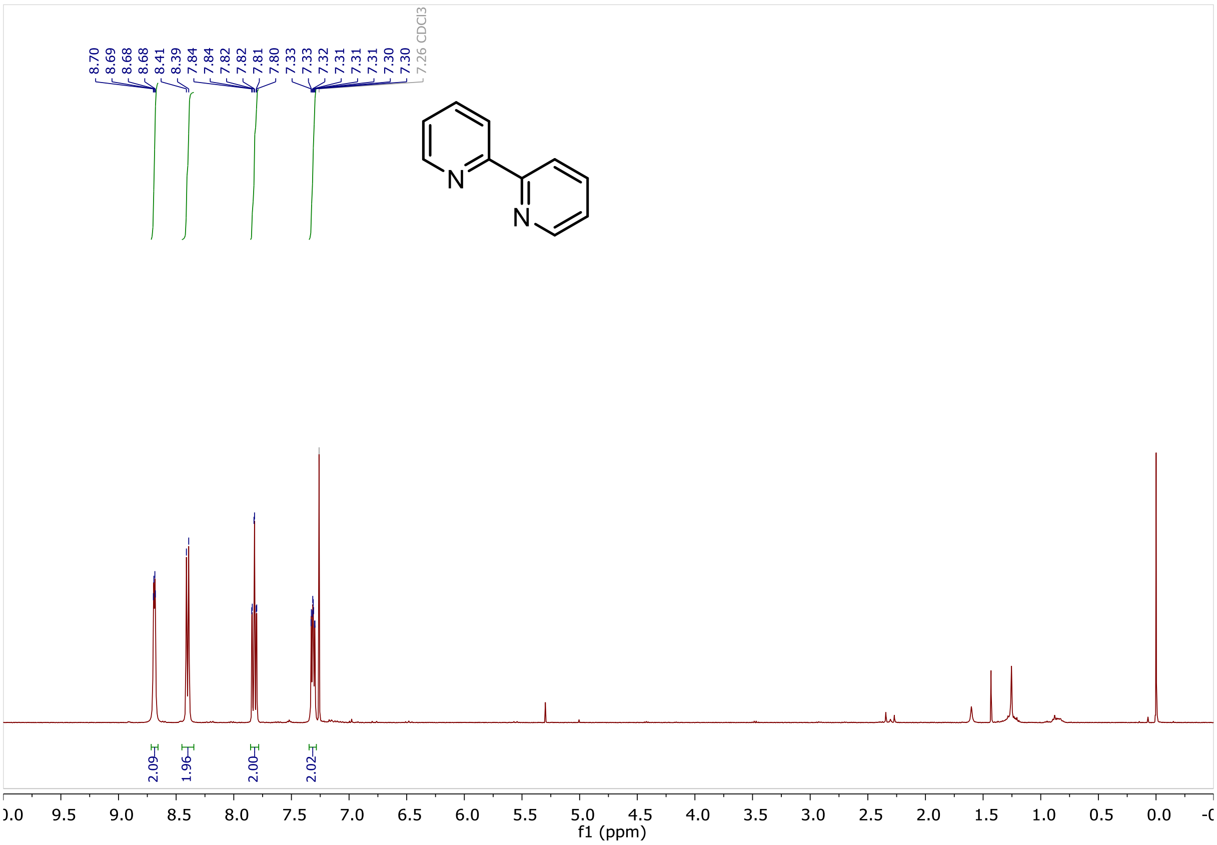

^{13}C NMR (101 MHz, $CDCl_3$): 2

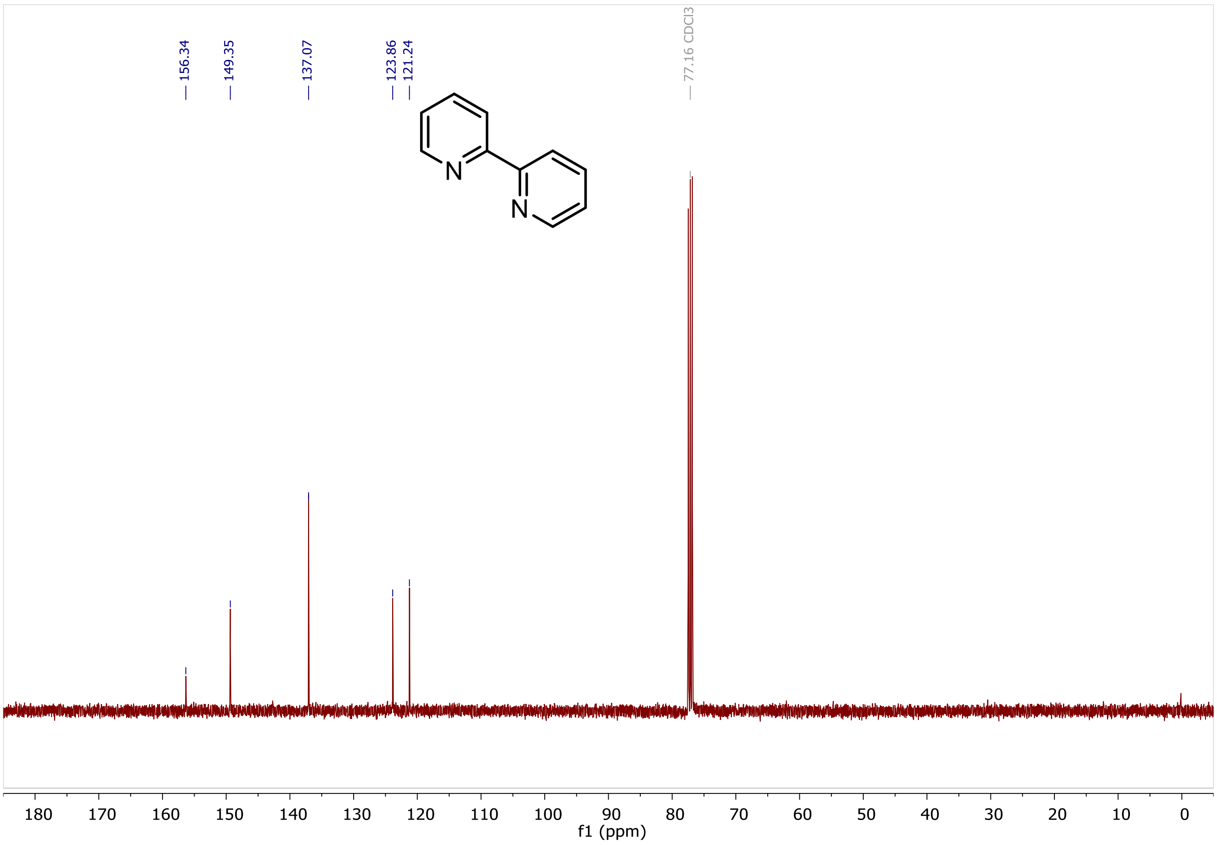

^{1}H NMR (400 MHz, $CDCl_3$): 3

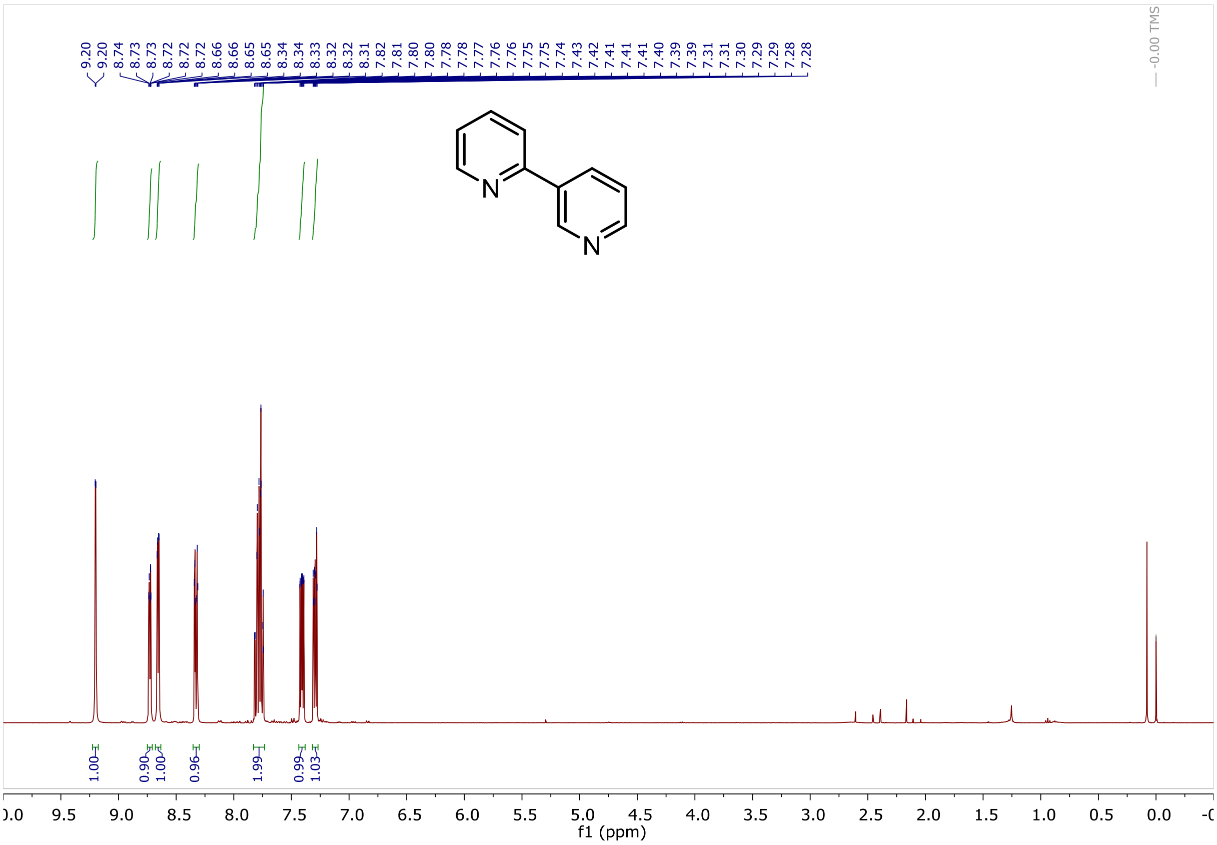

^{13}C NMR (101 MHz, $CDCl_3$): 3

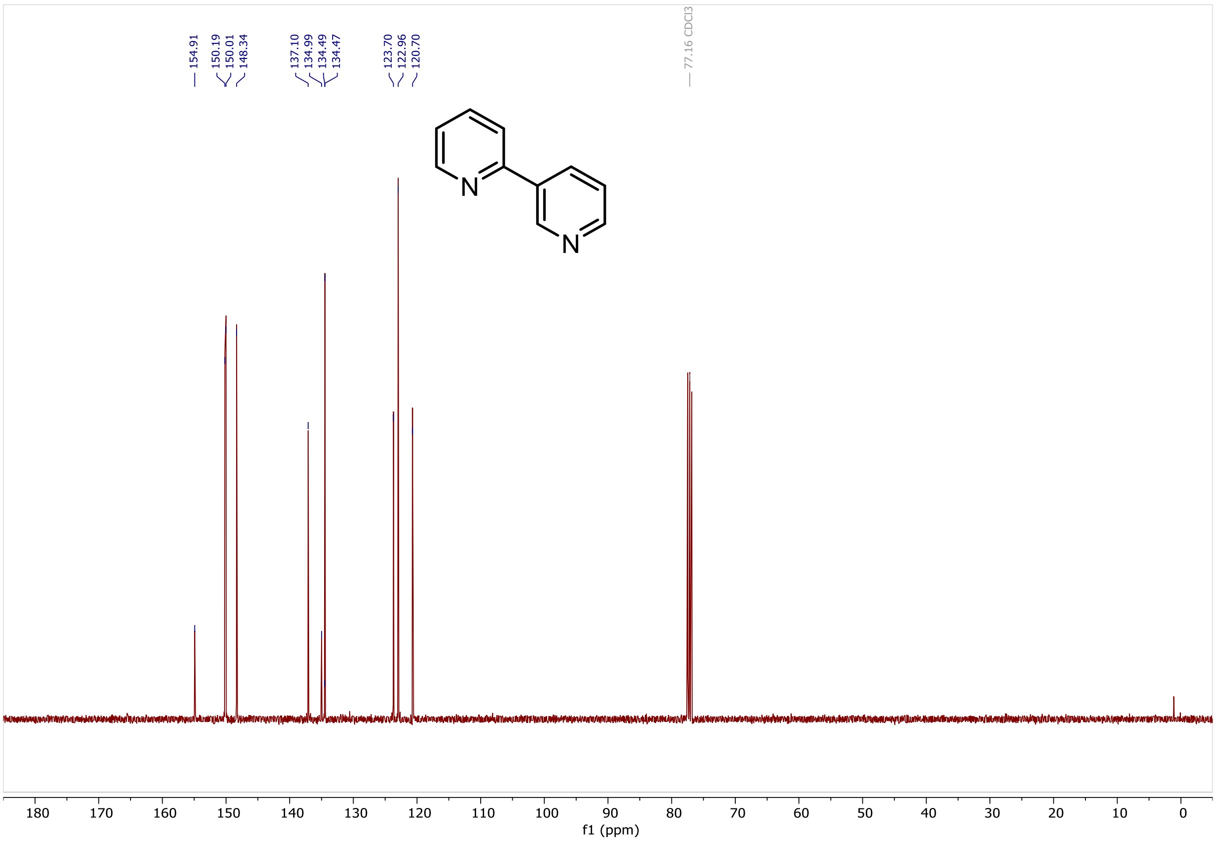

^{1}H NMR (500 MHz, CDCl$_3$): 4

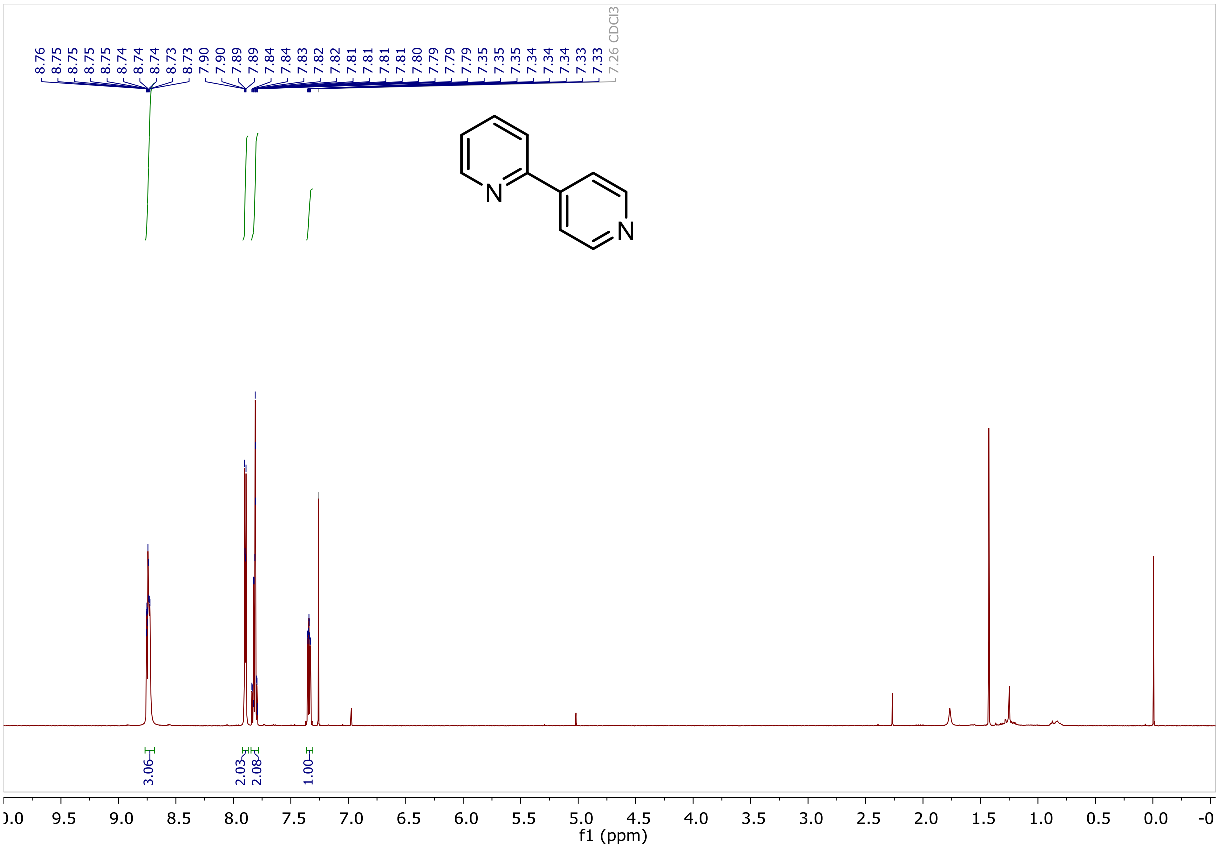

^{13}C NMR (126 MHz, CDCl$_3$): 4

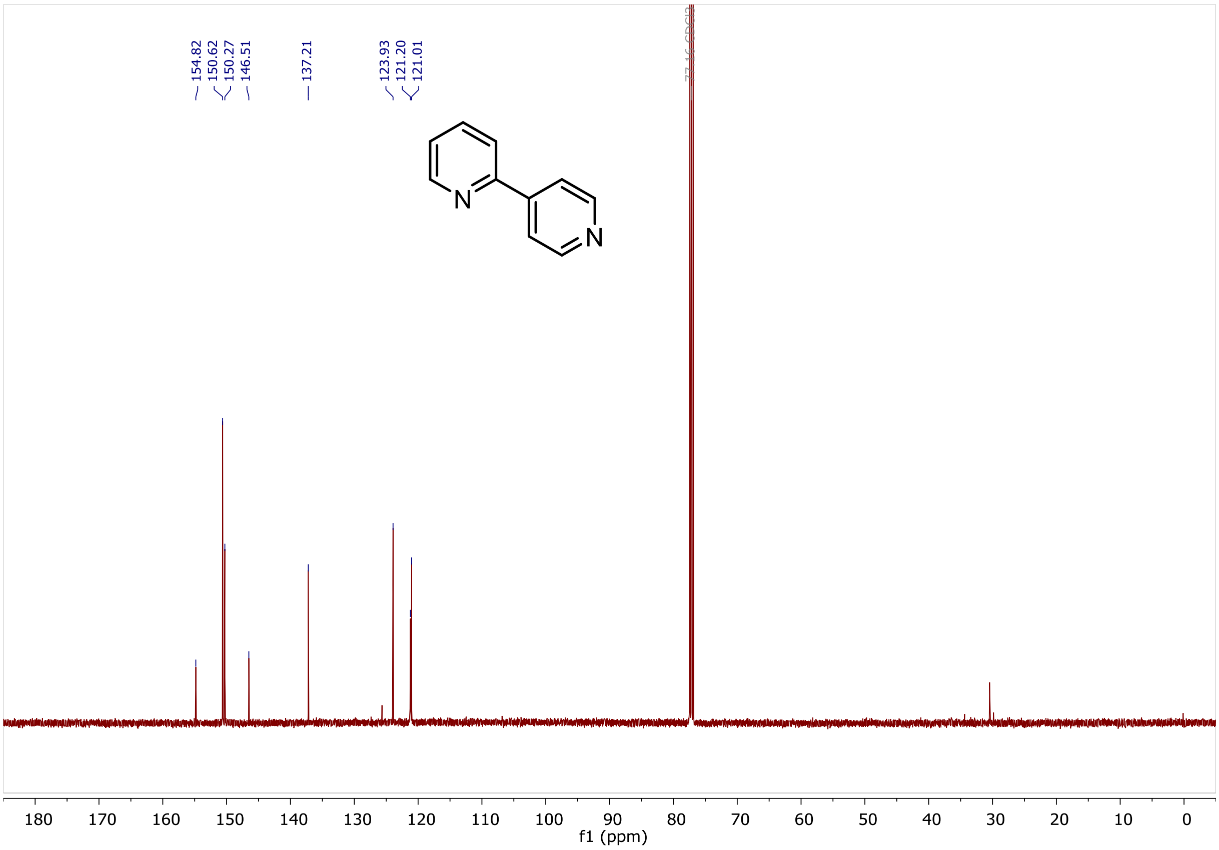

^{1}H NMR (500 MHz, $CDCl_3$): 5

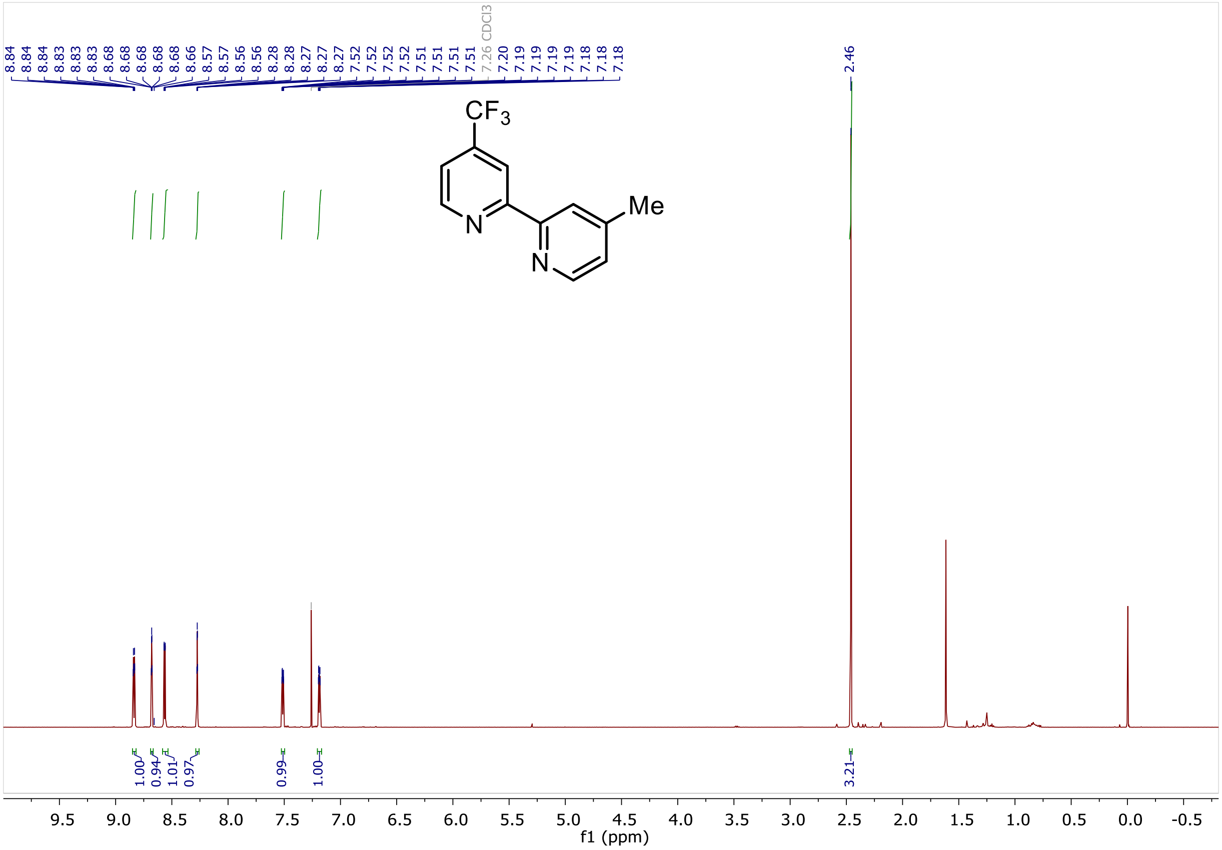

^{13}C NMR (126 MHz, $CDCl_3$): 5

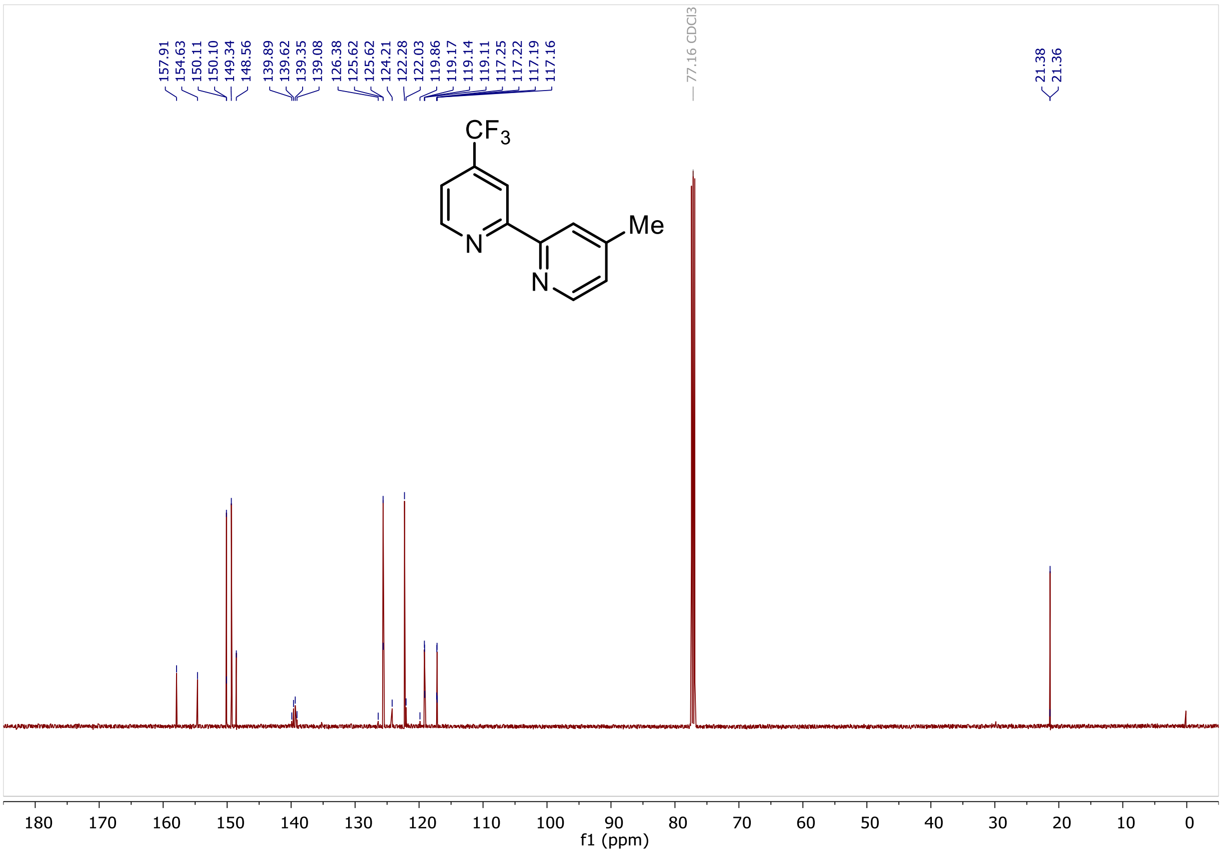

^{1}H NMR (500 MHz, $CDCl_3$): 6

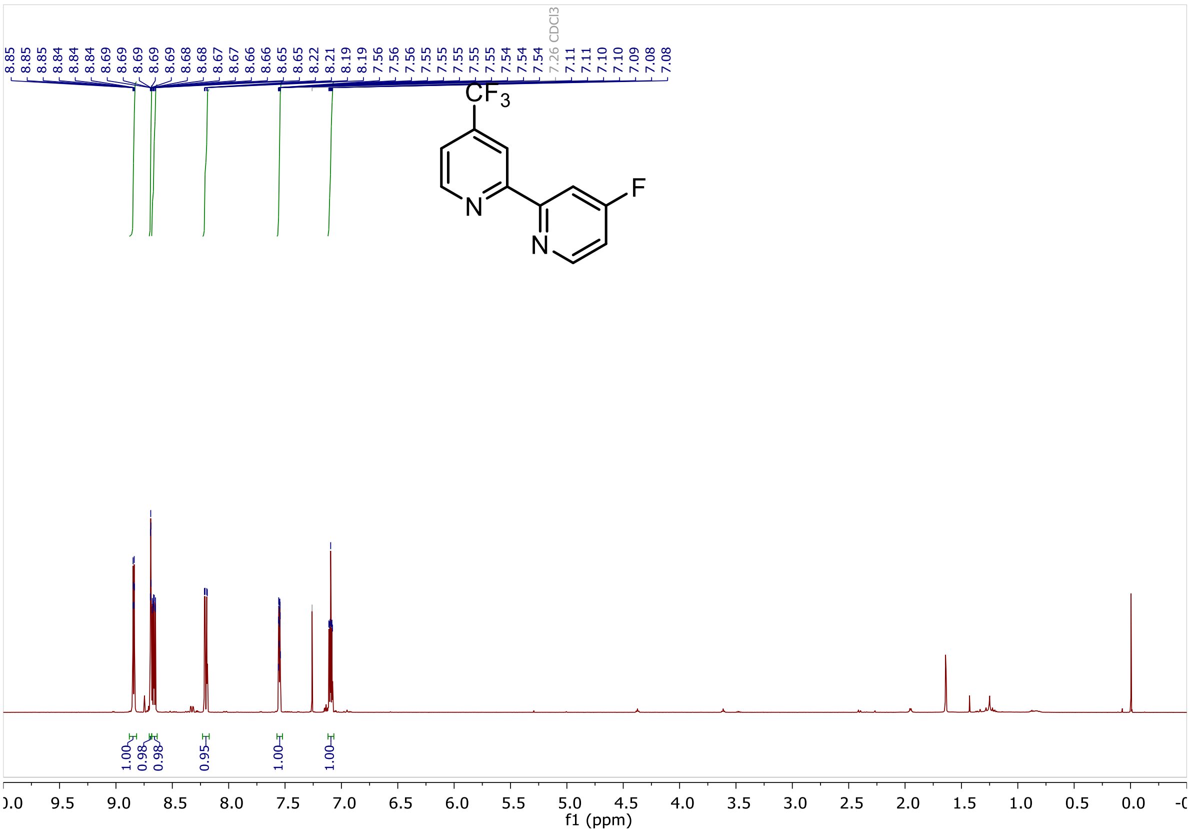

^{13}C NMR (126 MHz, $CDCl_3$): 6

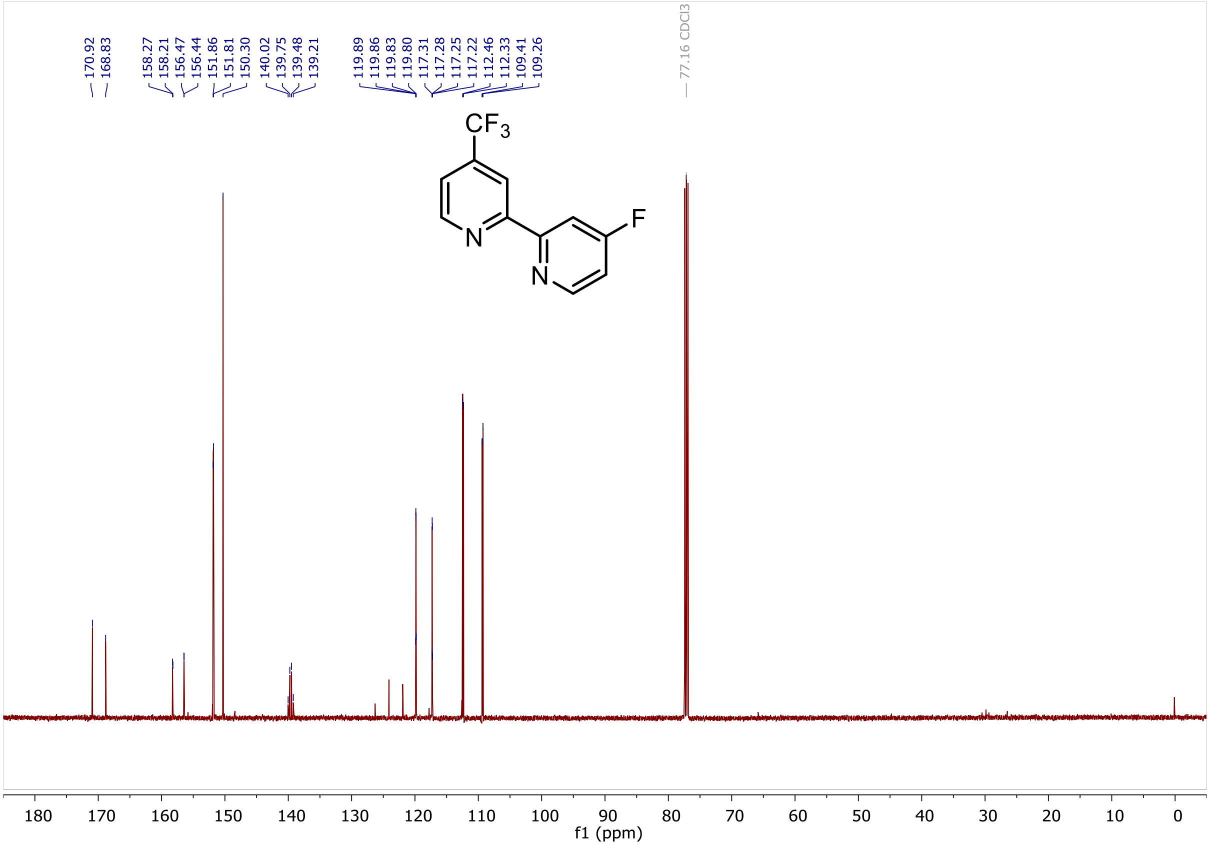

^{1}H NMR (500 MHz, $CDCl_3$): 7

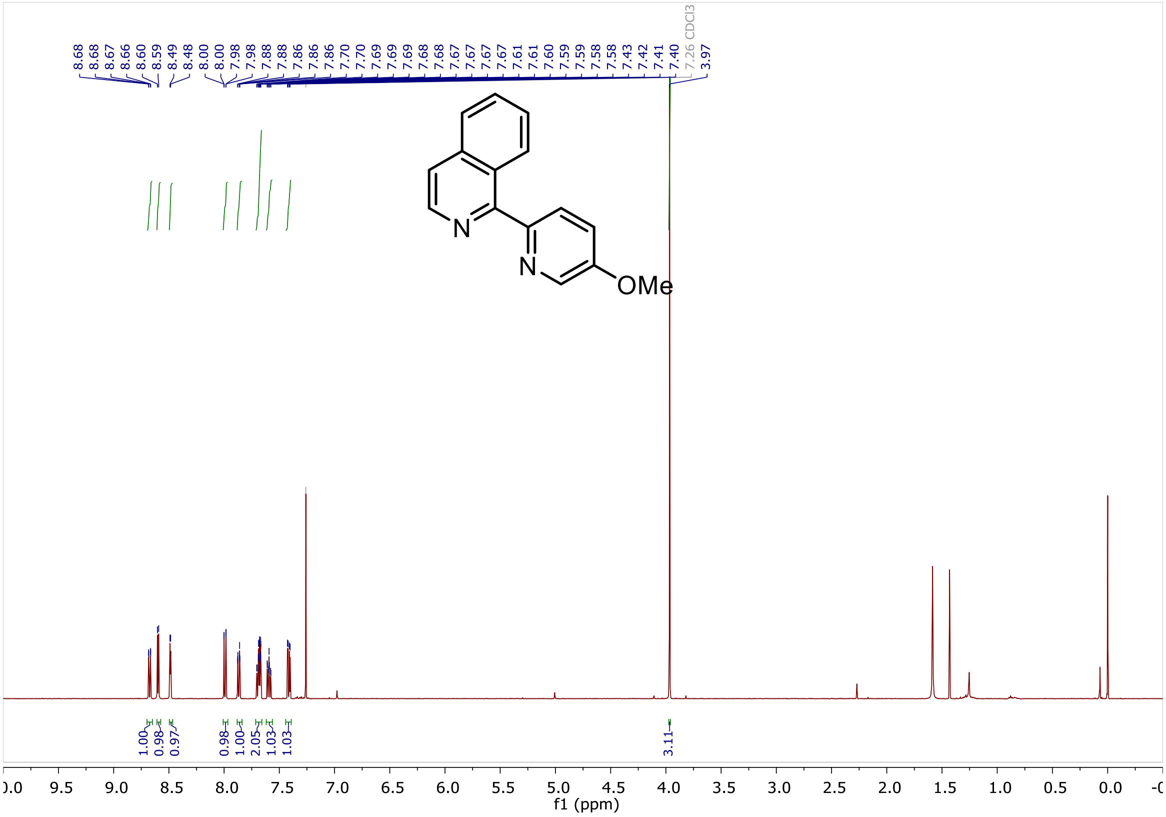

^{13}C NMR (126 MHz, $CDCl_3$): 7

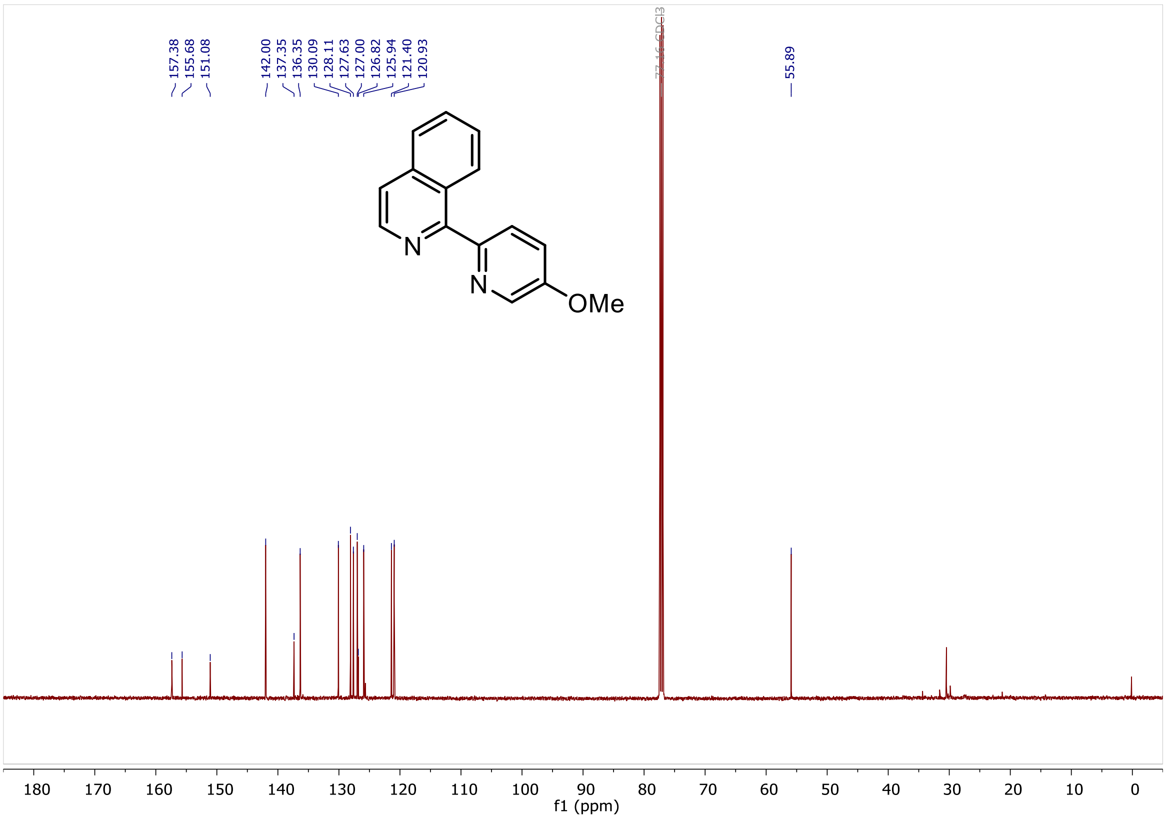

^{1}H NMR (500 MHz, CDCl$_3$): 8

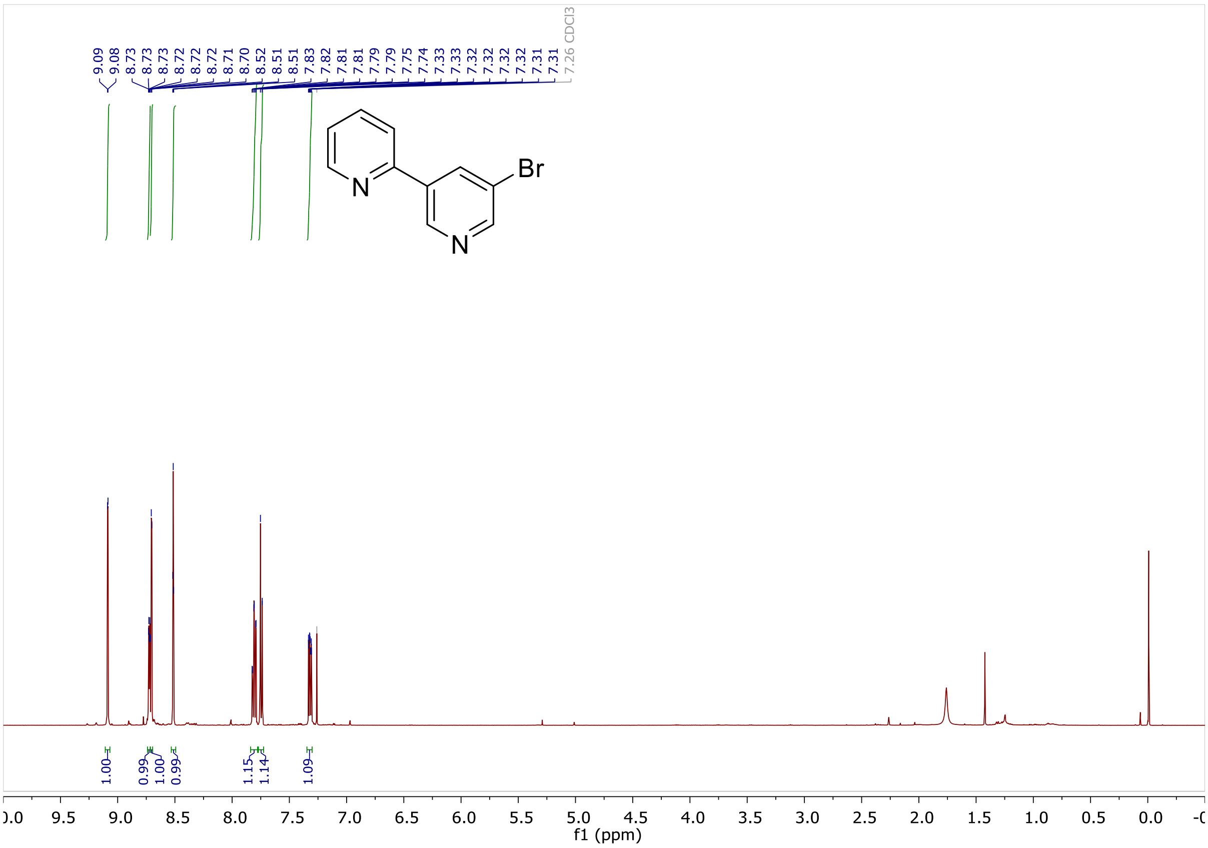

^{13}C NMR (126 MHz, CDCl$_3$): 8

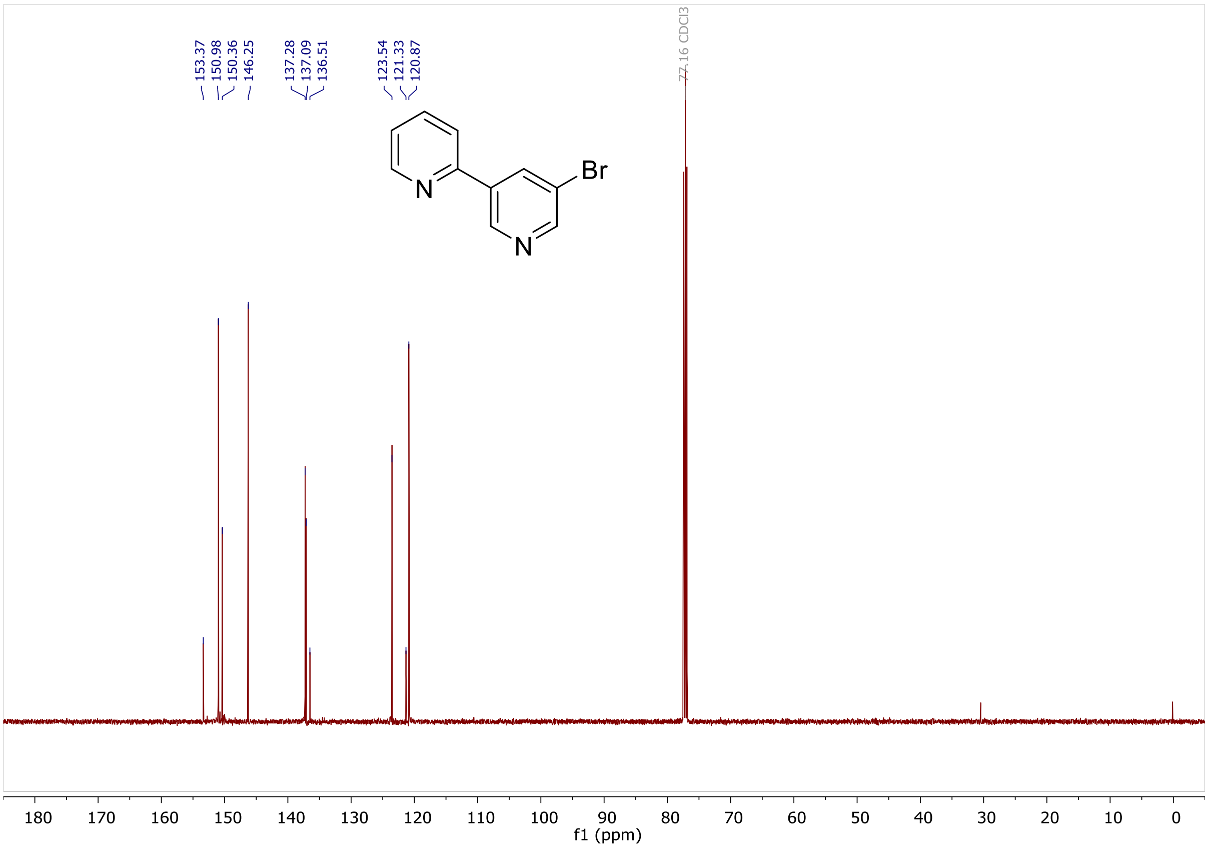

^{1}H NMR (500 MHz, $CDCl_3$): 9

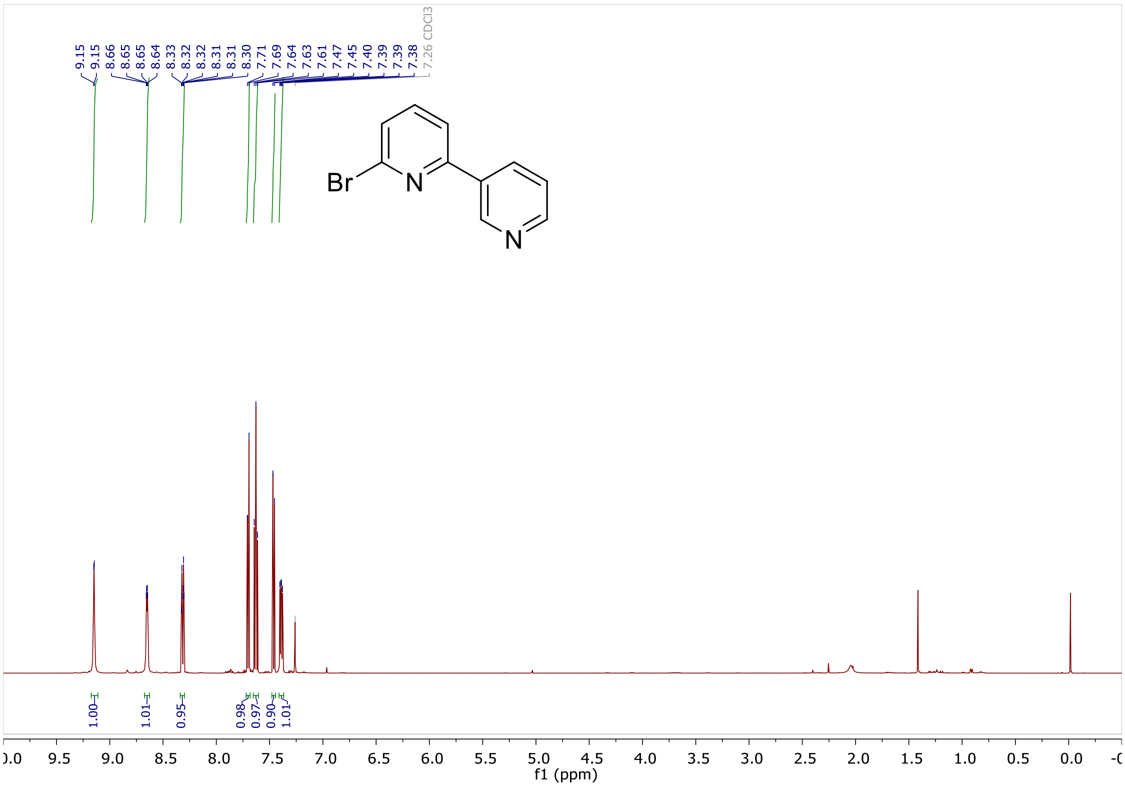

^{13}C NMR (126 MHz, $CDCl_3$): 9

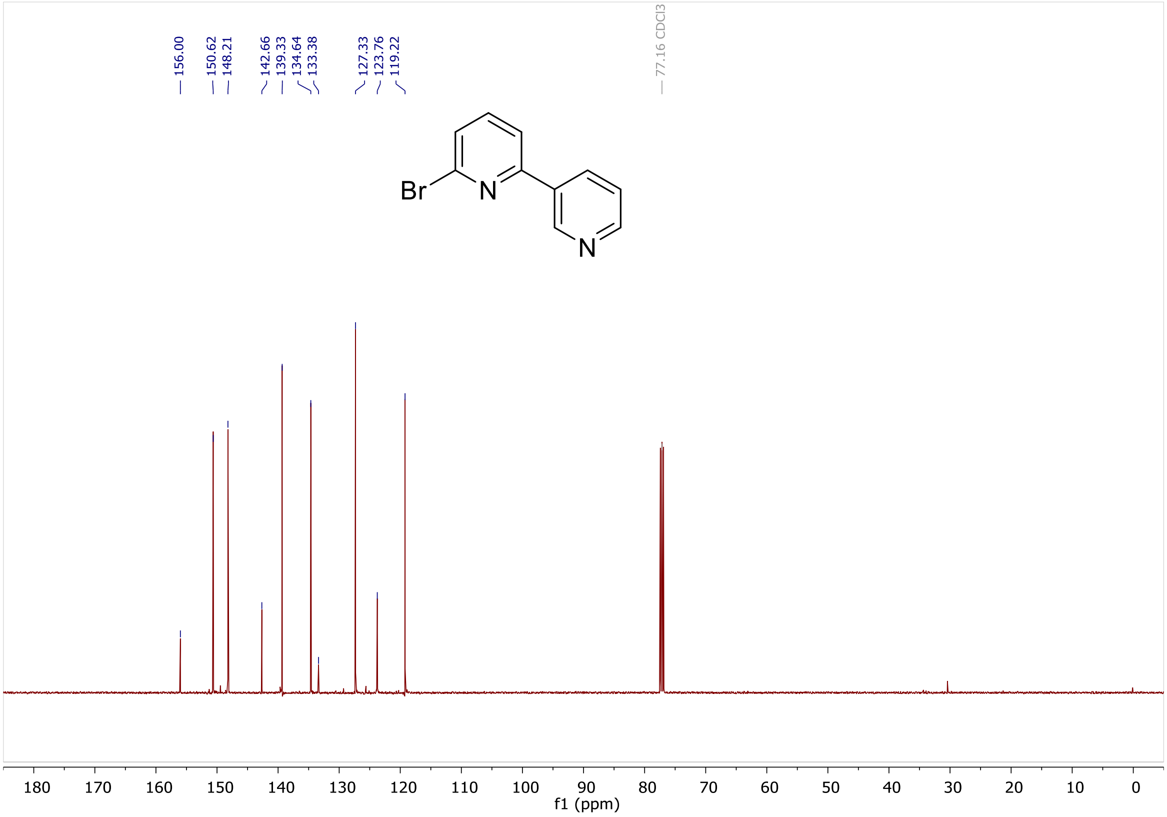

^{1}H NMR (400 MHz, $CDCl_3$): 10

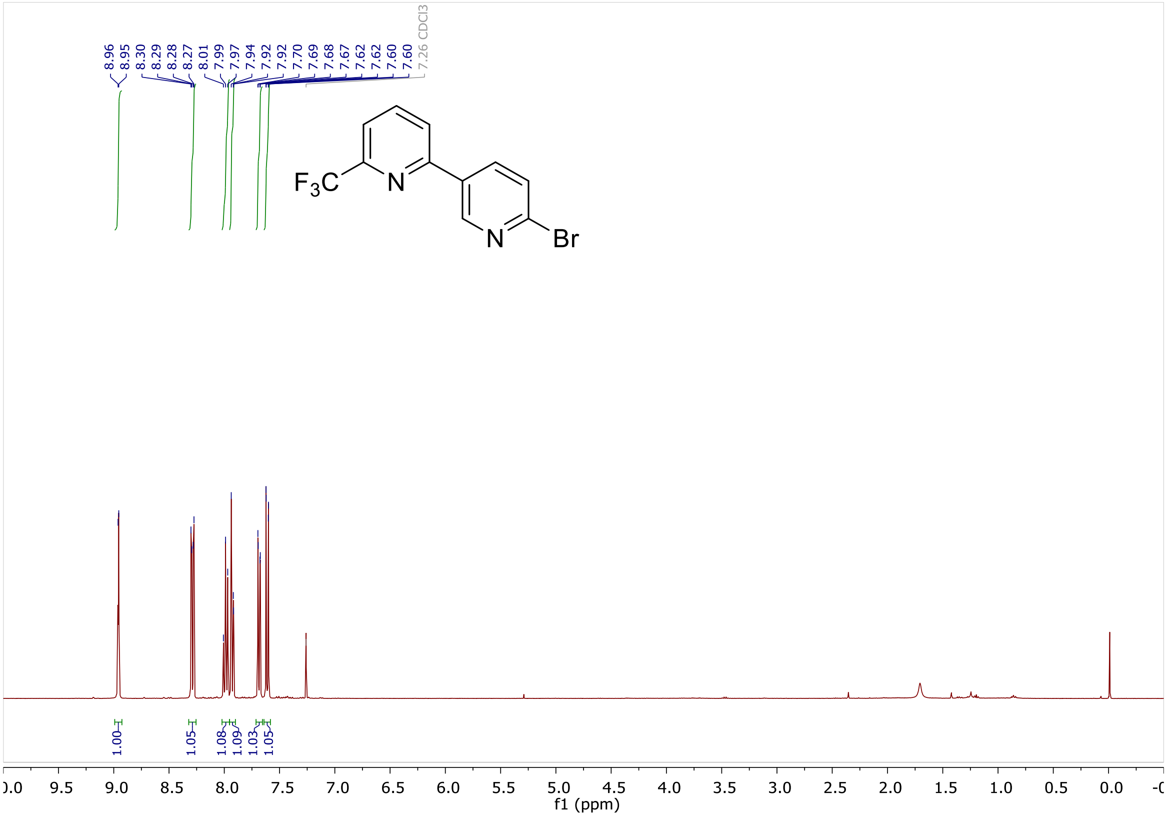

^{13}C NMR (101 MHz, $CDCl_3$): 10

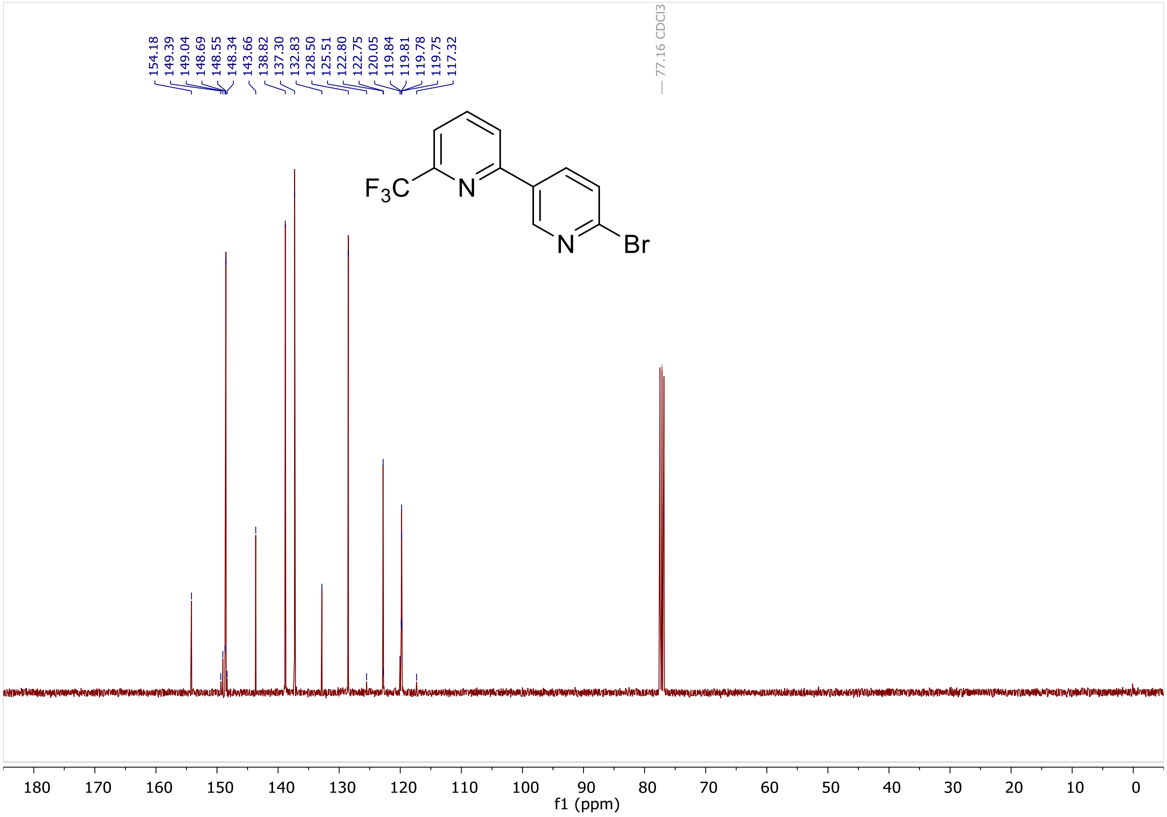

^{1}H NMR (400 MHz, CDCl$_3$): 11

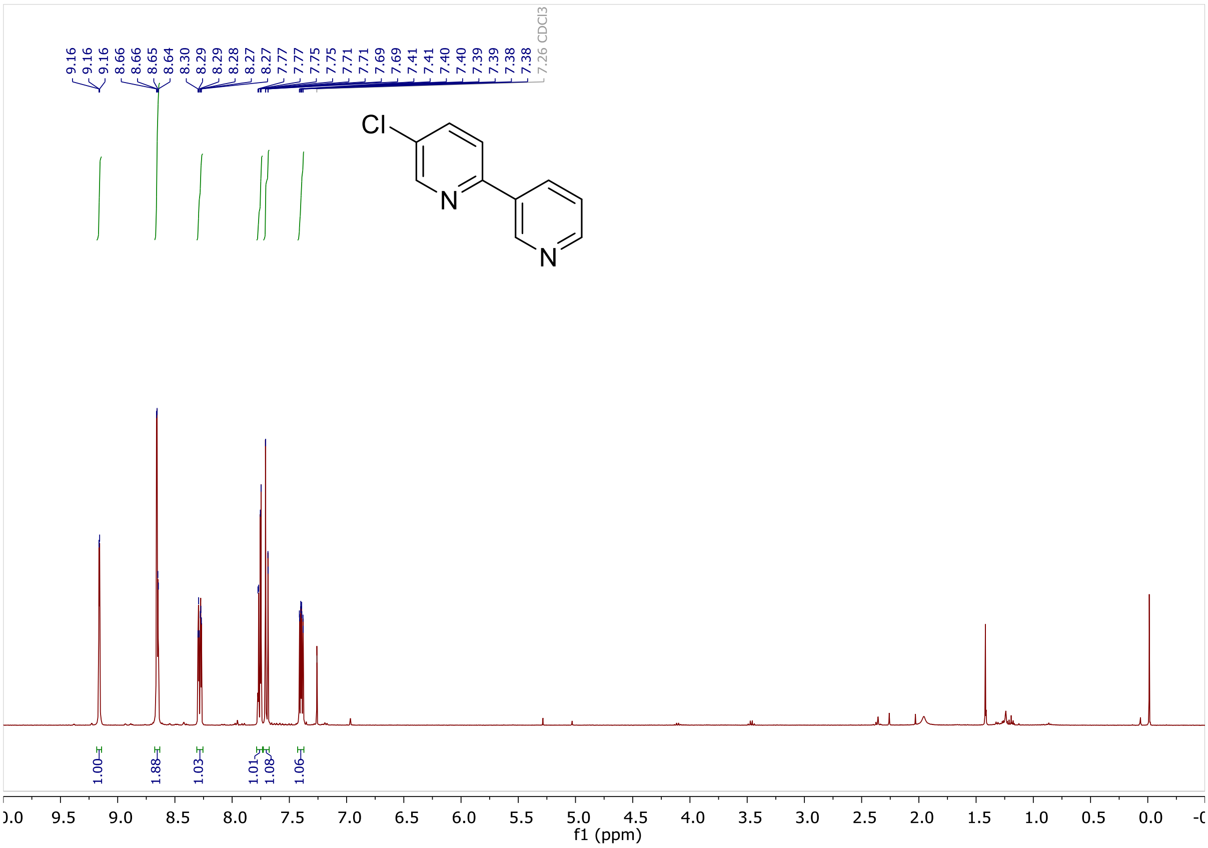

^{13}C NMR (101 MHz, CDCl$_3$): 11

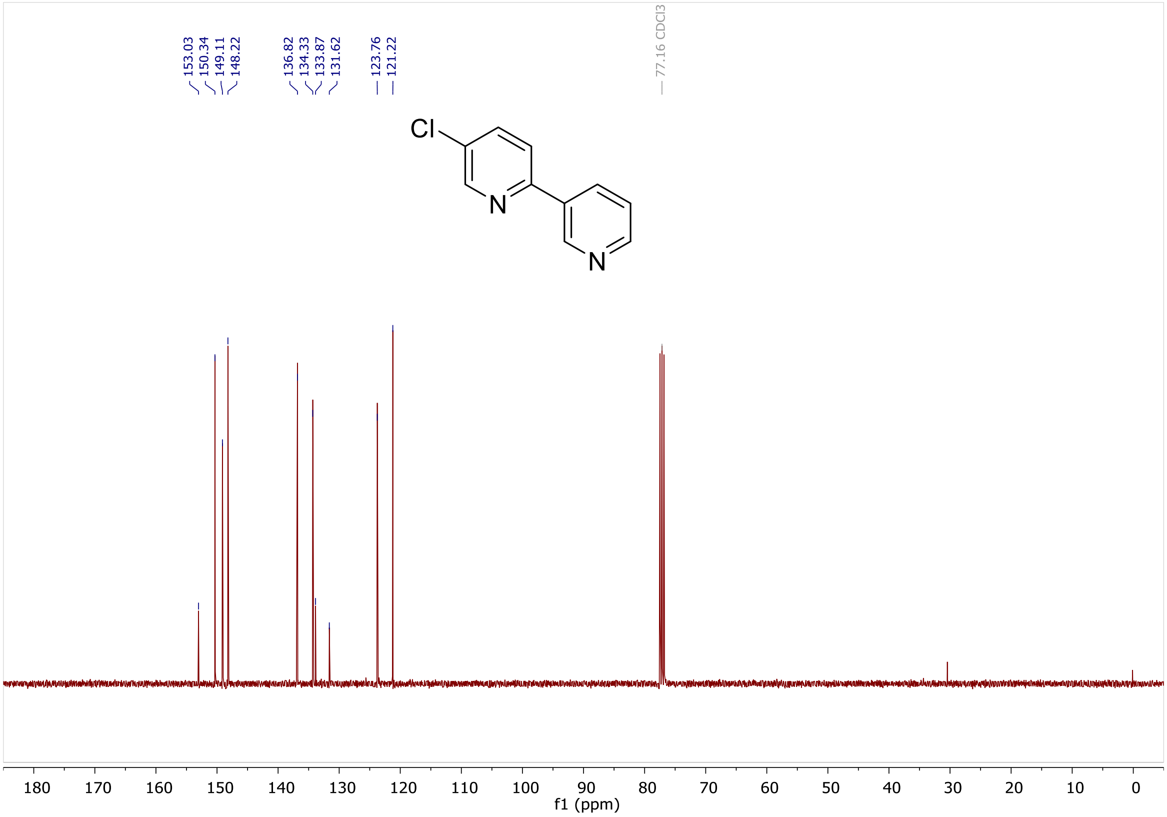

^{1}H NMR (400 MHz, CDCl$_3$): 12

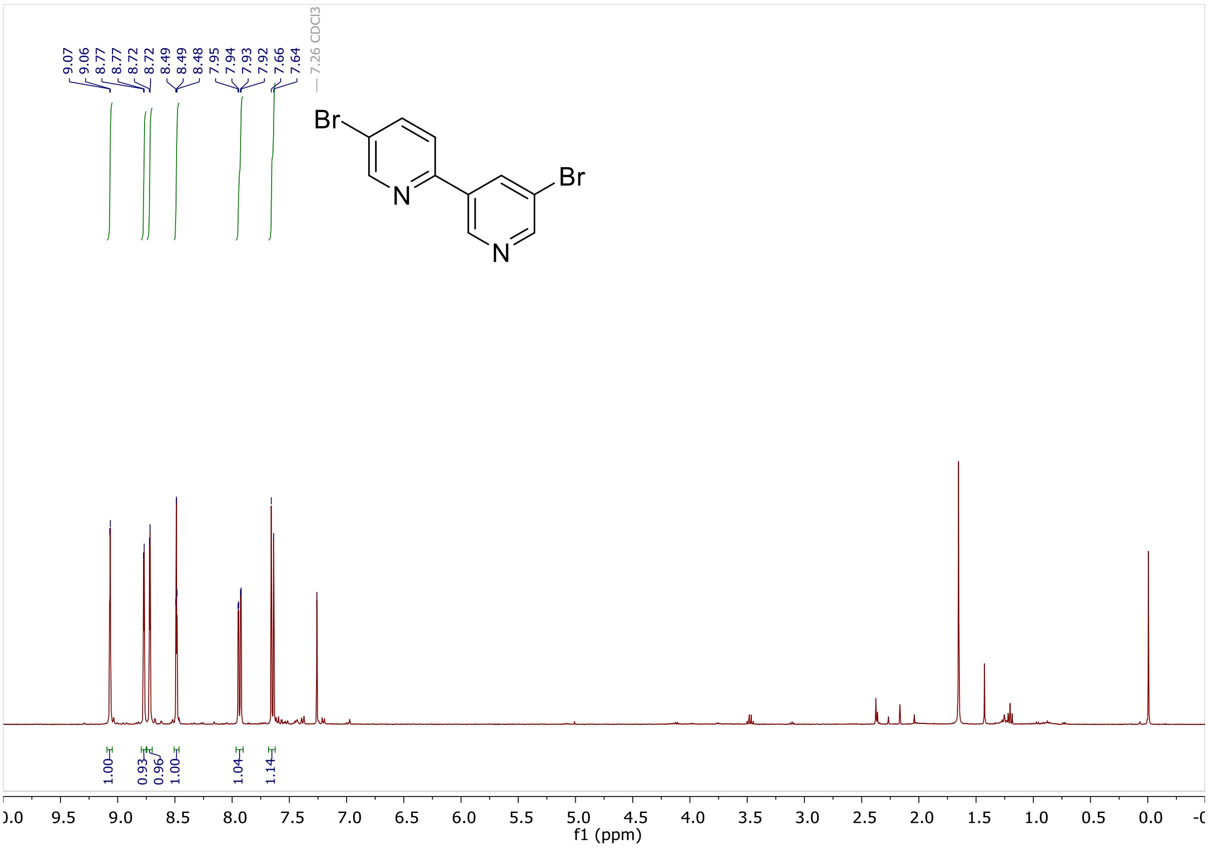

^{13}C NMR (101 MHz, CDCl$_3$): 12

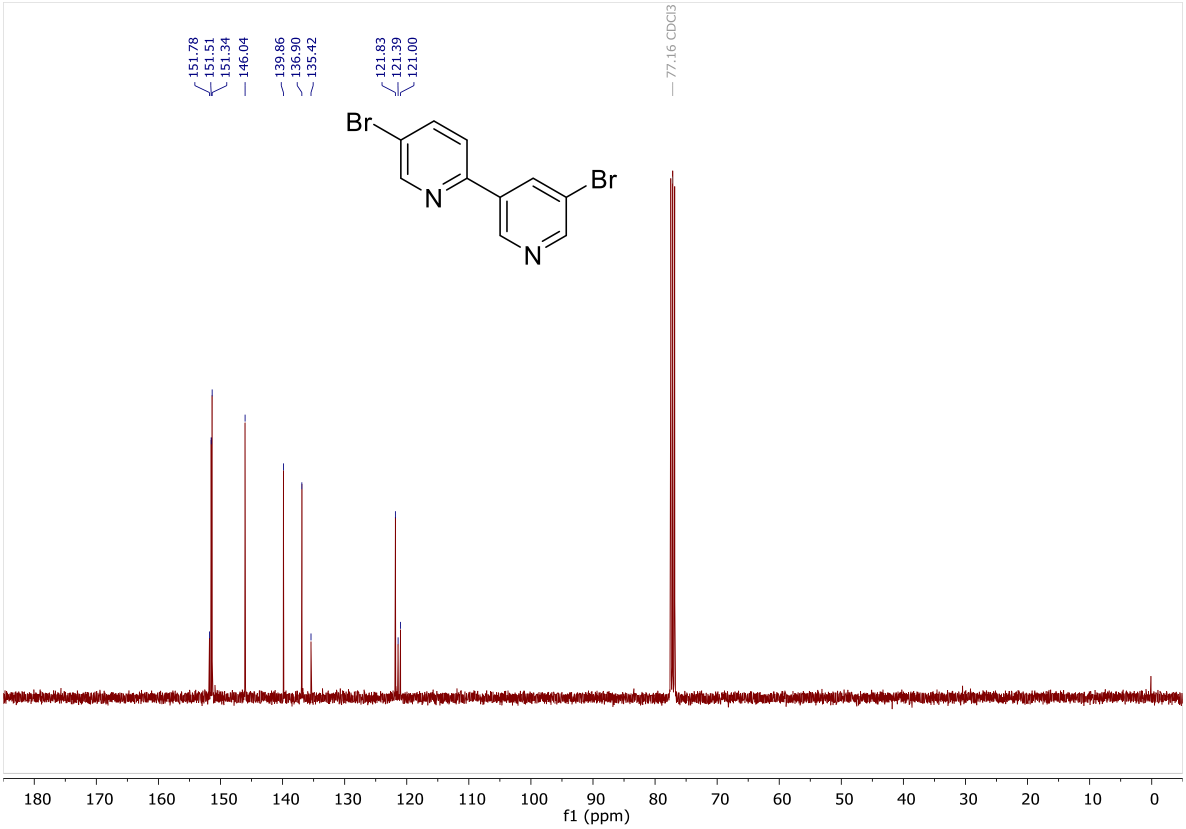

^{1}H NMR (500 MHz, $CDCl_3$): 13

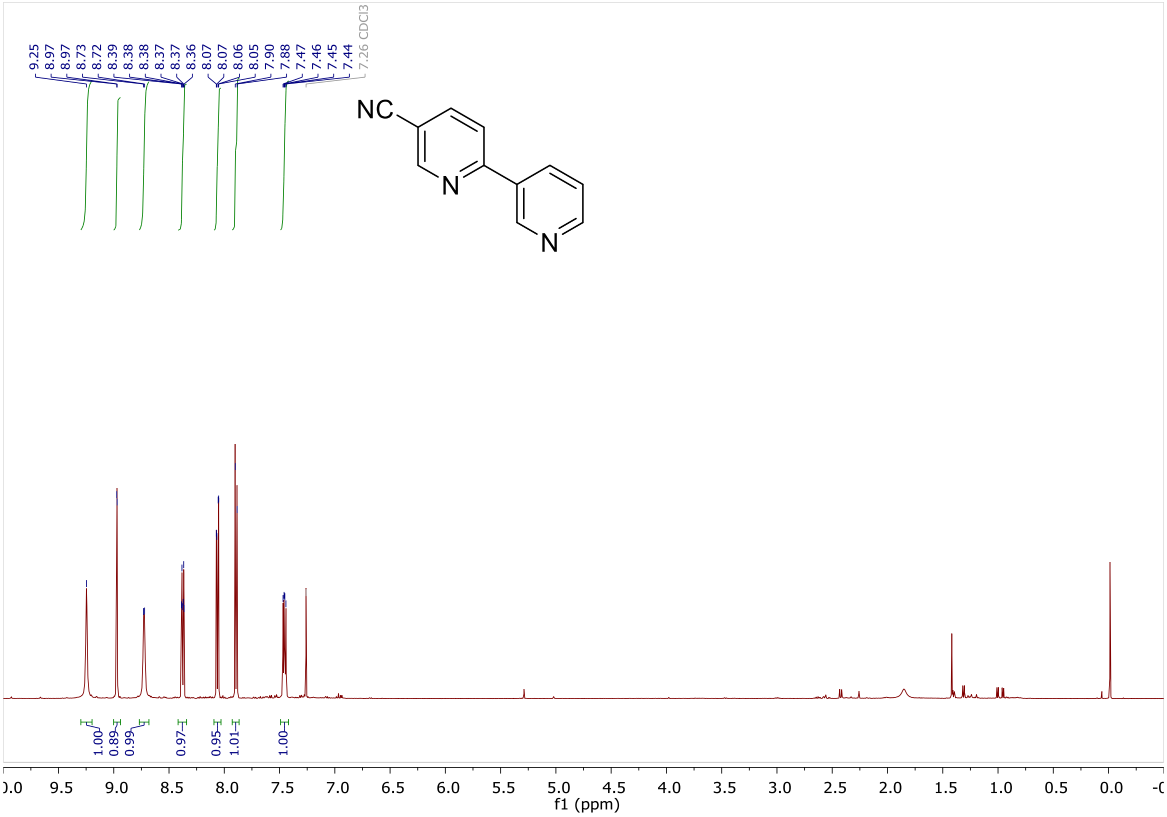

^{13}C NMR (126 MHz, $CDCl_3$): 13

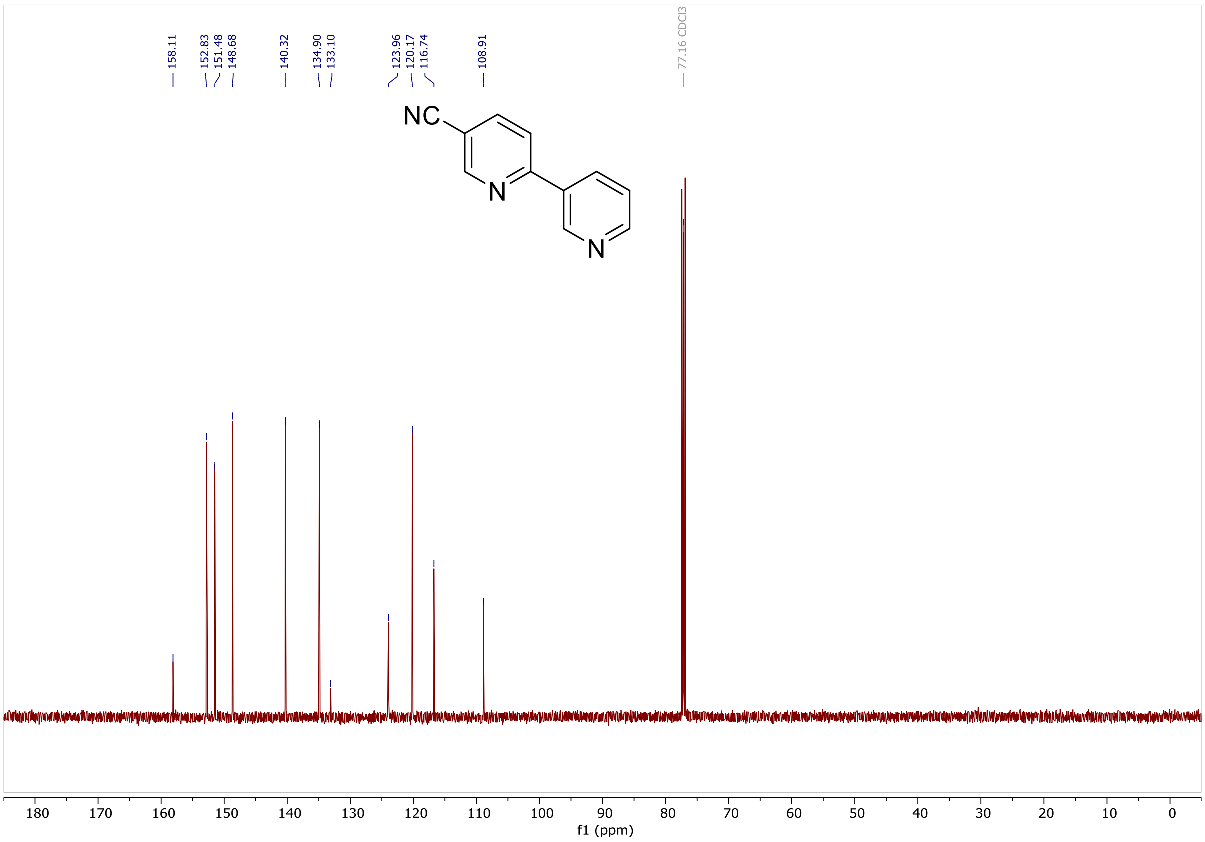

^{1}H NMR (500 MHz, $CDCl_3$): 14

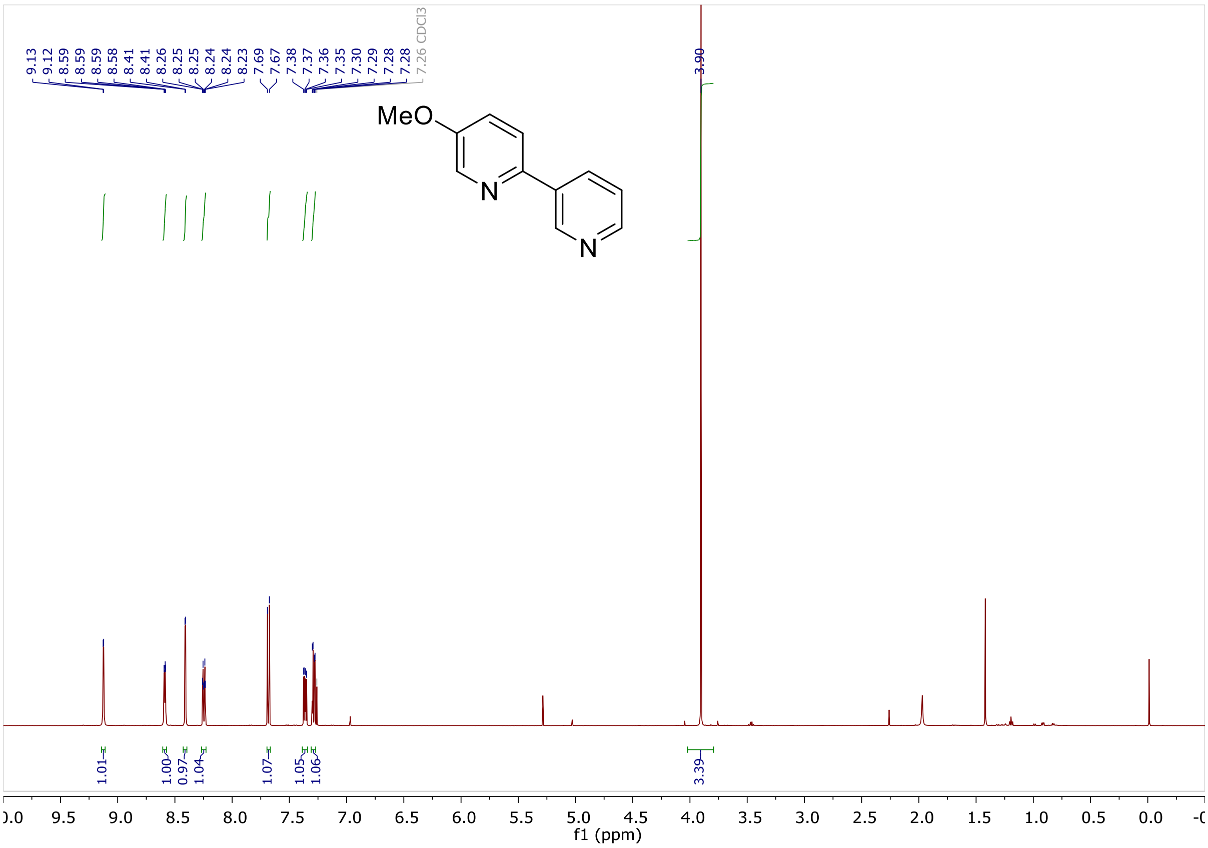

^{13}C NMR (126 MHz, $CDCl_3$): 14

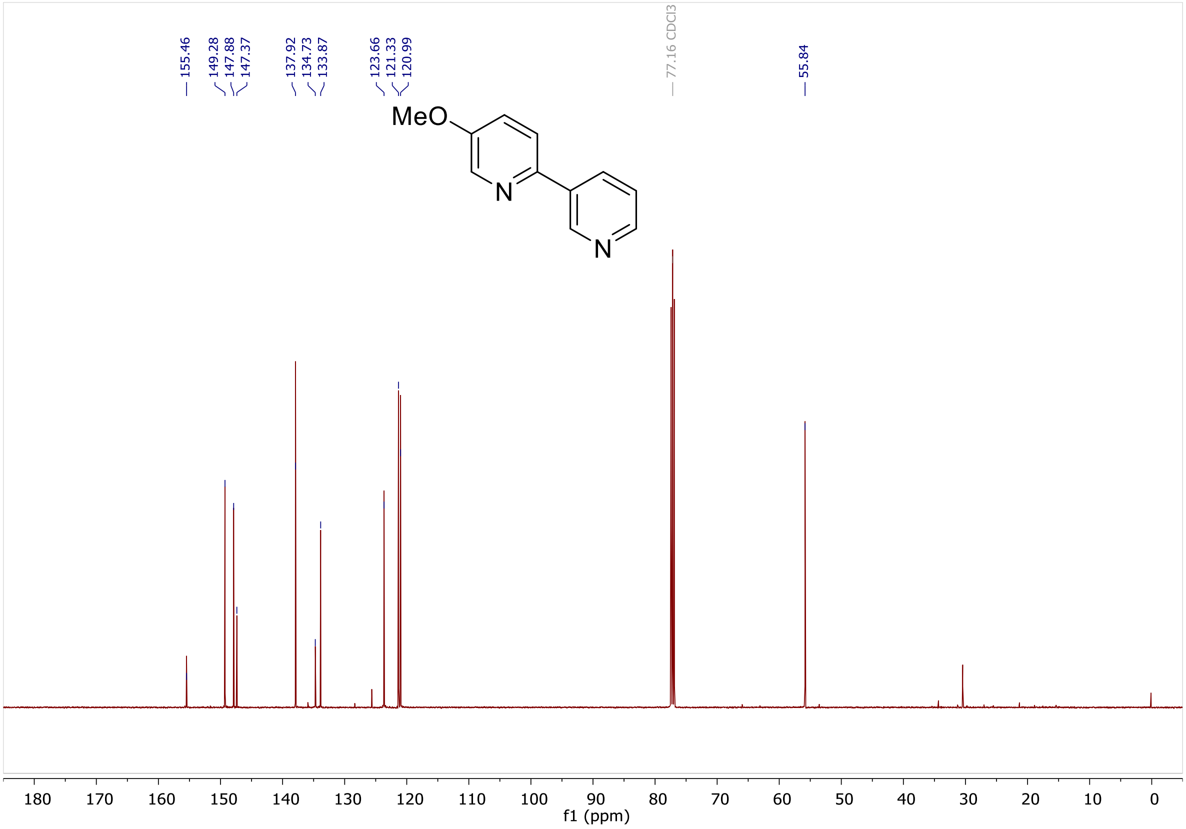

^{1}H NMR (500 MHz, $CDCl_3$): 15

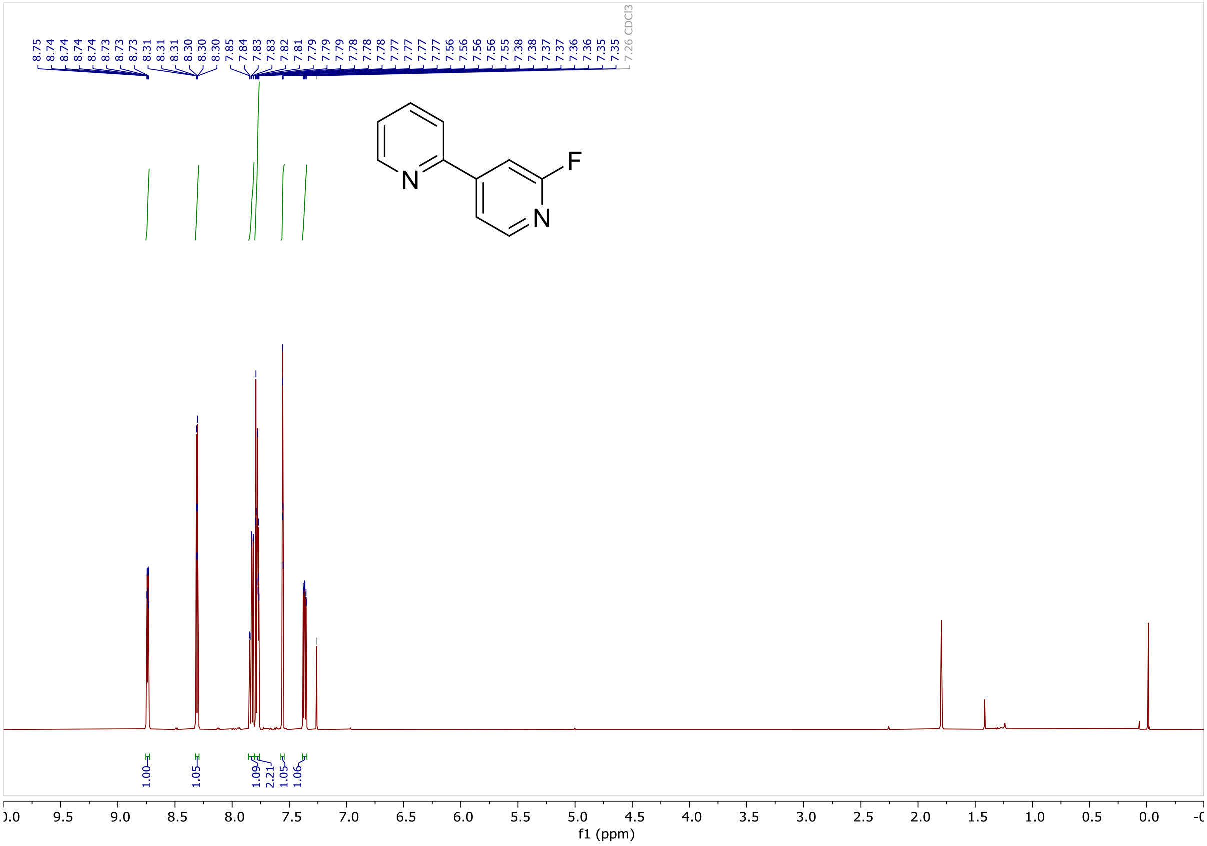

^{13}C NMR (126 MHz, $CDCl_3$): 15

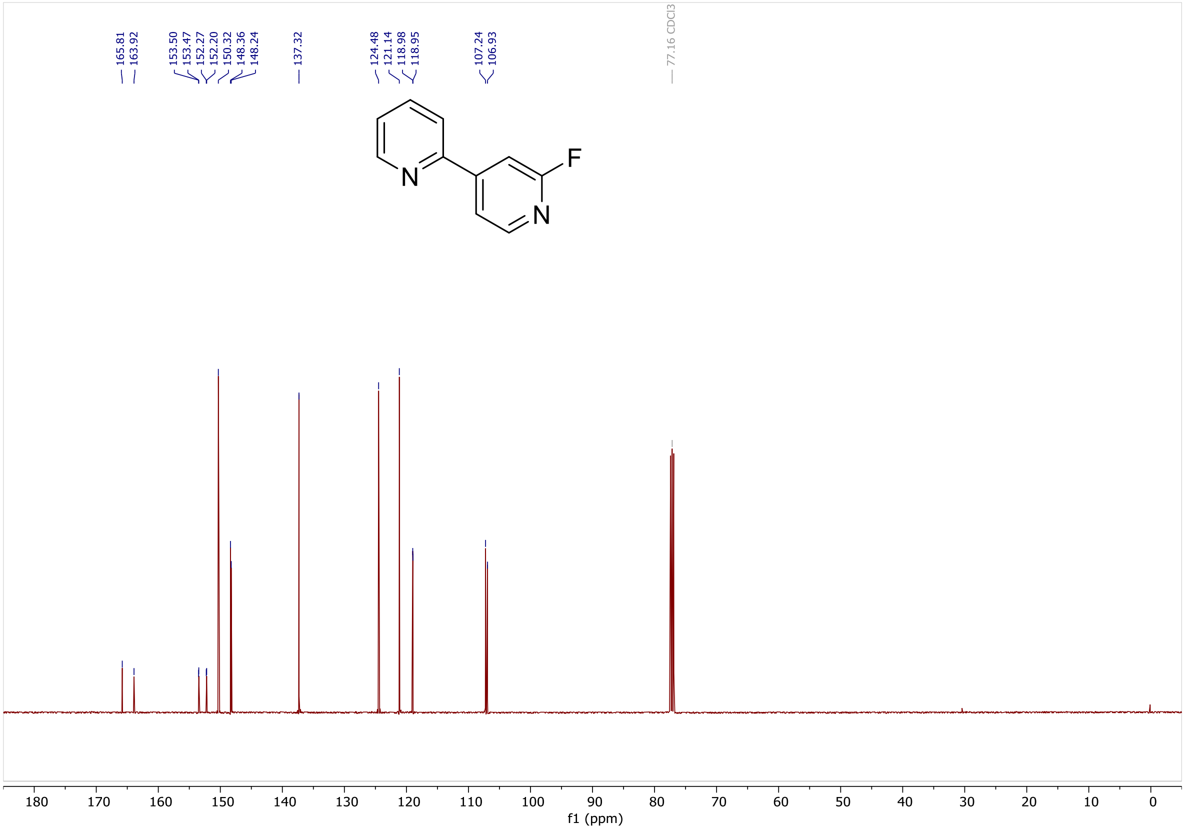

^{1}H NMR (500 MHz, $CDCl_3$): 16

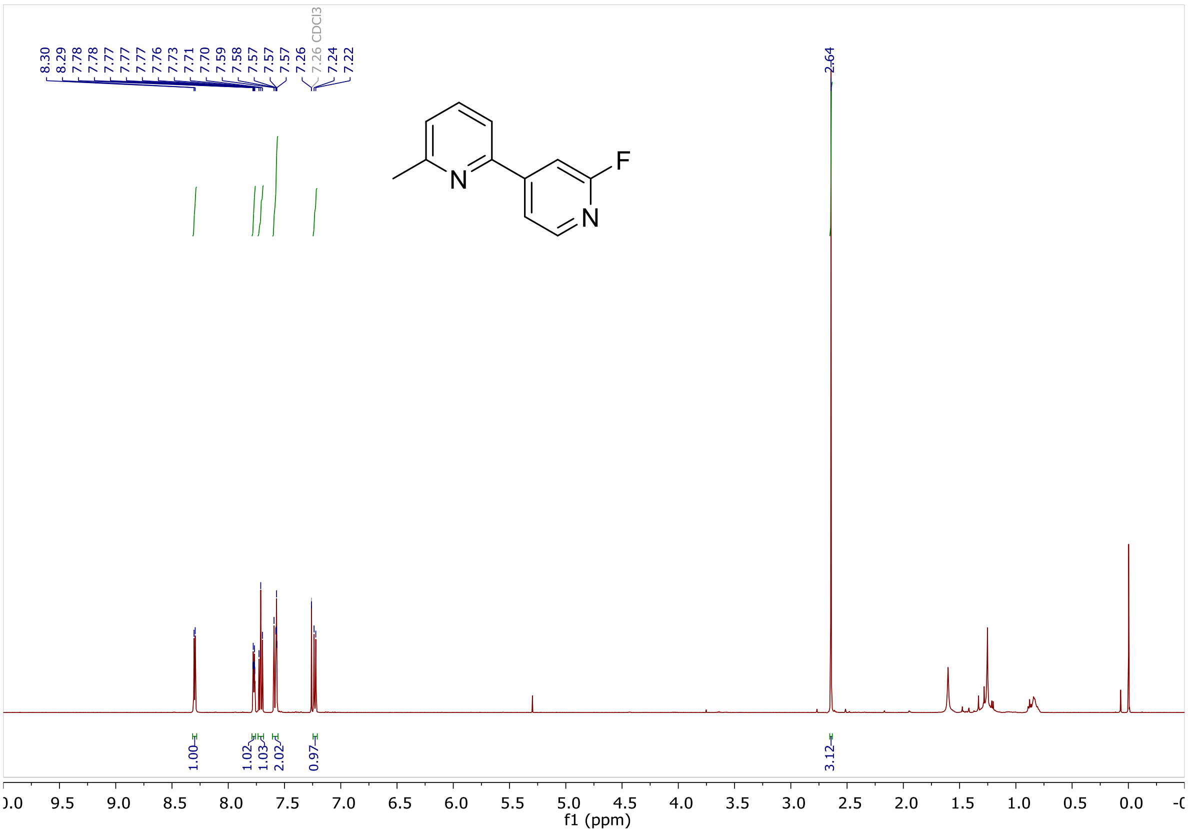

^{13}C NMR (126 MHz, $CDCl_3$): 16

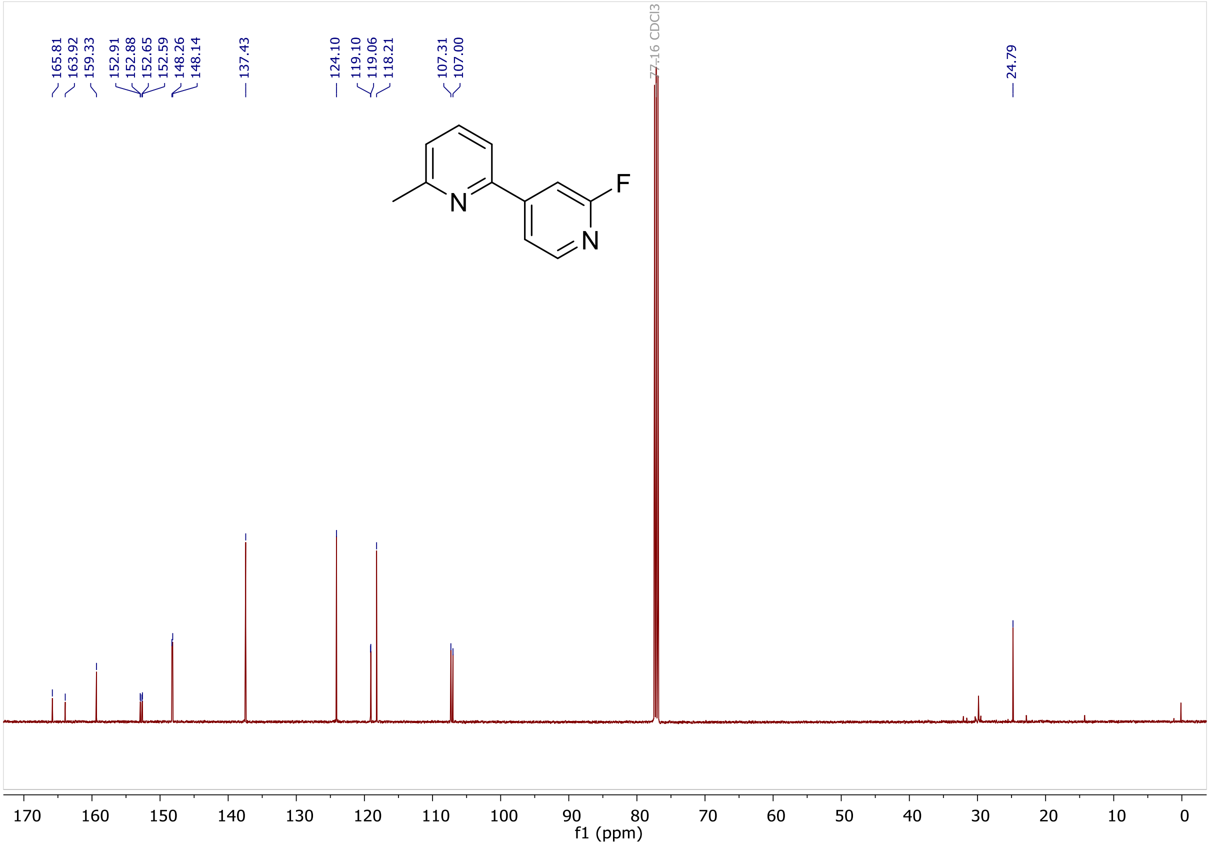

^{1}H NMR (500 MHz, CDCl$_3$): 17

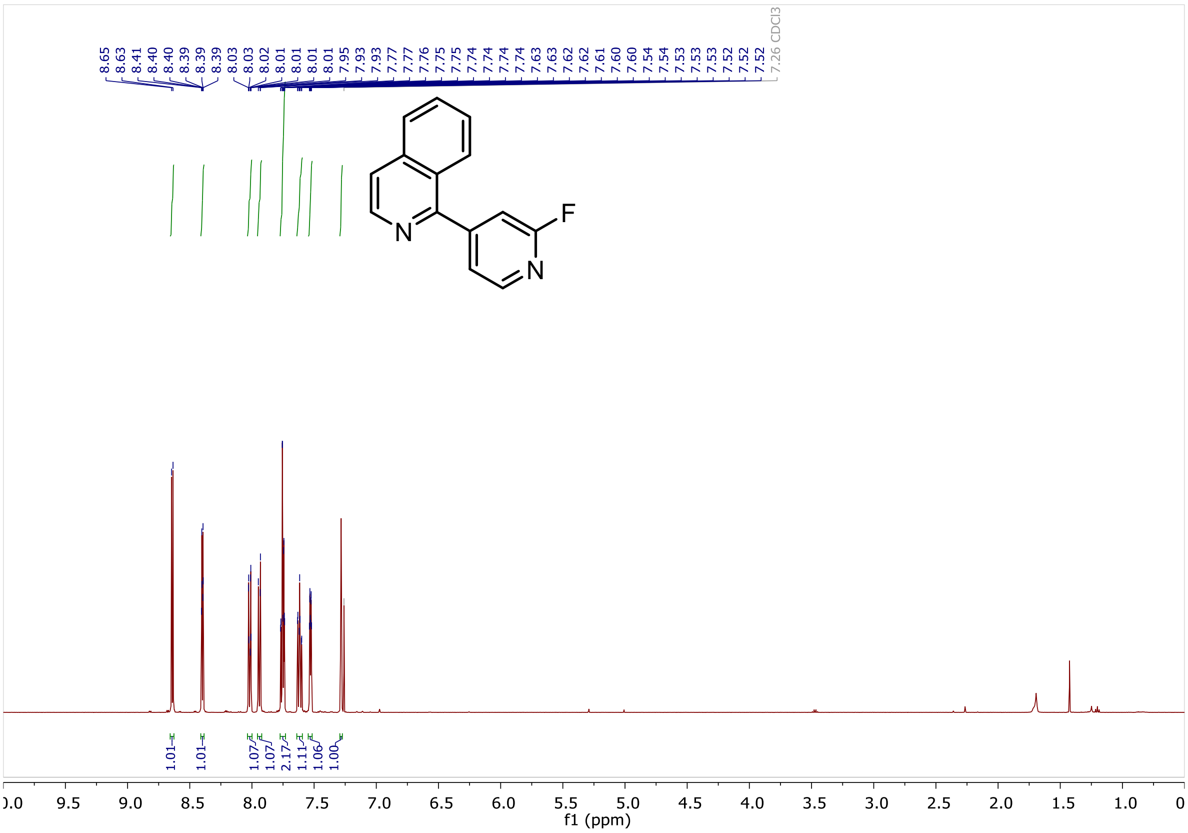

^{13}C NMR (126 MHz, CDCl$_3$): 17

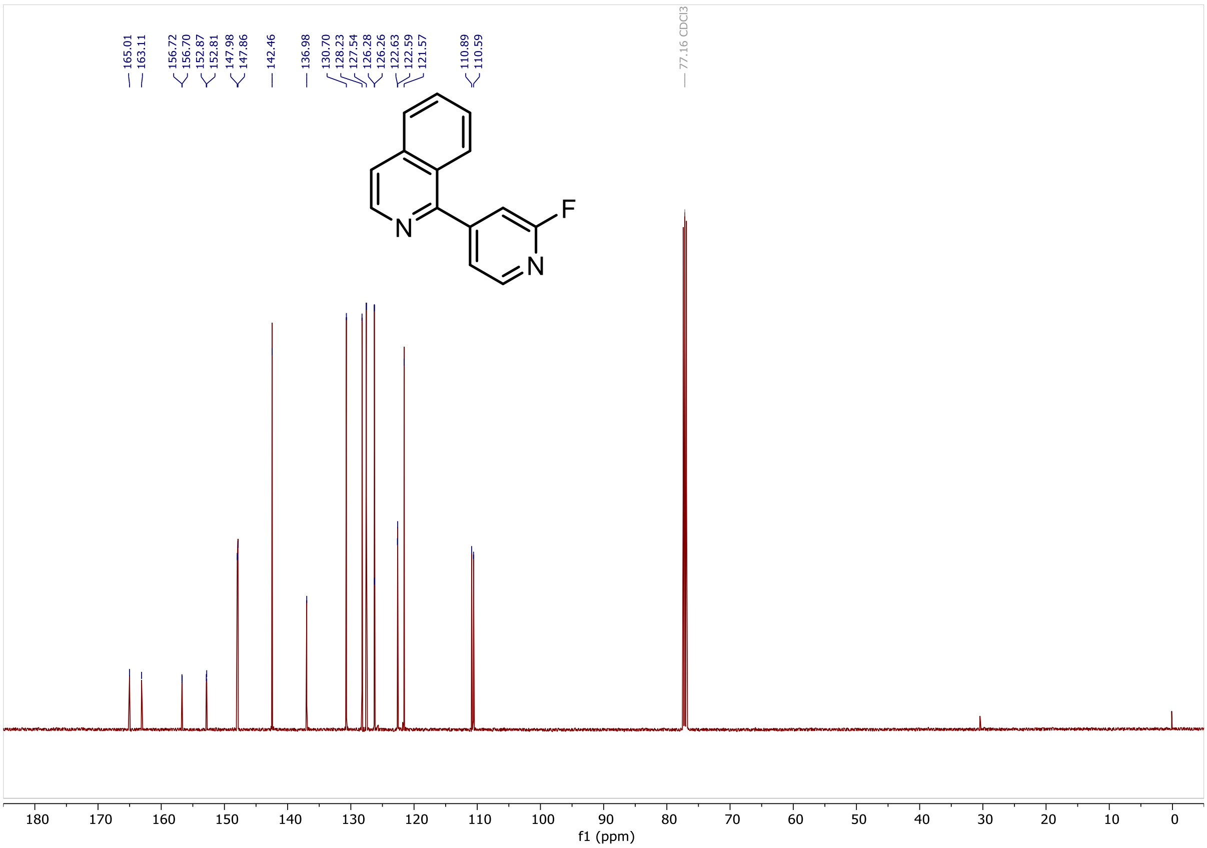

^{1}H NMR (500 MHz, $CDCl_3$): 18

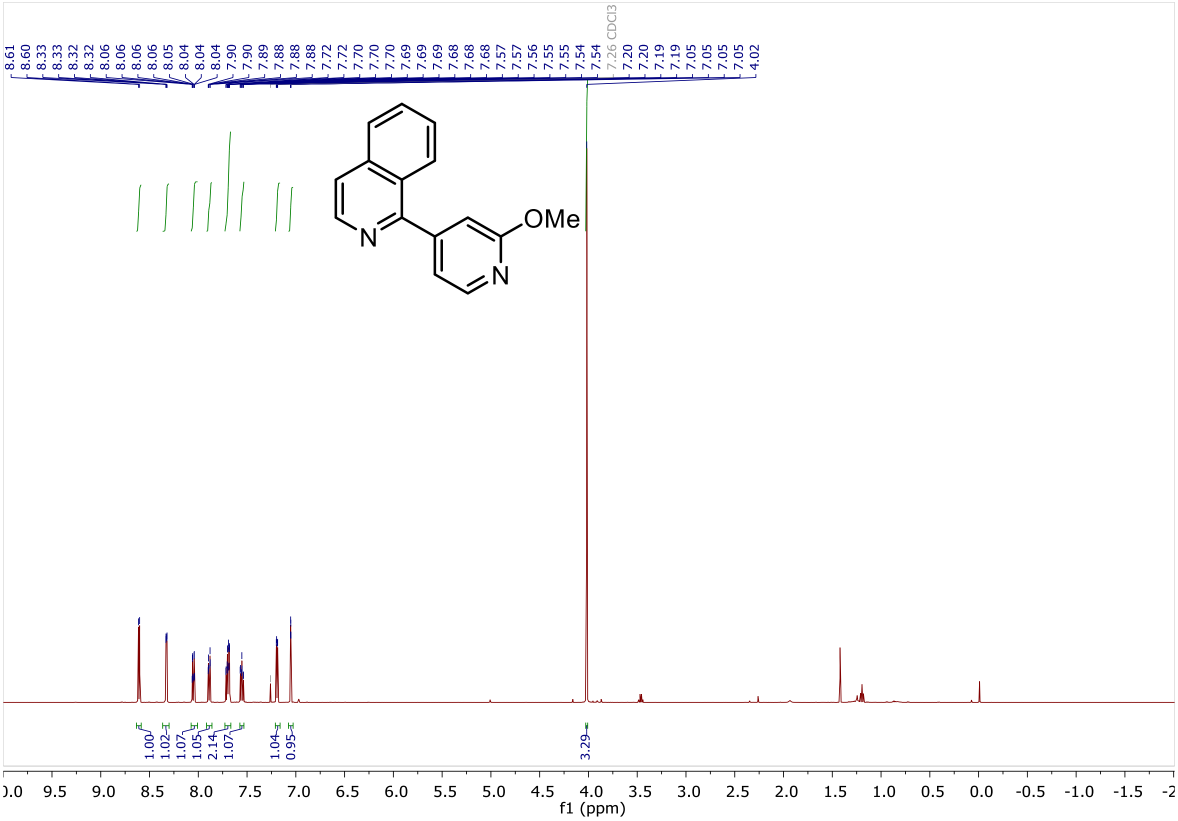

^{13}C NMR (126 MHz, $CDCl_3$): 18

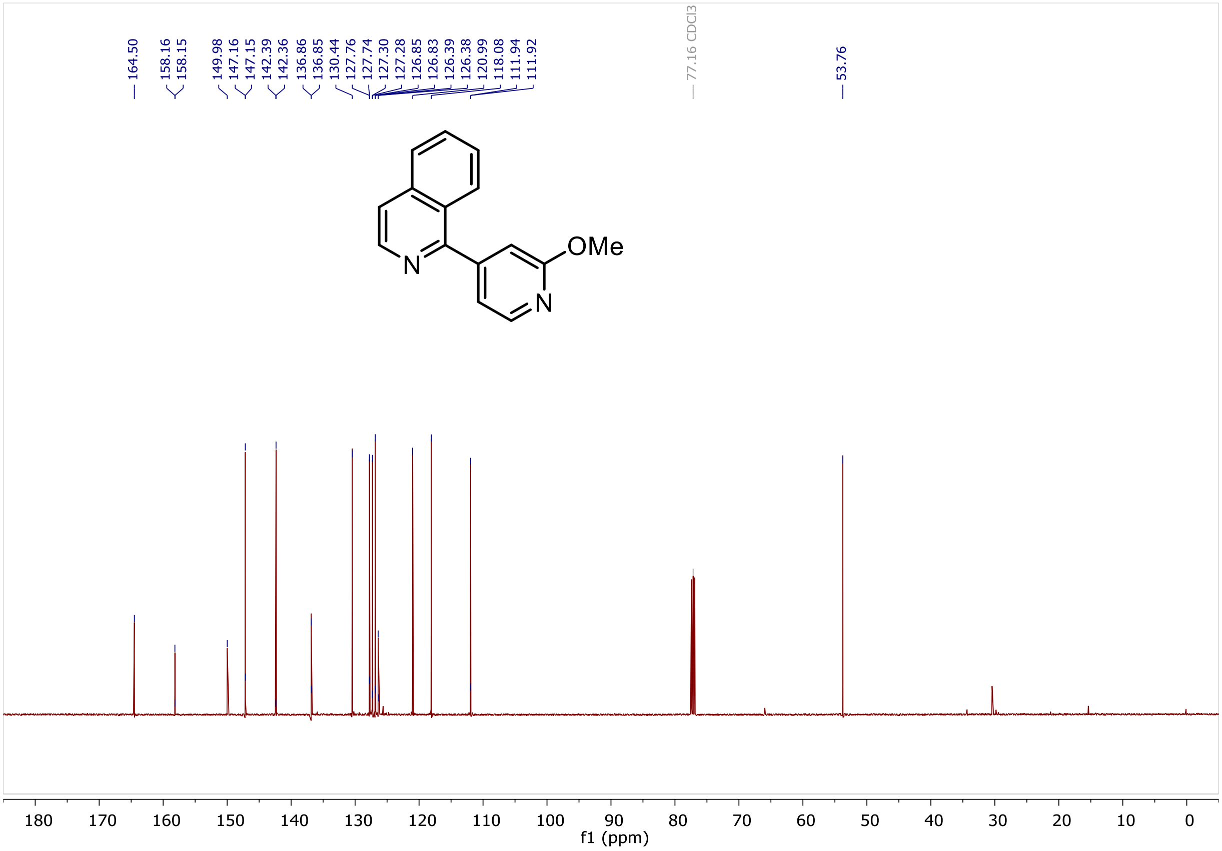

^{1}H NMR (500 MHz, $CDCl_3$): 19

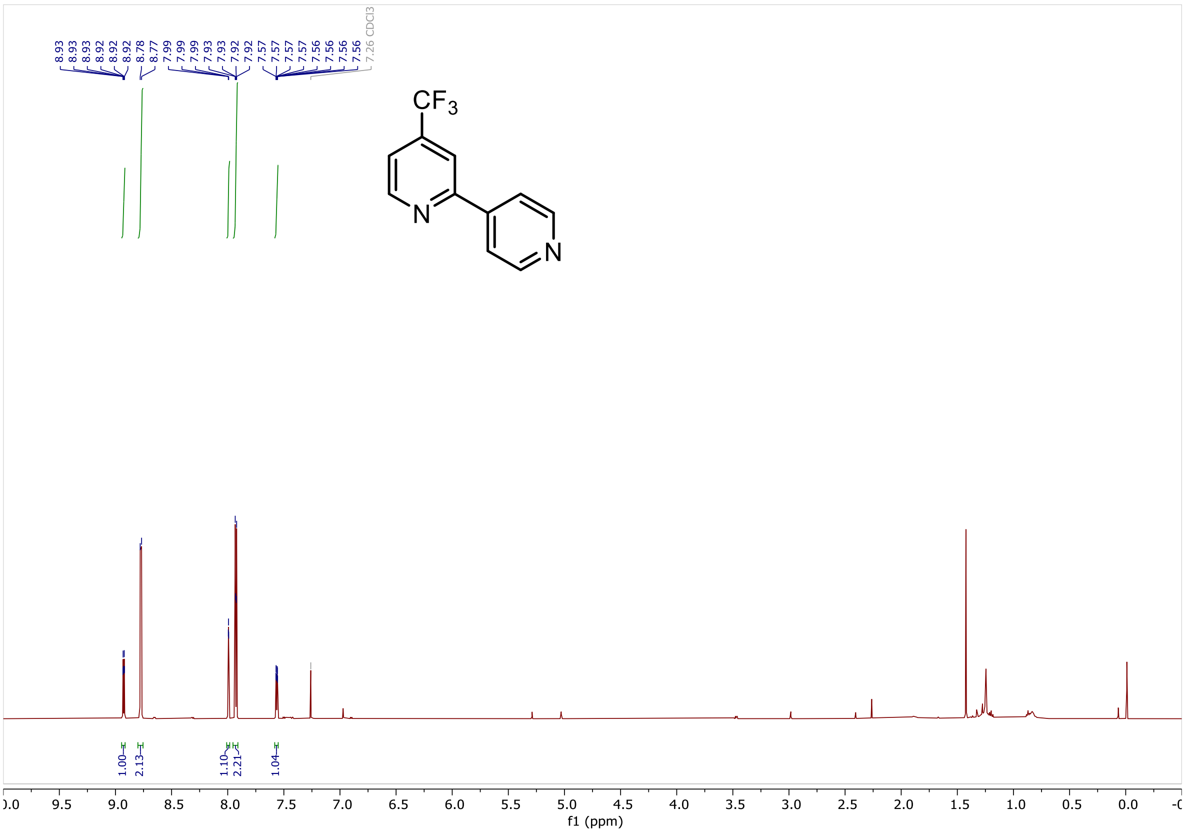

^{13}C NMR (126 MHz, $CDCl_3$): 19

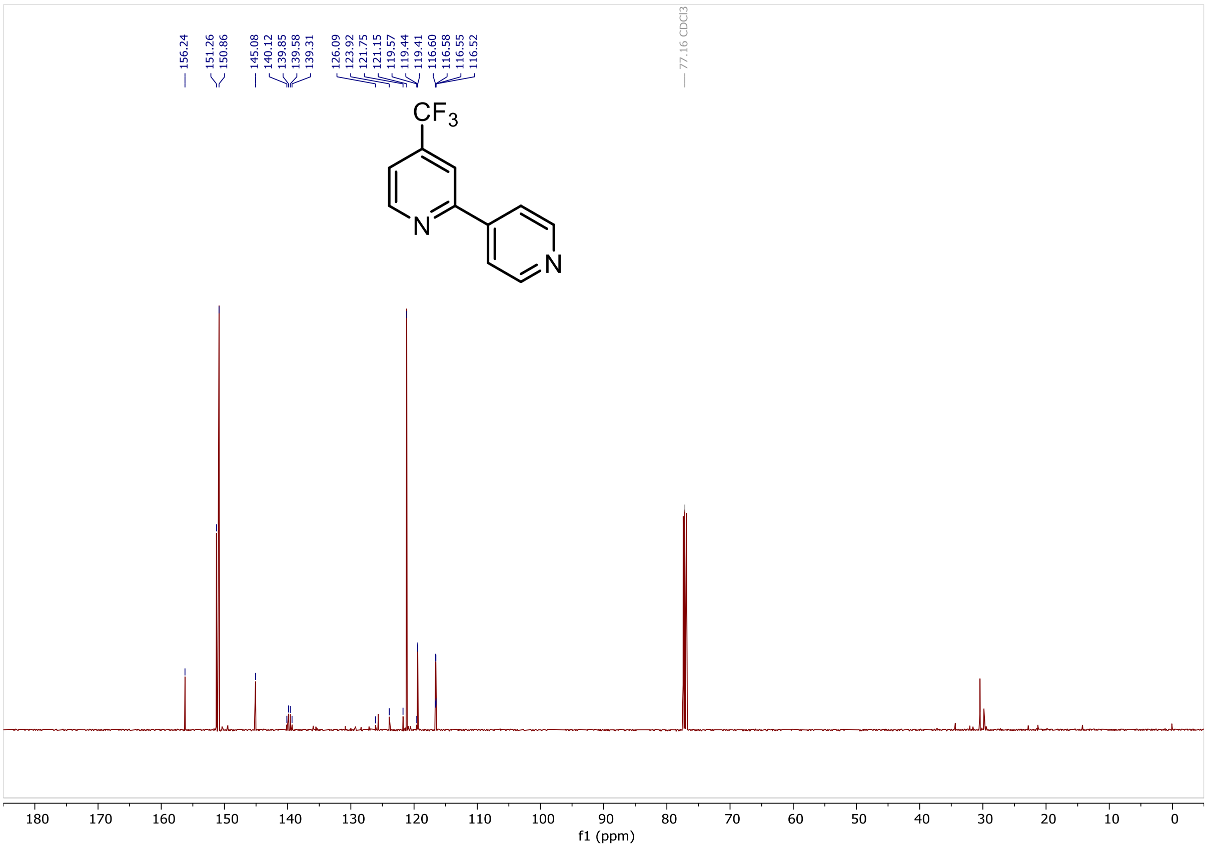

^{1}H NMR (500 MHz, $CDCl_3$): 20

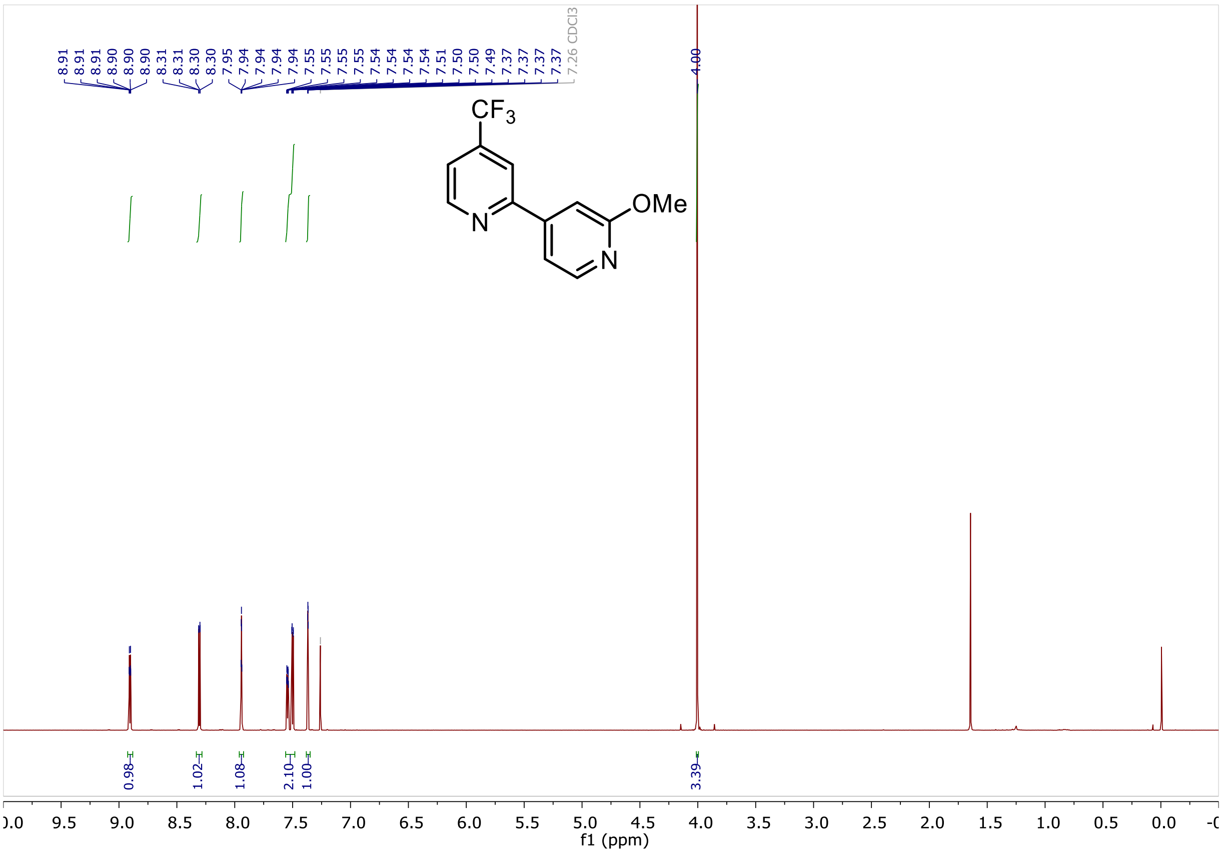

^{13}C NMR (126 MHz, $CDCl_3$): 20

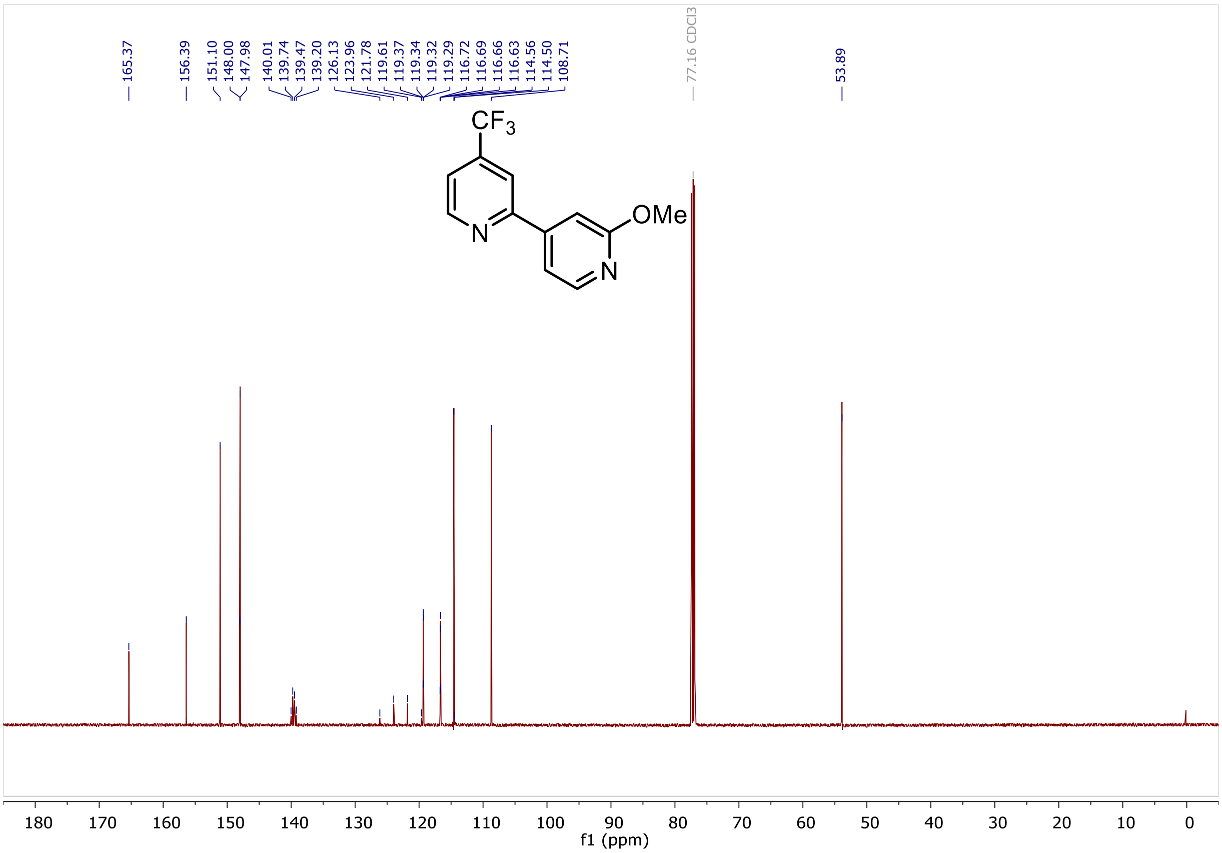

^{1}H NMR (500 MHz, $CDCl_3$): 21

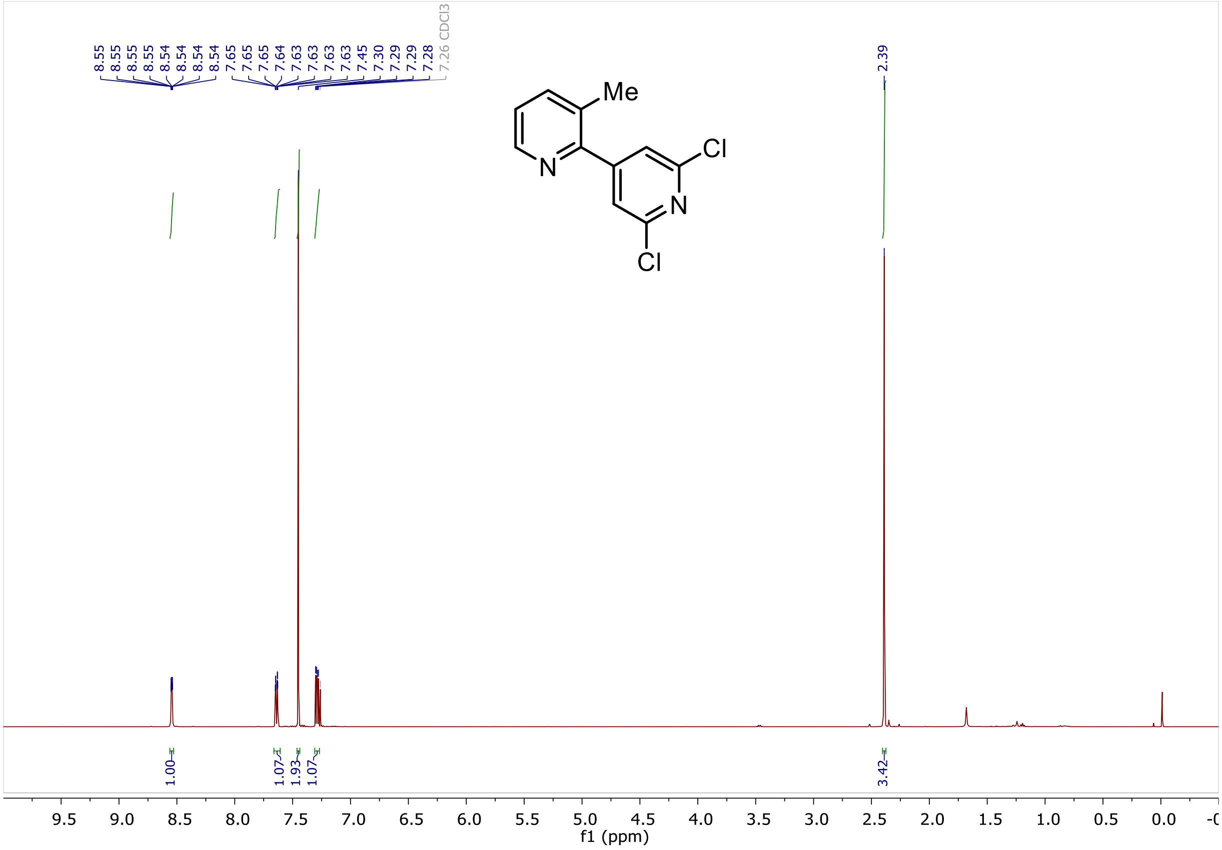

^{13}C NMR (126 MHz, $CDCl_3$): 21

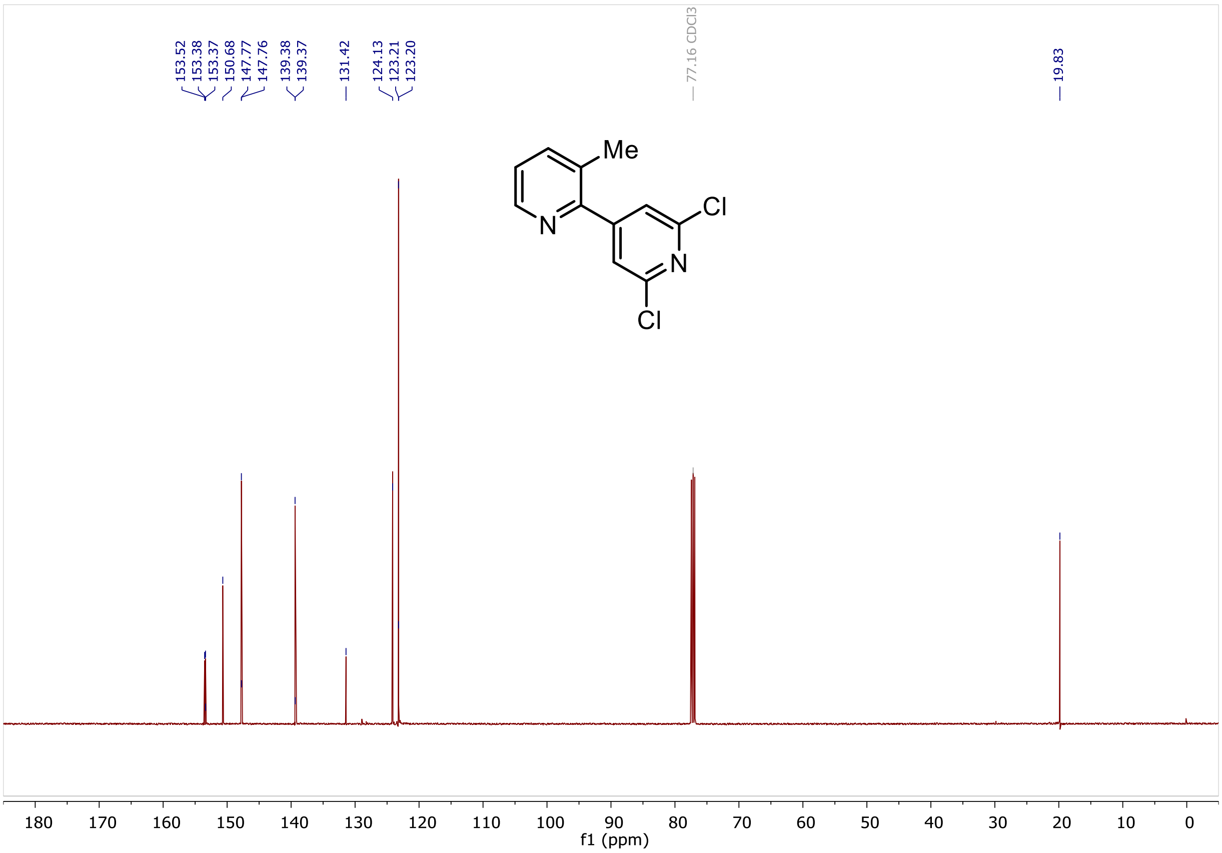

^{1}H NMR (500 MHz, $CDCl_3$): 22

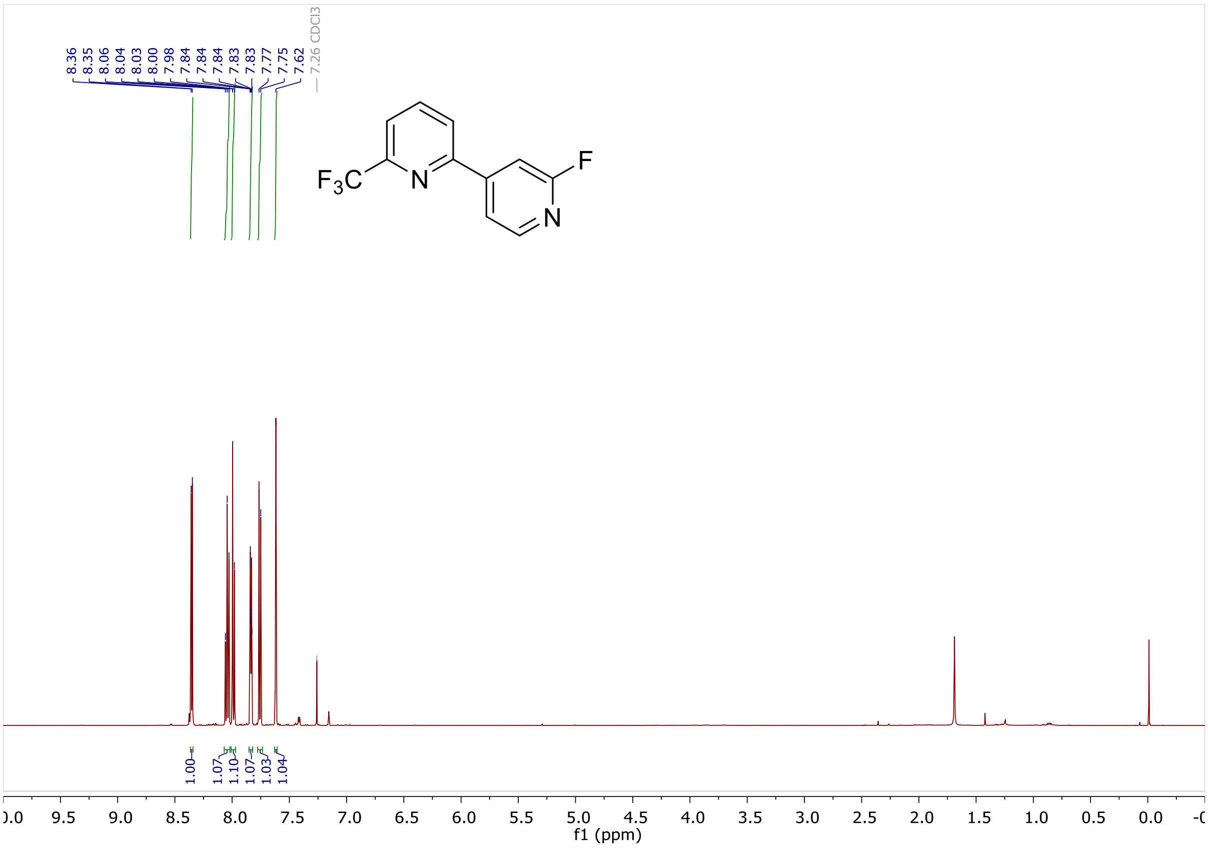

^{13}C NMR (126 MHz, $CDCl_3$): 22

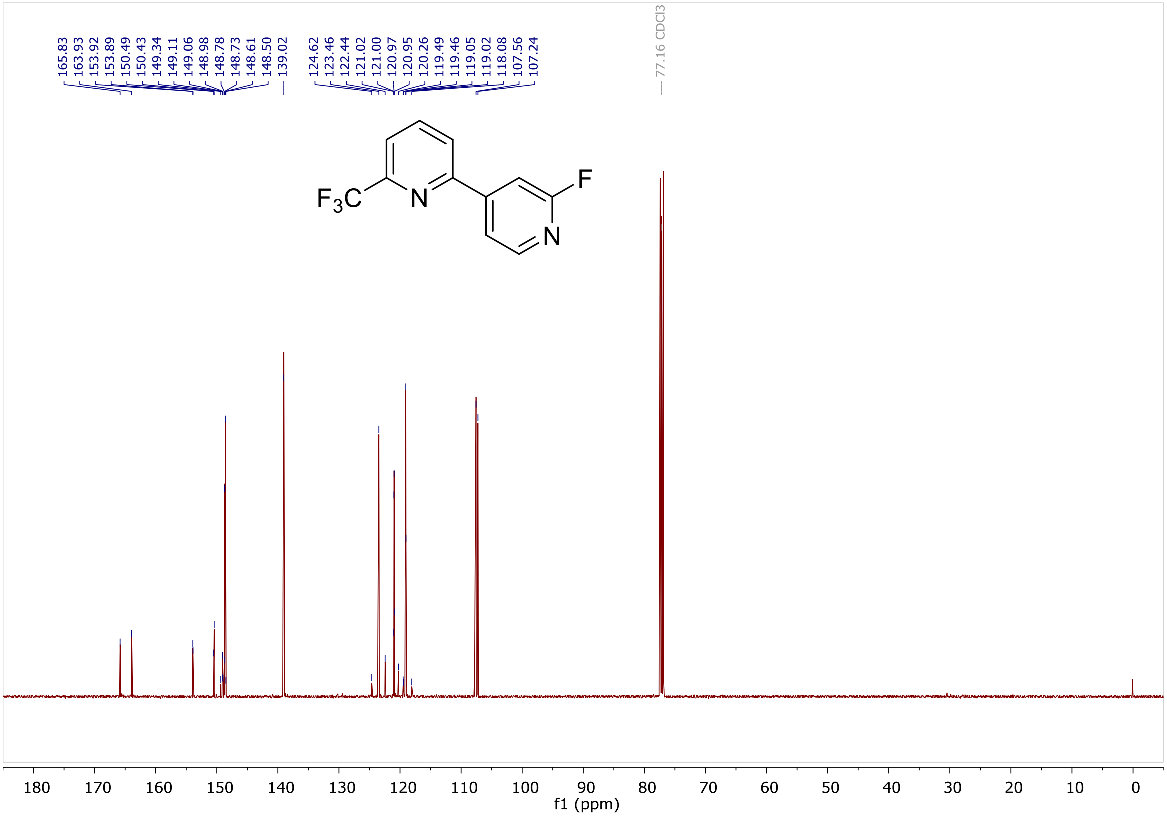

^{1}H NMR (400 MHz, $CDCl_3$): 23

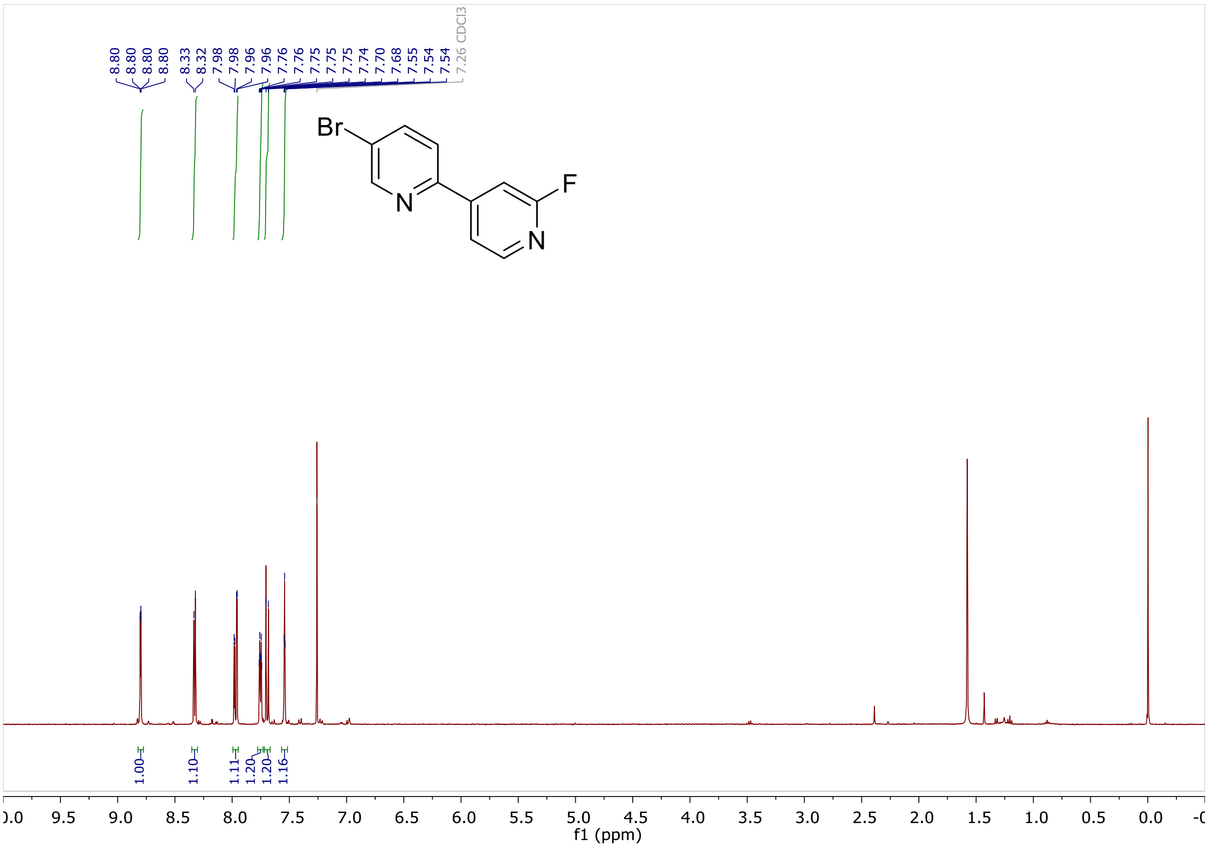

^{13}C NMR (126 MHz, $CDCl_3$): 23

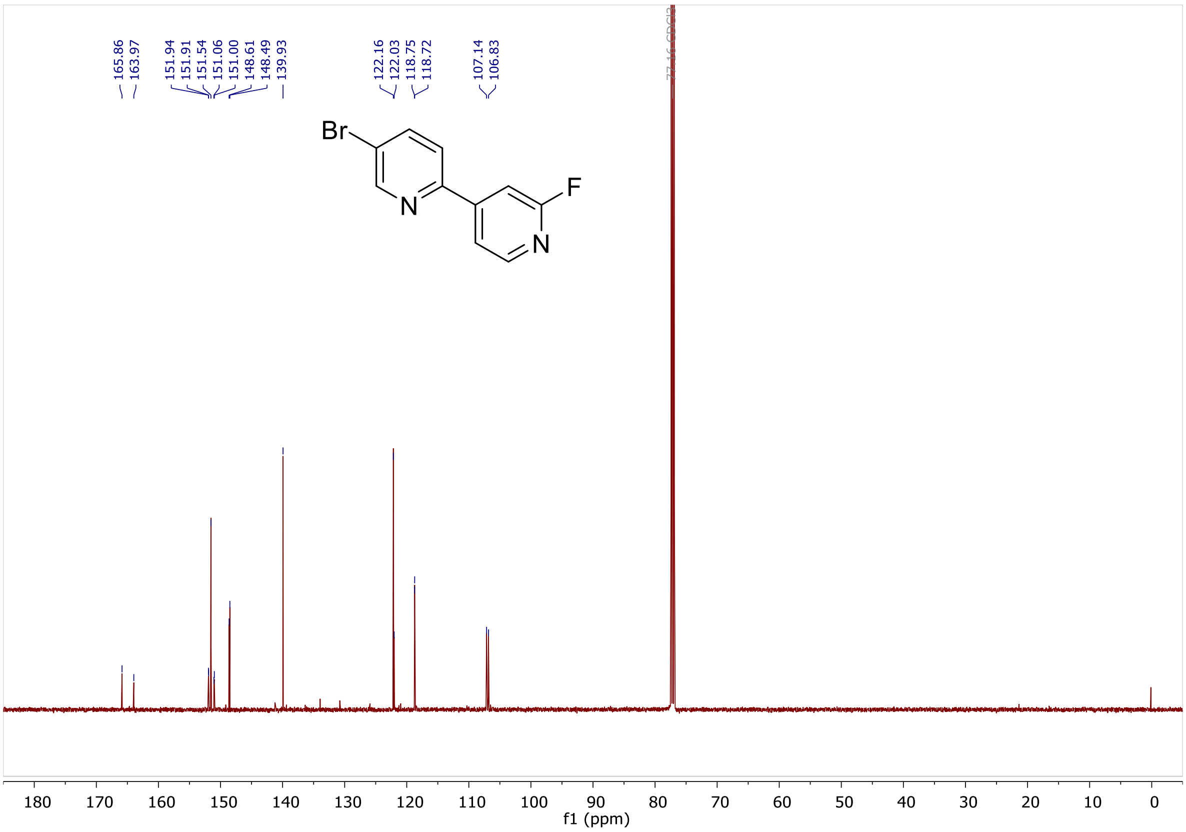

^{1}H NMR (400 MHz, $CDCl_3$): 24

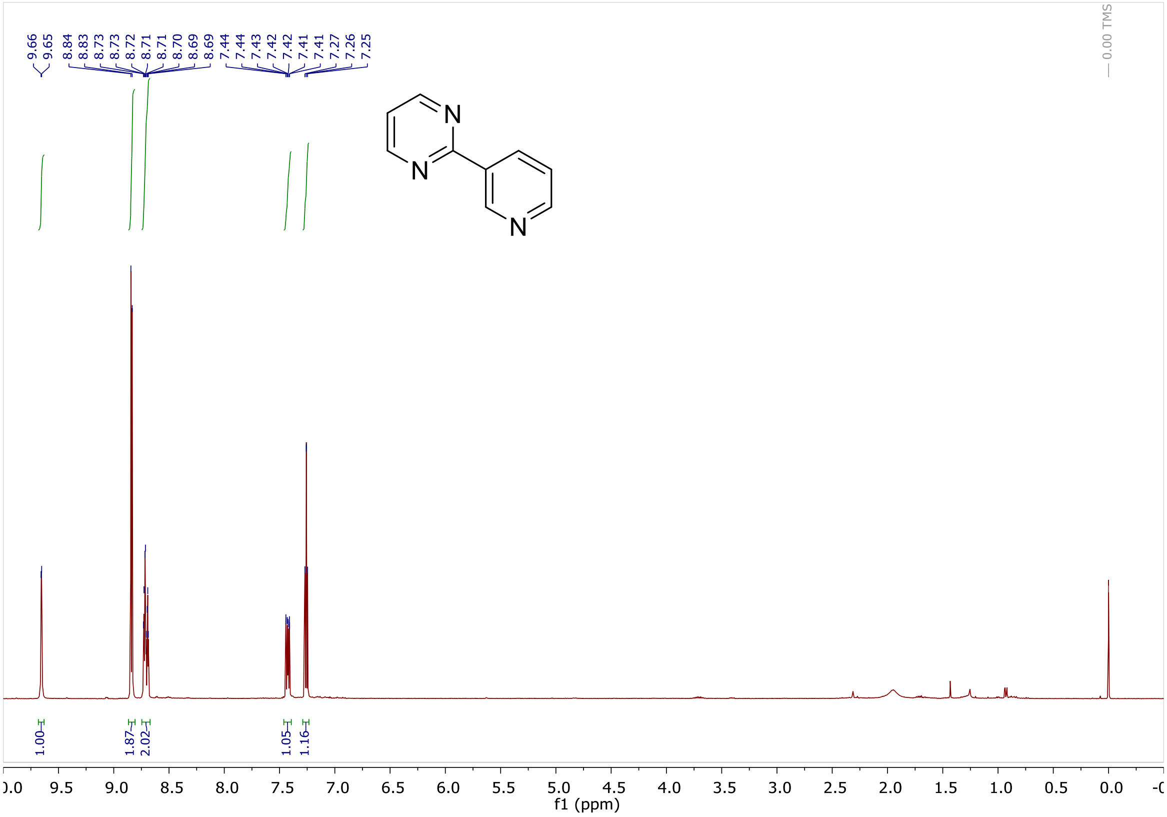

^{13}C NMR (101 MHz, $CDCl_3$): 24

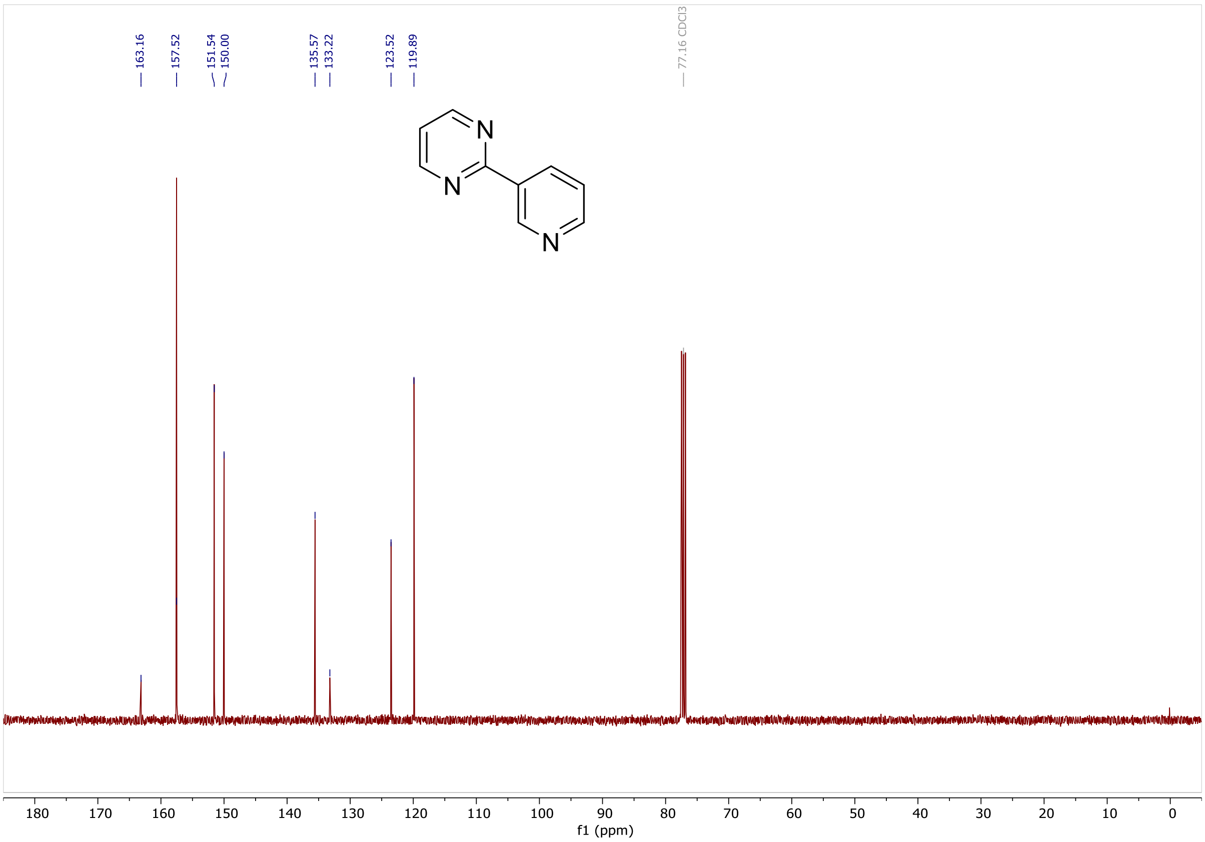

^{1}H NMR (400 MHz, $CDCl_3$): 25

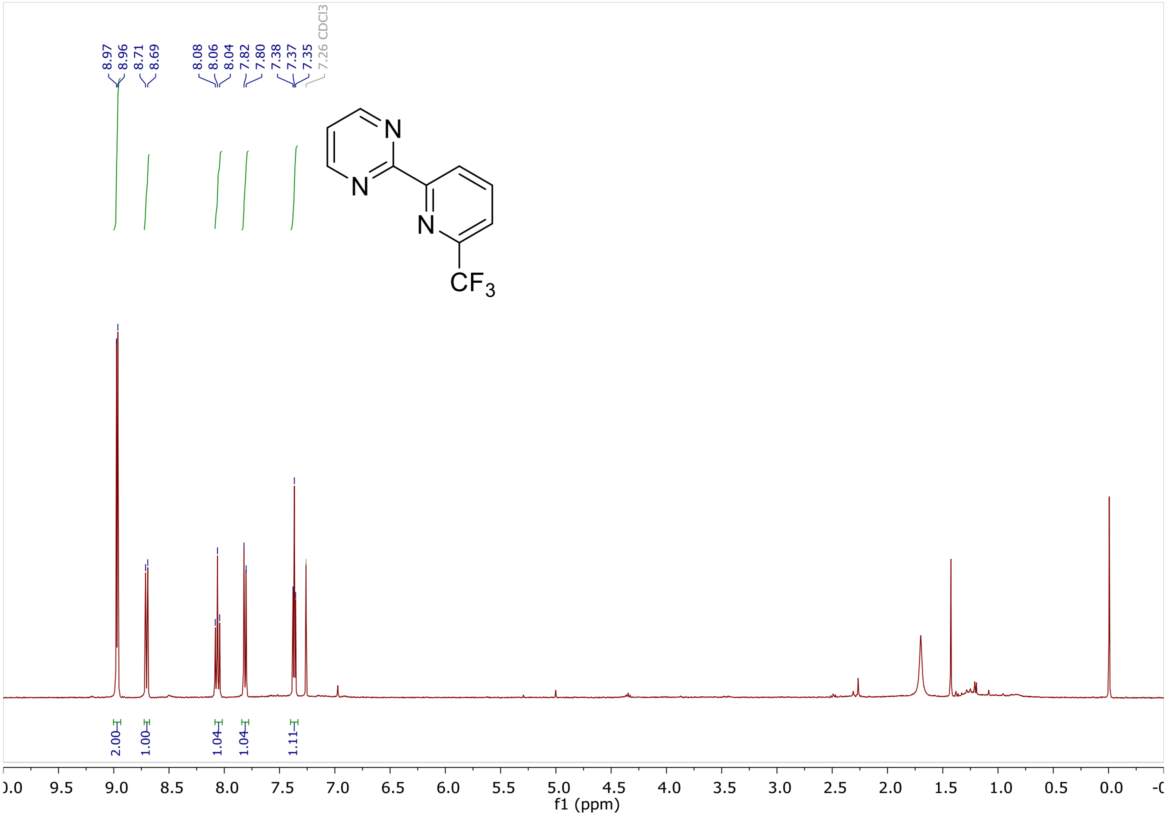

^{13}C NMR (101 MHz, $CDCl_3$): 25

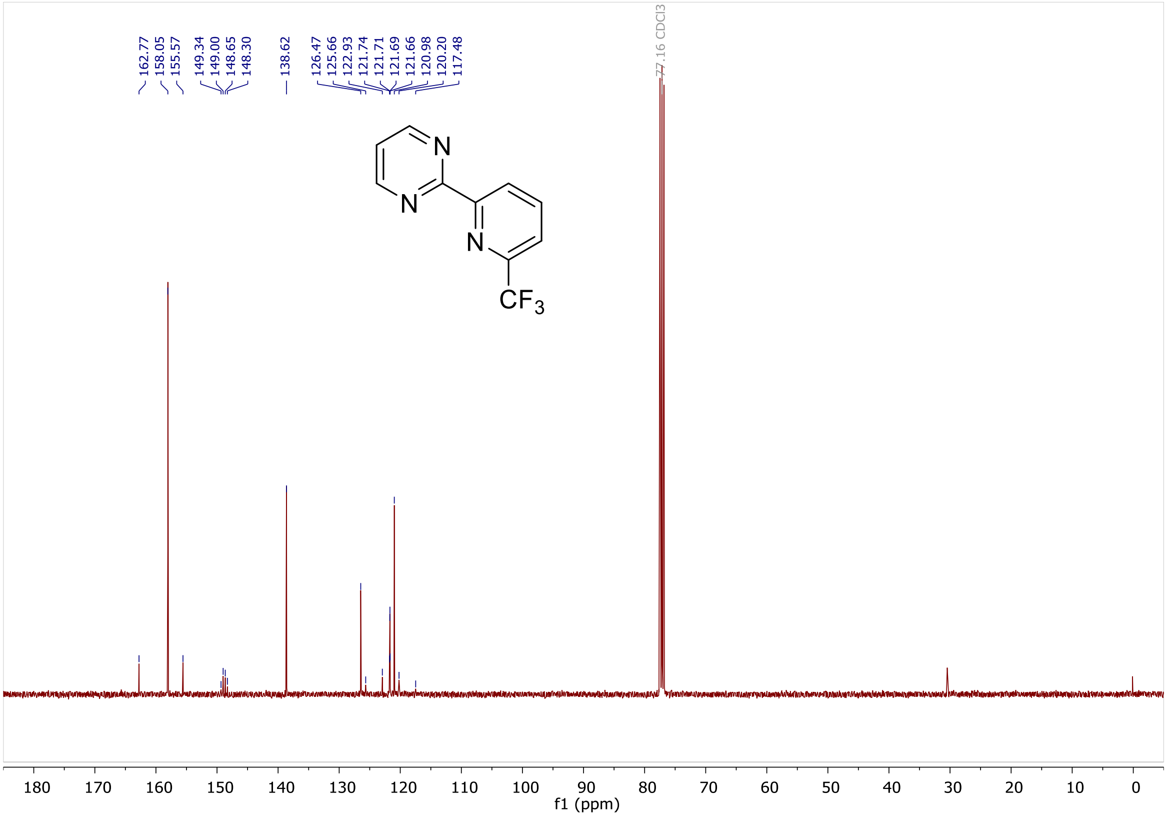

^{1}H NMR (500 MHz, CDCl$_3$): 26

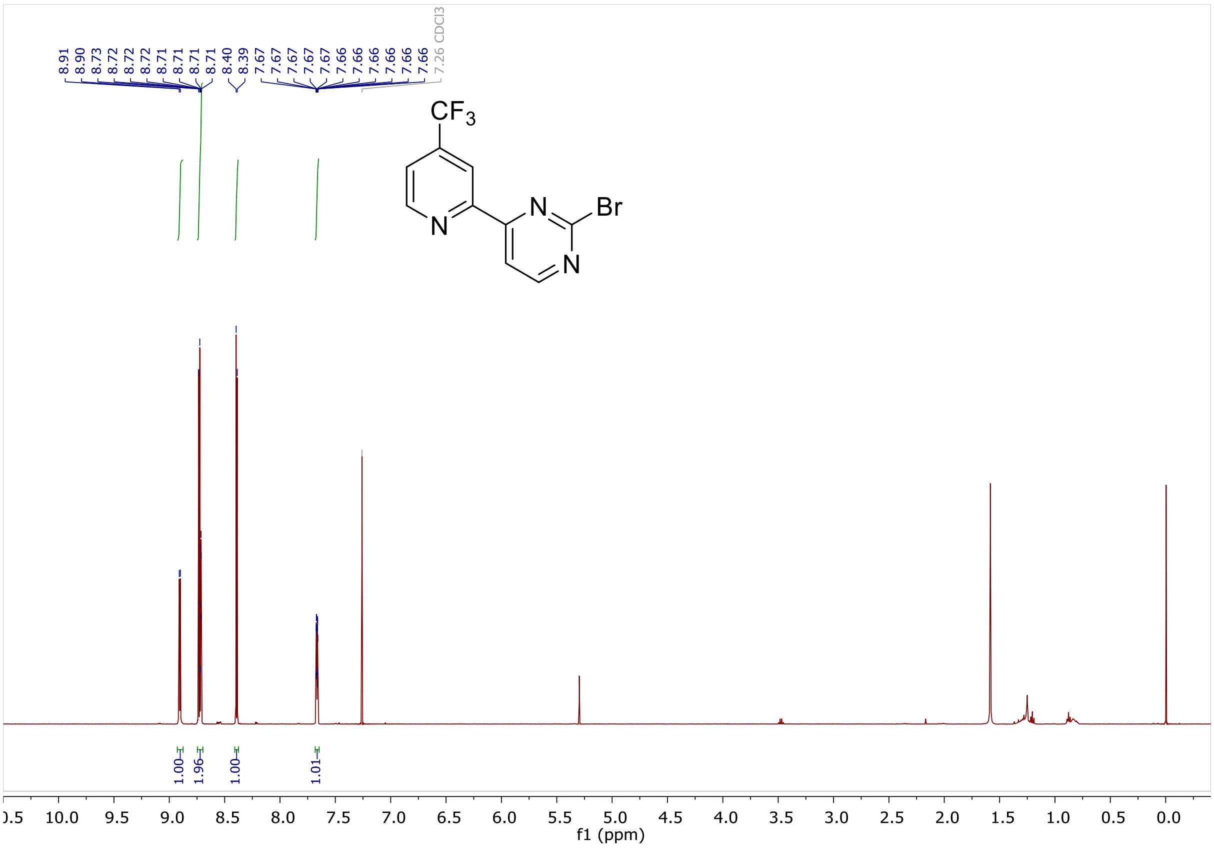

^{13}C NMR (101 MHz, CDCl$_3$): 26

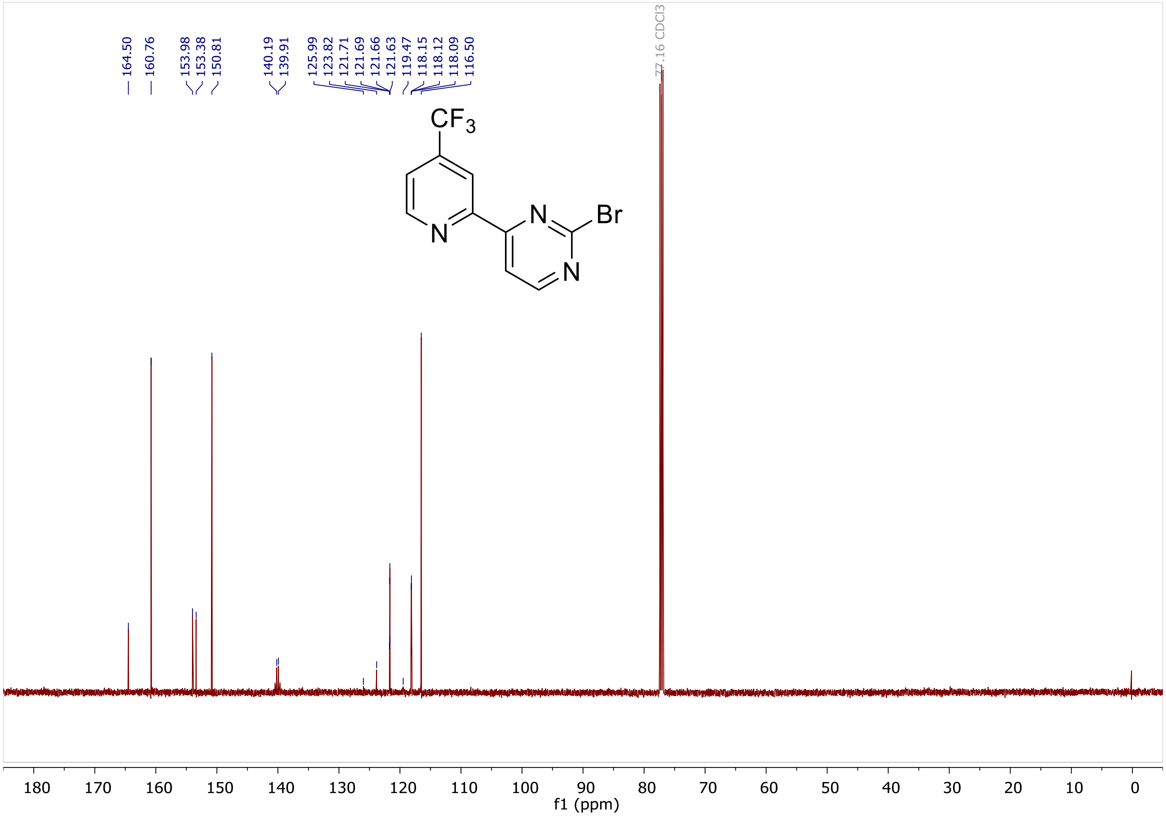

^{1}H NMR (400 MHz, $CDCl_3$): 27

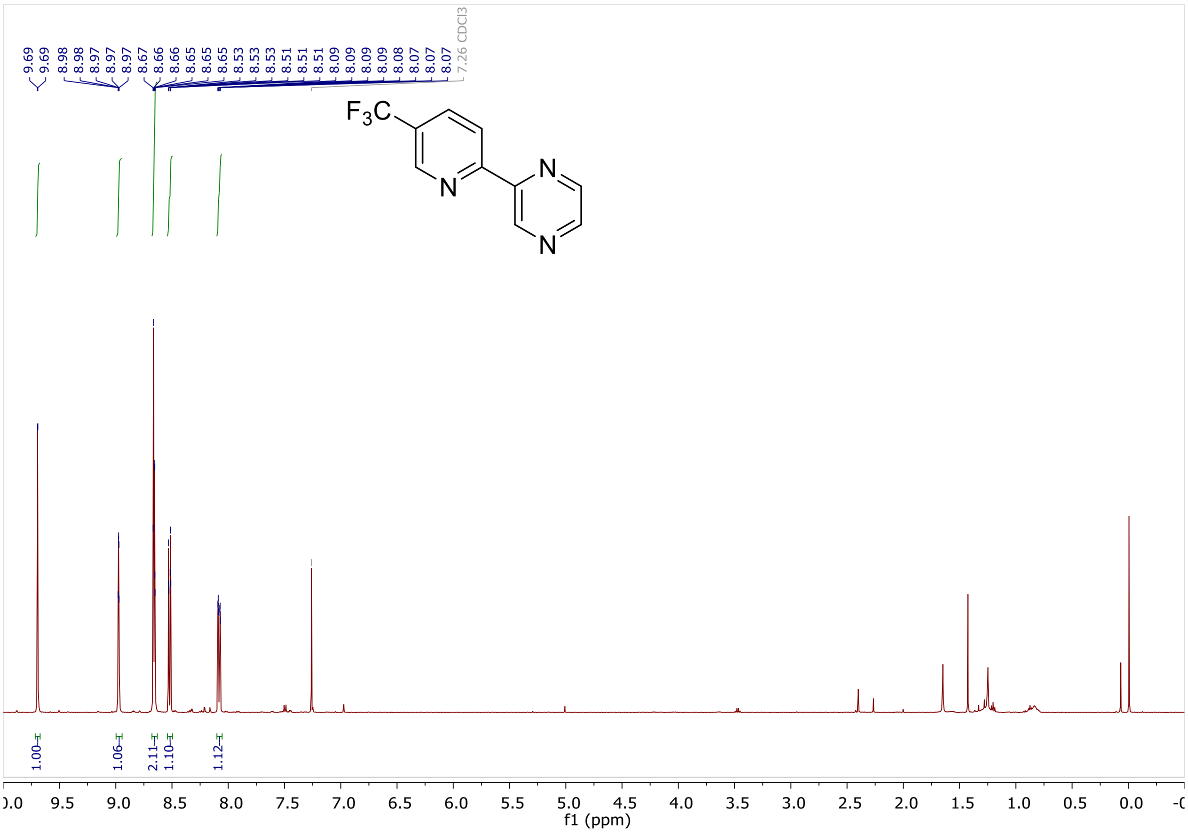

^{13}C NMR (101 MHz, $CDCl_3$): 27

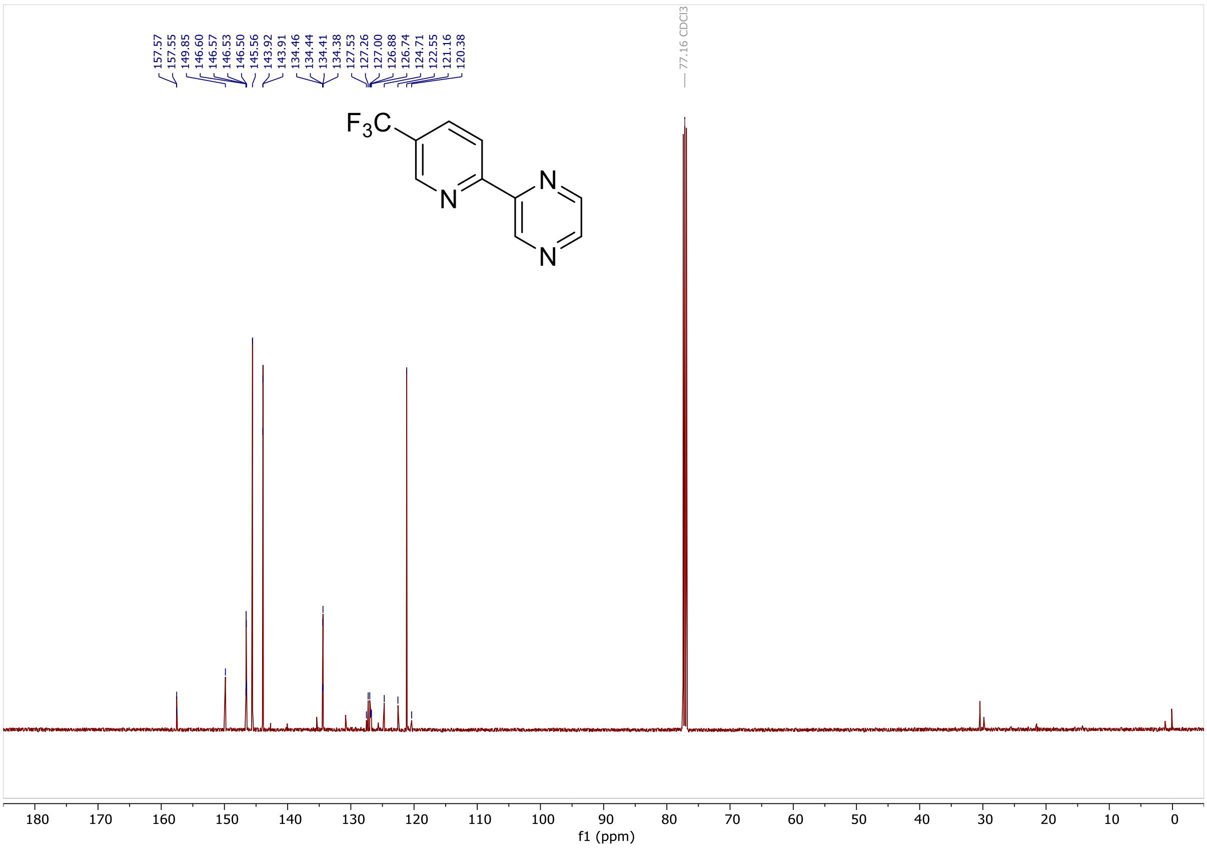

^{1}H NMR (400 MHz, $CDCl_3$): 28

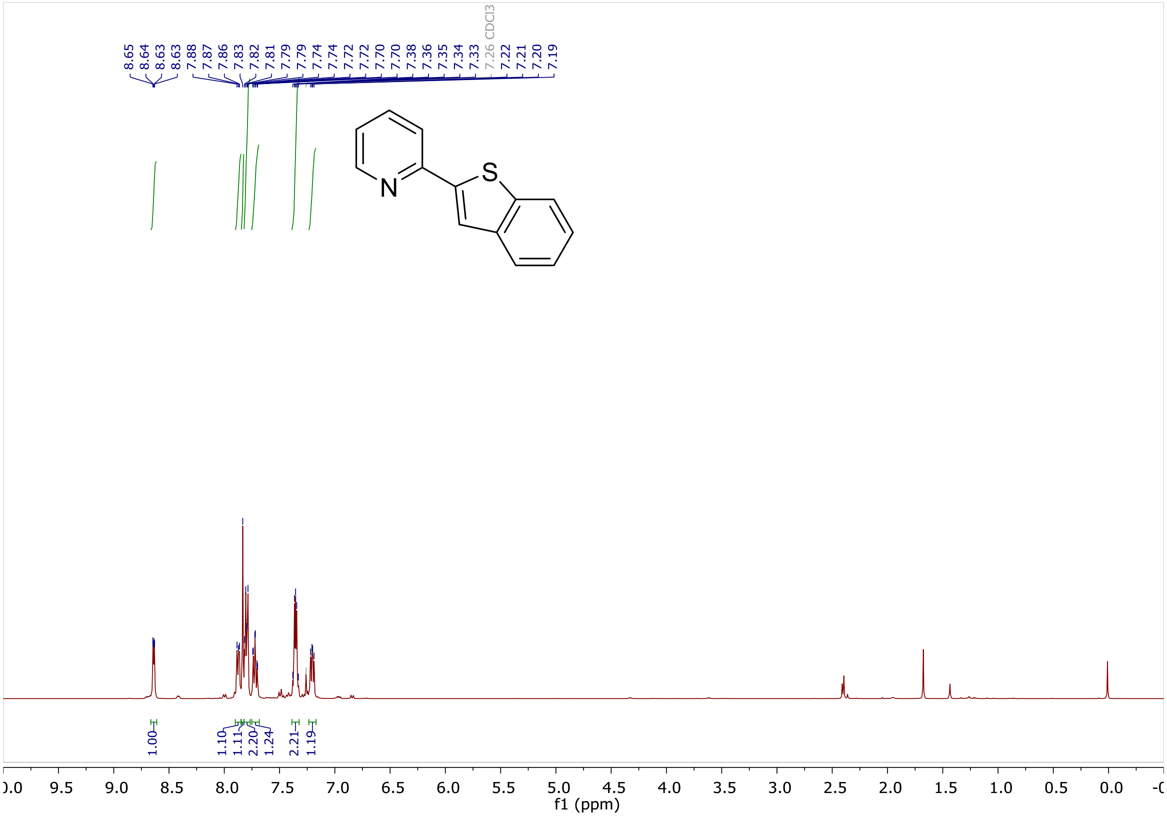

^{13}C NMR (101 MHz, $CDCl_3$): 28

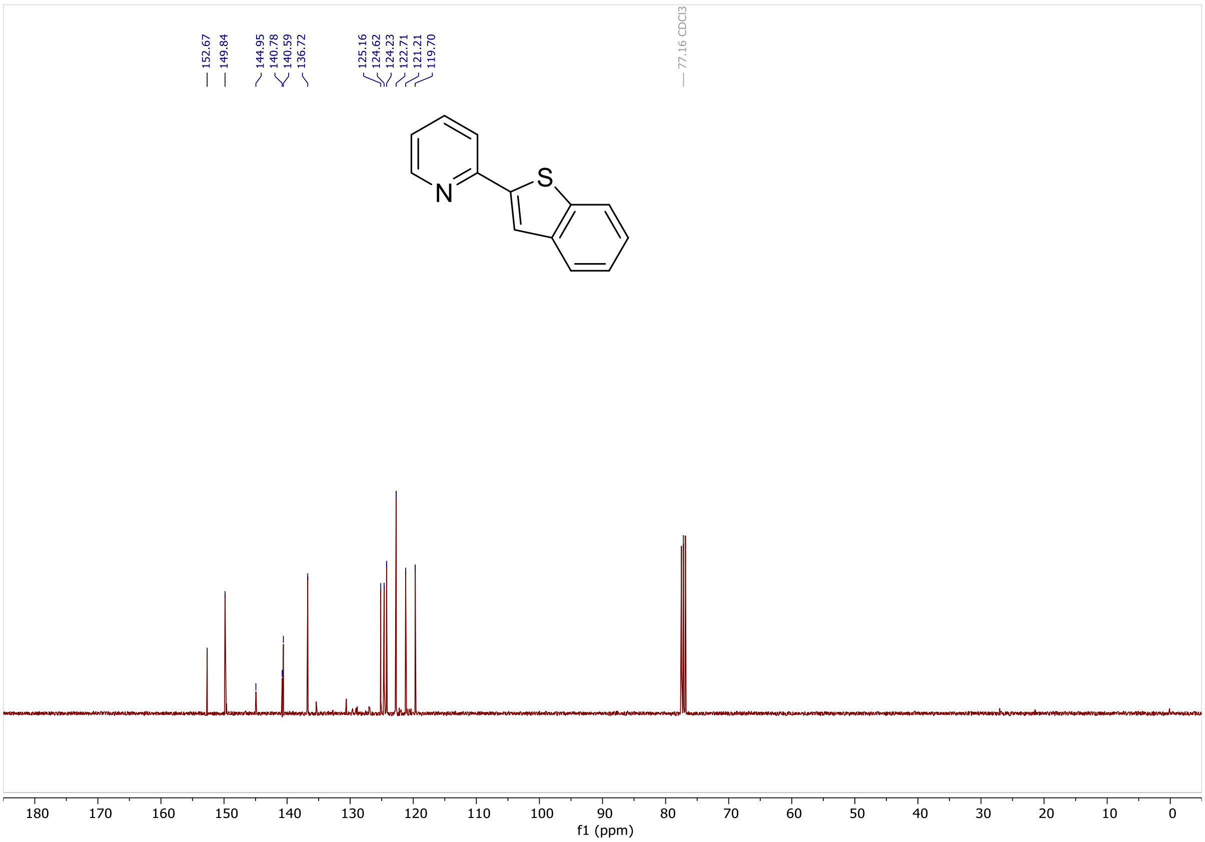

^{1}H NMR (400 MHz, $CDCl_3$): 29

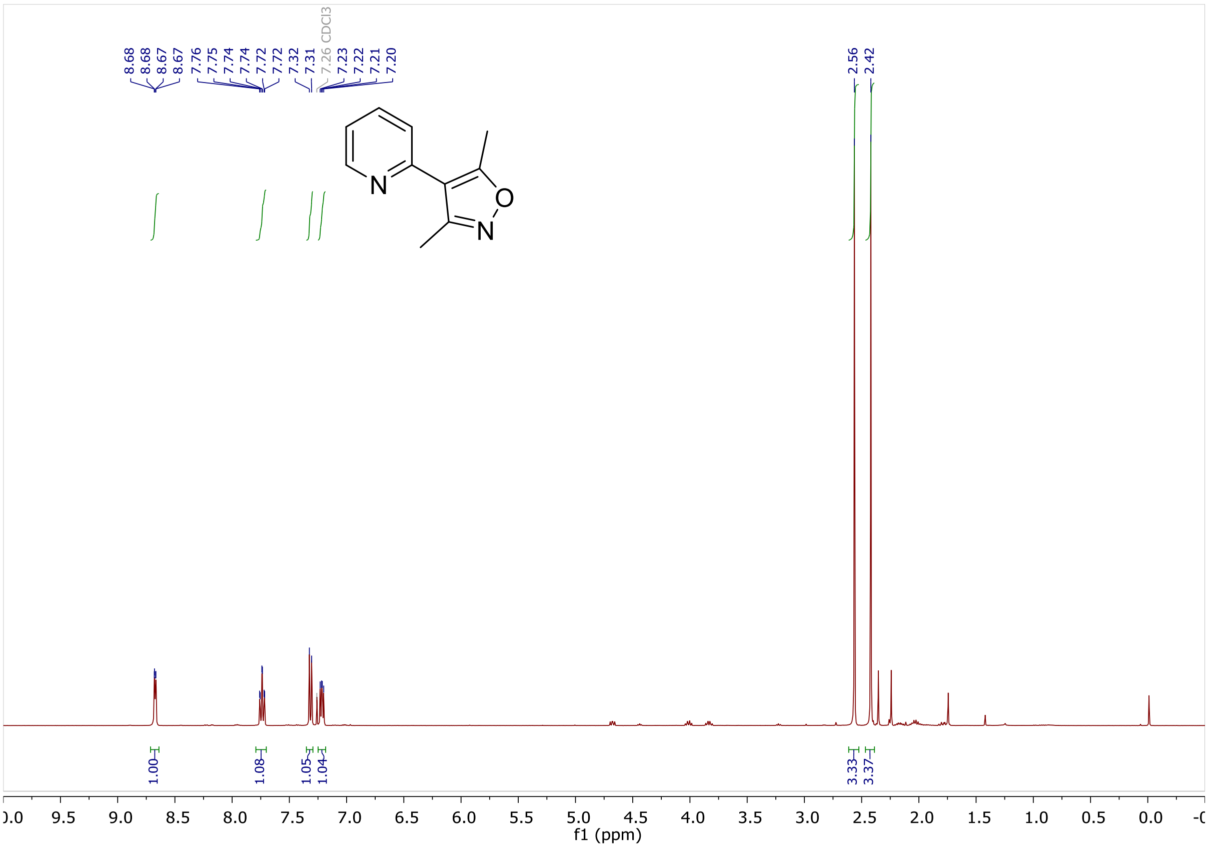

^{13}C NMR (101 MHz, $CDCl_3$): 29

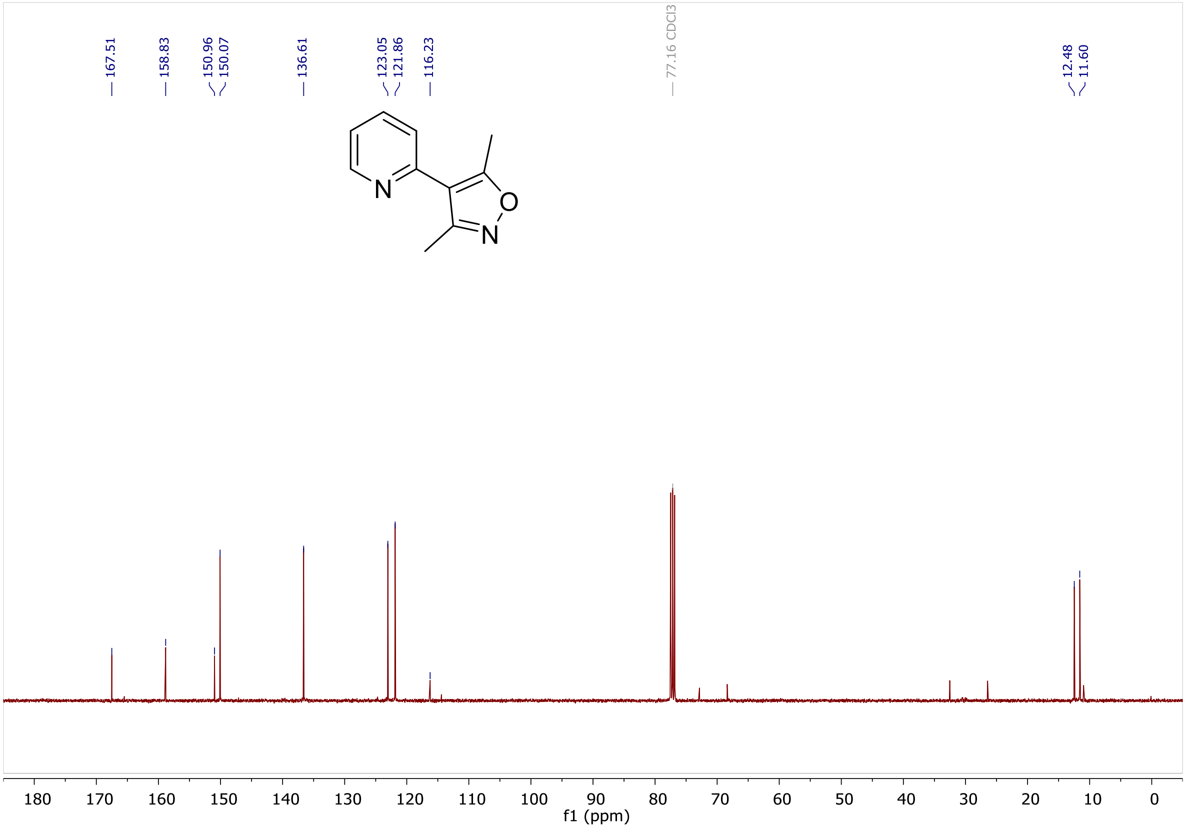

^{1}H NMR (400 MHz, $CDCl_3$): 30

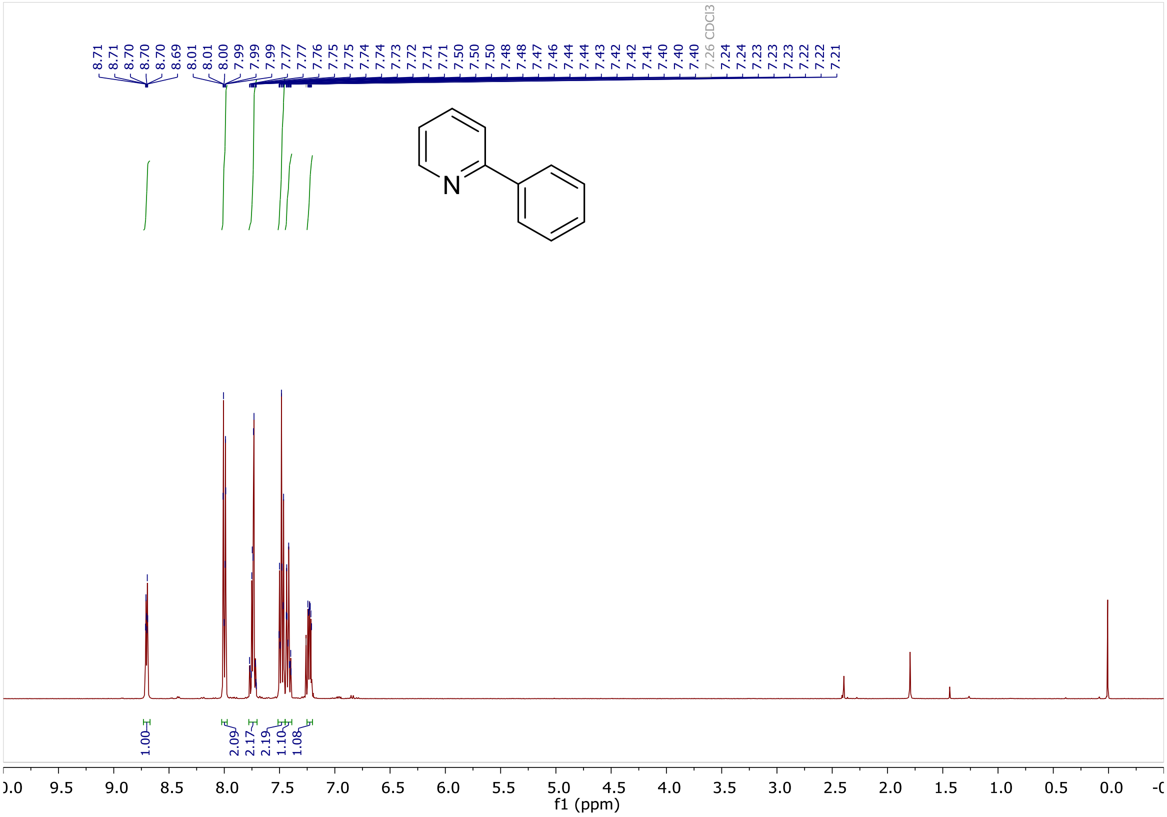

^{13}C NMR (101 MHz, $CDCl_3$): 30

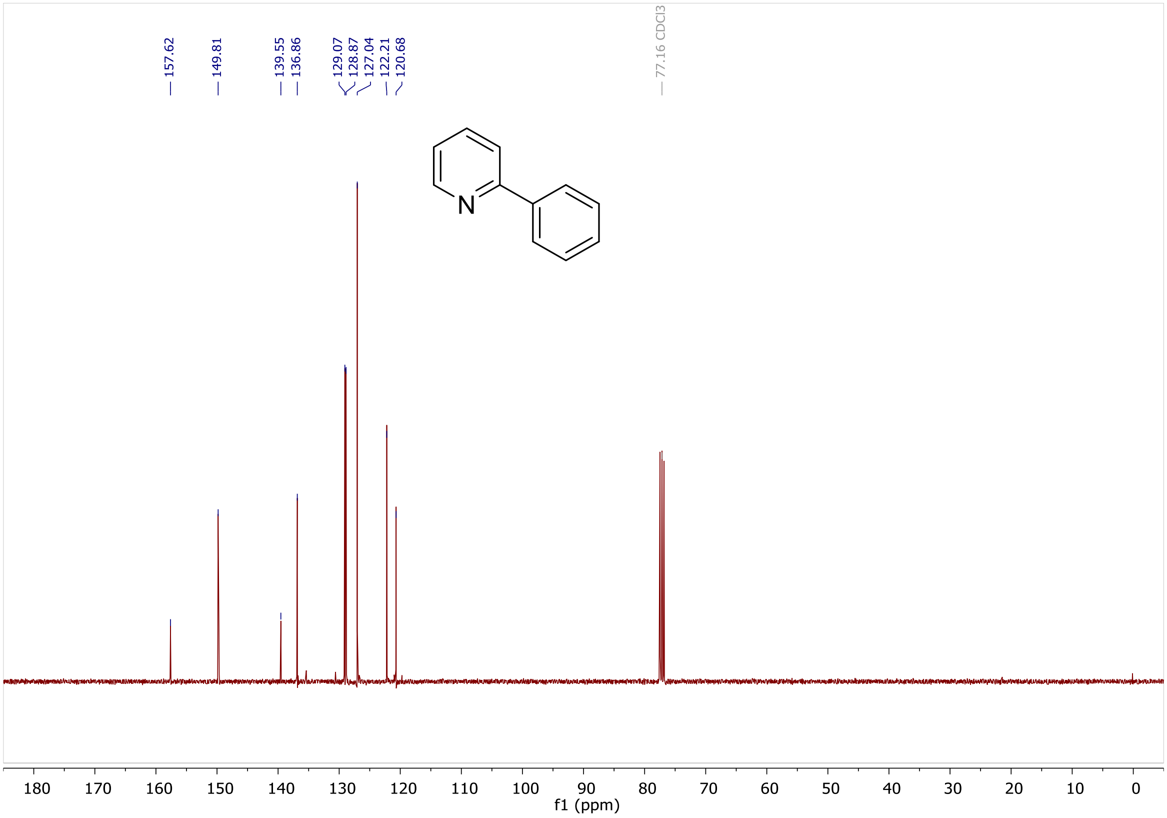

^{1}H NMR (400 MHz, $CDCl_3$): 36

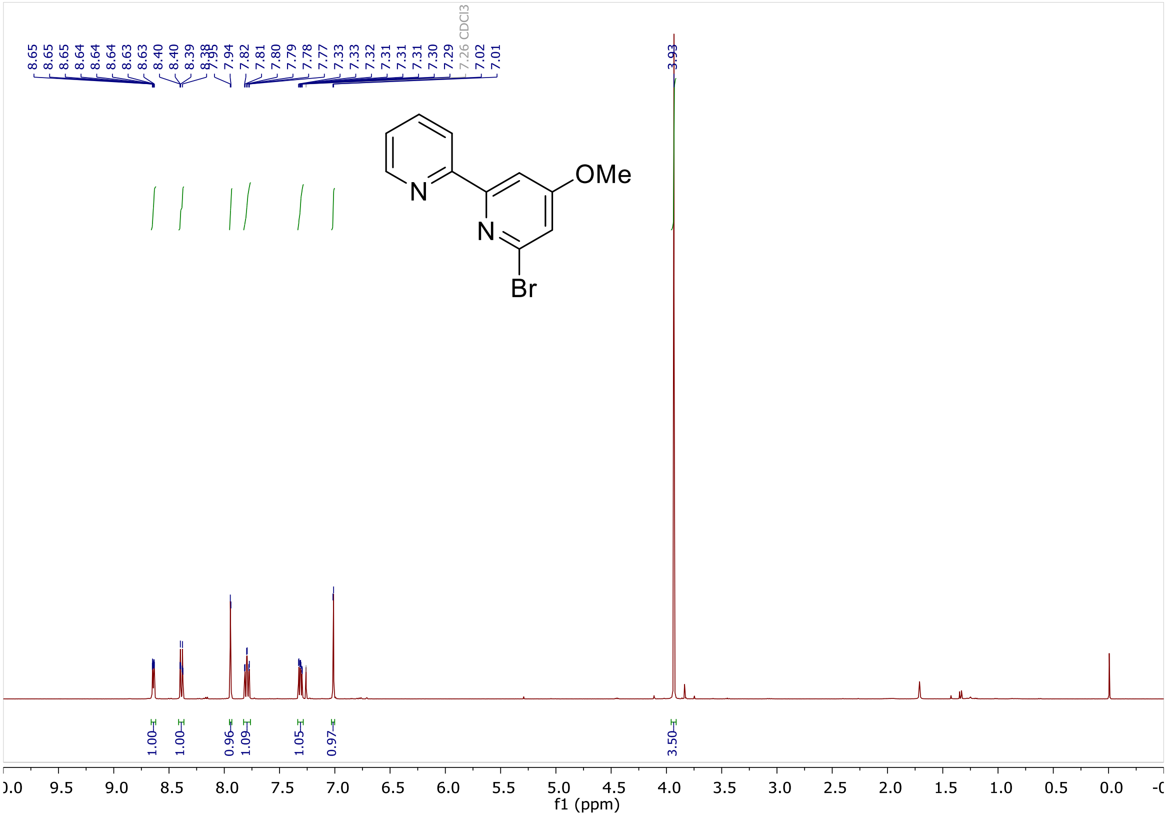

^{13}C NMR (101 MHz, $CDCl_3$): 36

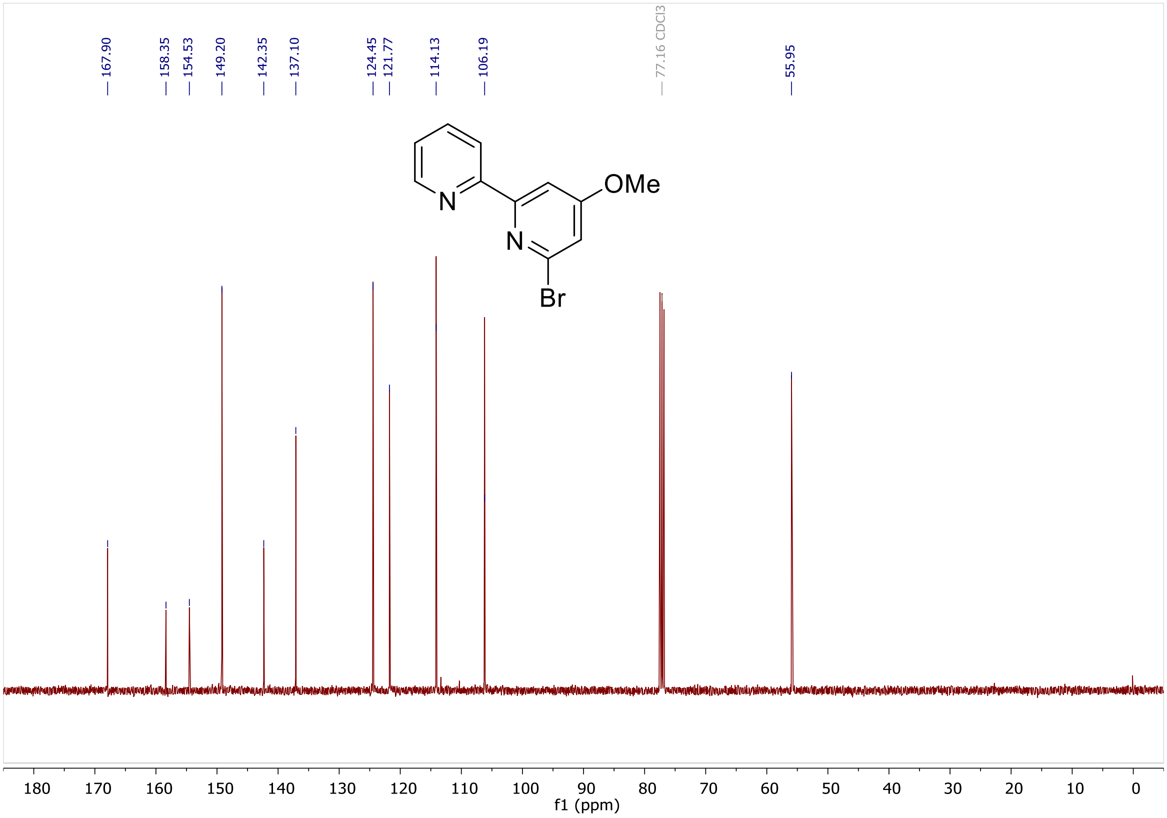

^{1}H NMR (400 MHz, $CDCl_3$): 37

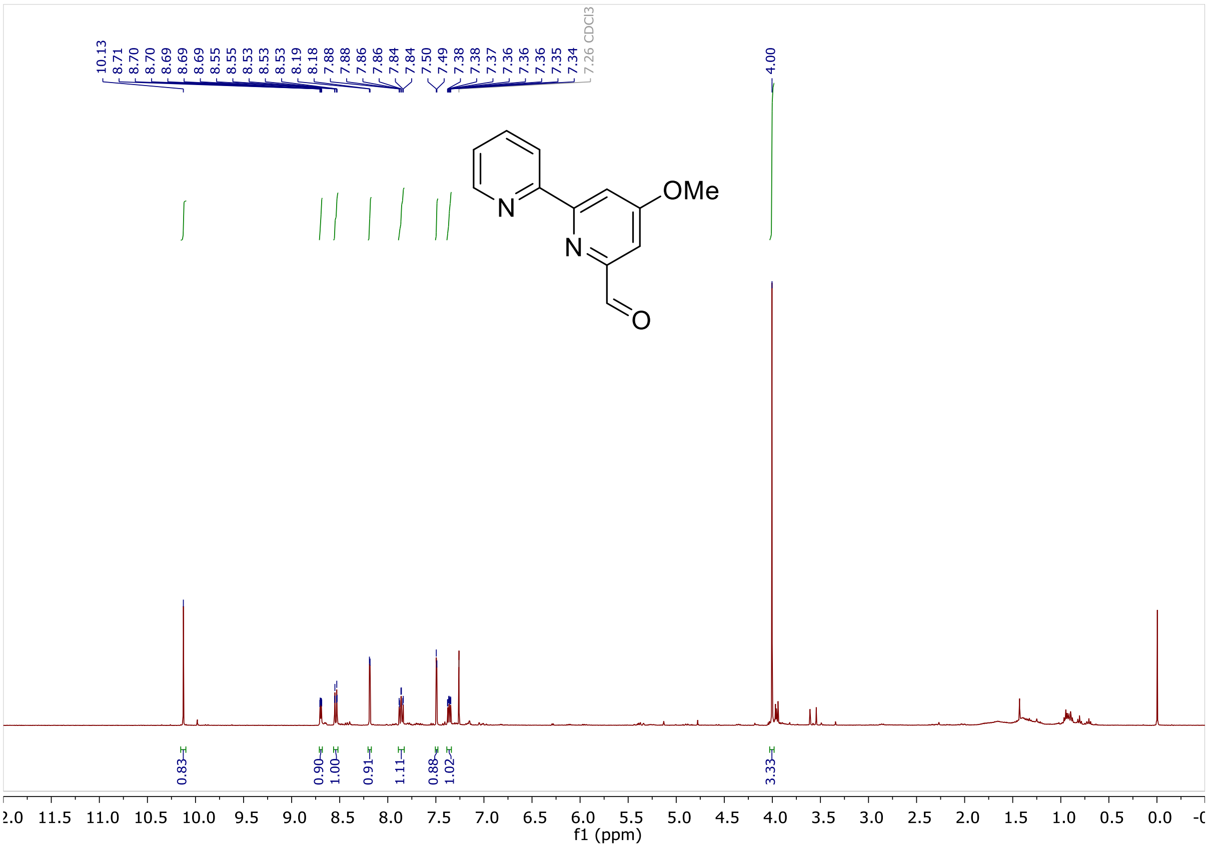

^{13}C NMR (101 MHz, $CDCl_3$): 37

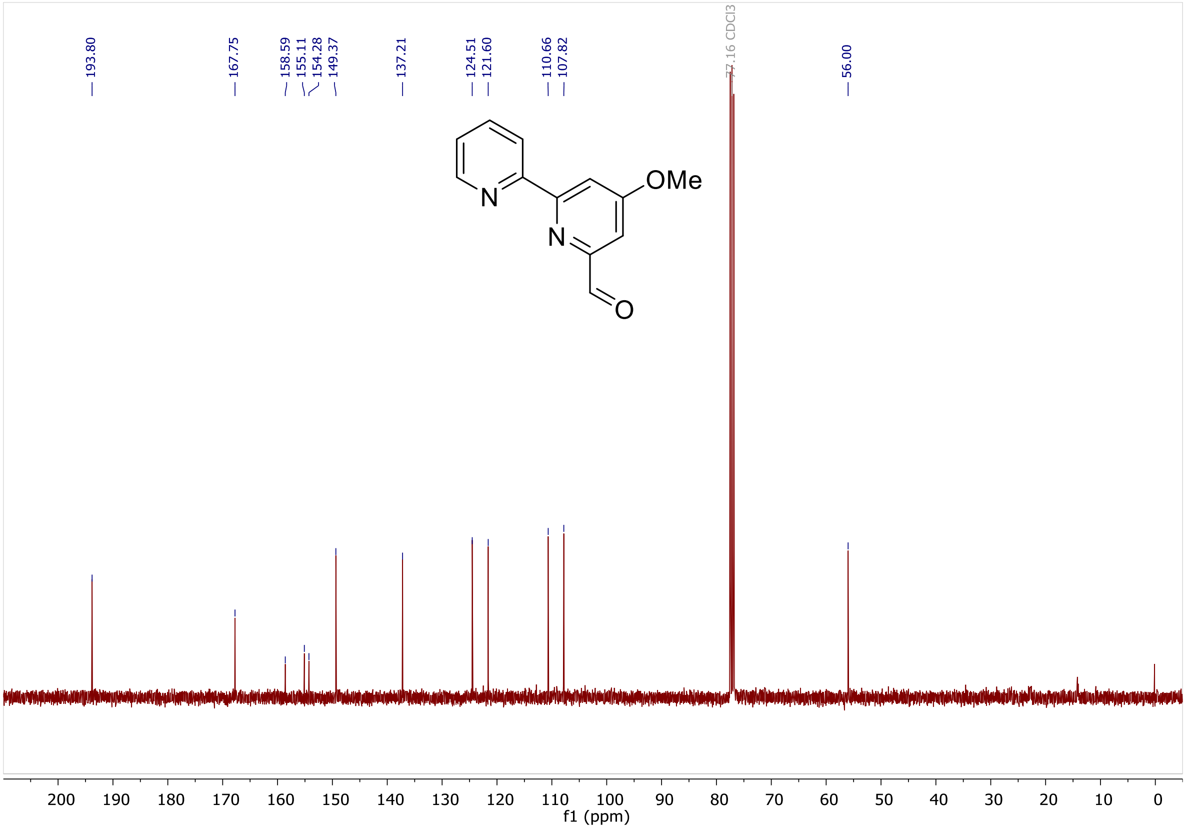

^{1}H NMR (400 MHz, DMSO-*d*6): 38

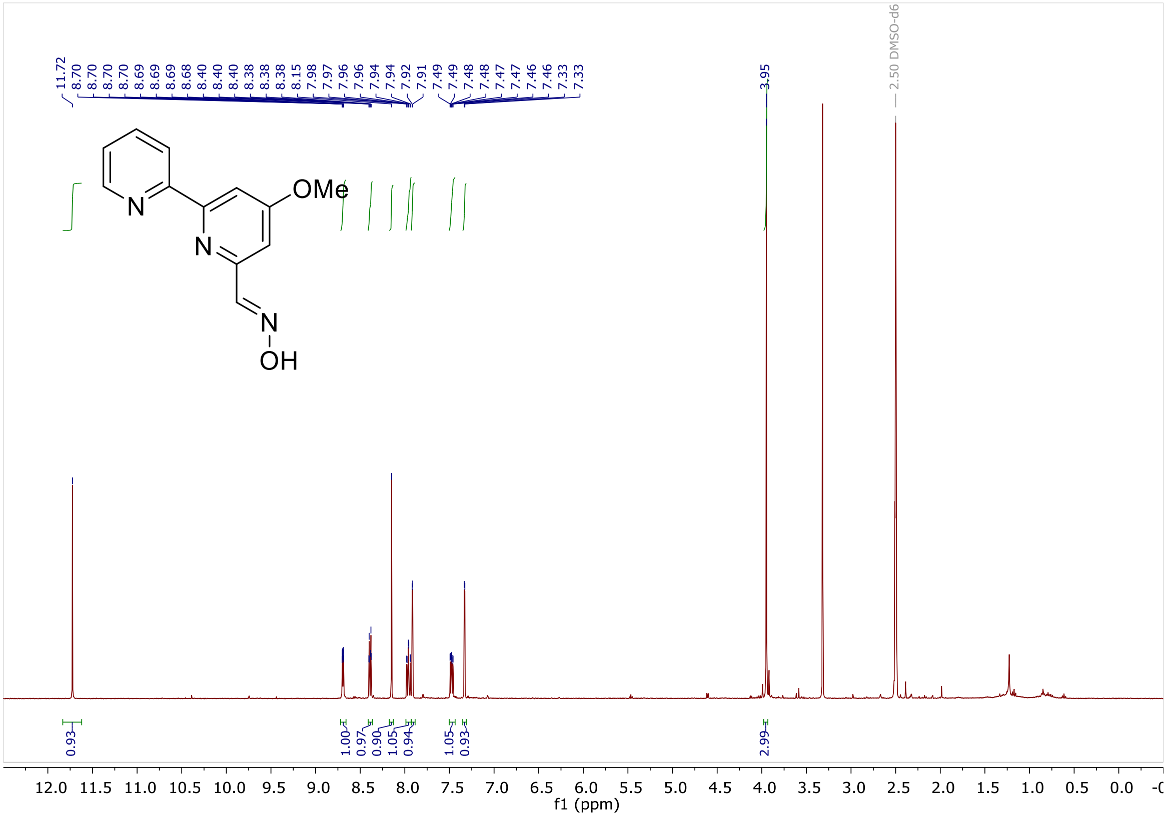

^{13}C NMR (101 MHz, DMSO-*d*6): 38

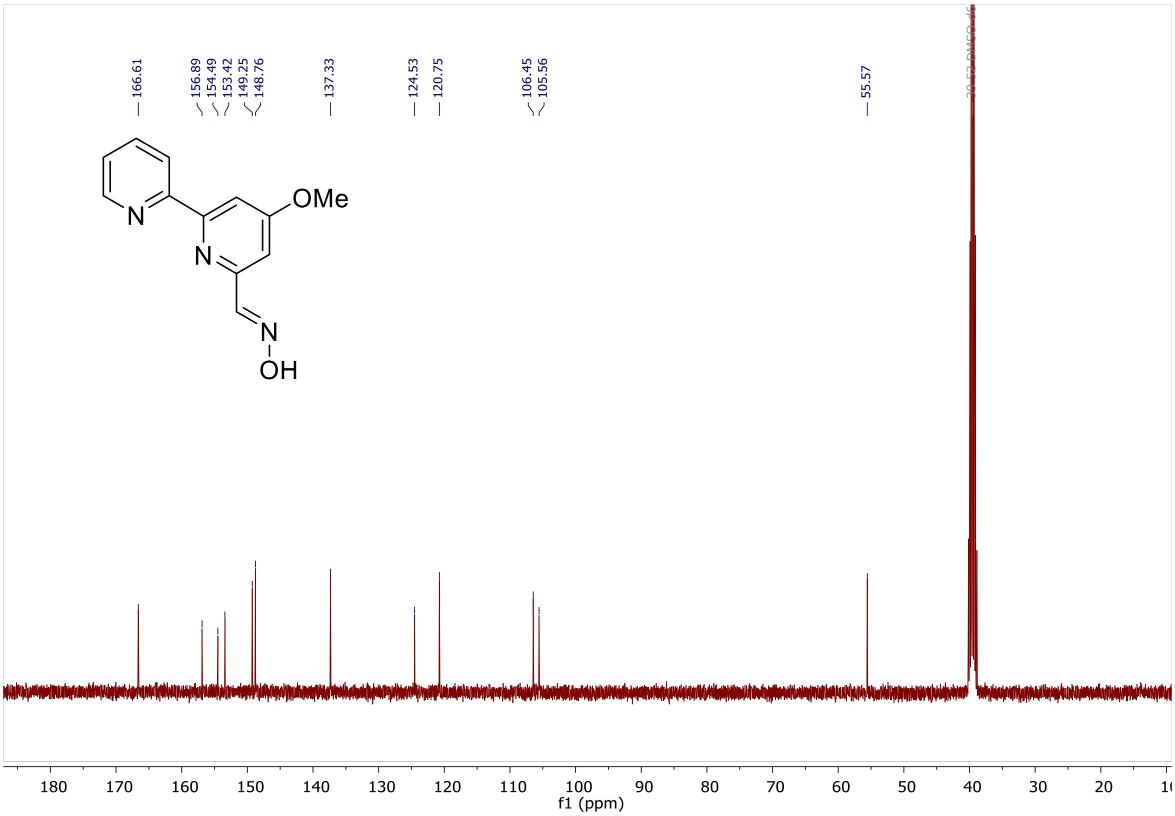

7. References

(1) Petersen, T. B.; Khan, R.; Olofsson, B. Metal-Free Synthesis of Aryl Esters from Carboxylic Acids and Diaryliodonium Salts. *Org. Lett.* **2011**, *13*, 3462–3465. https://doi.org/10.1021/ol2012082.

(2) Duong, V. K.; Horan, A. M.; McGarrigle, E. M. Synthesis of Pyridylsulfonium Salts and Their Application in the Formation of Functionalized Bipyridines. *Org. Lett.* **2020**, *22*, 8451–8457. https://doi.org/10.1021/acs.orglett.0c03048.

(3) Rohbogner, C. J.; Wagner, A. J.; Clososki, G. C.; Knochel, P. Magnesiation of weakly activated arenes using $Tmp_2MgCl.2LiCl$: Synthesis of *tert*-butyl ethyl phthalate. *Org. Synth.* **2009**, *86*, 374. https://doi.org/10.15227/orgsyn.086.0374.

(4) Watson, S. C.; Eastham, J. F. Colored Indicators for Simple Direct Titration of Magnesium and Lithium Reagents. *J. Organomet. Chem.* **1967**, *9*, 165–168.

(5) Arisawa, M.; Tazawa, T.; Tanii, S.; Horiuchi, K.; Yamaguchi, M. Rhodium-Catalyzed Synthesis of Unsymmetric Di(Heteroaryl) Sulfides Using Heteroaryl Ethers and S-Heteroaryl Thioesters via Heteroarylthio Exchange. *J. Org. Chem.* **2017**, *82*, 804–810. https://doi.org/10.1021/acs.joc.6b02585.

(6) Markovic, T.; Rocke, B. N.; Blakemore, D. C.; Mascitti, V.; Willis, M. C. Pyridine Sulfinates as General Nucleophilic Coupling Partners in Palladium-Catalyzed Cross-Coupling Reactions with Aryl Halides. *Chem. Sci.* **2017**, *8*, 4437–4442. https://doi.org/10.1039/c7sc00675f.

(7) Gao, G.; Xia, W.; Jain, P.; Yu, J.-Q. Pd(II)-Catalyzed C3-Selective Arylation of Pyridine with (Hetero)Arenes. *Org. Lett.* **2016**, *18*, 744–747. https://doi.org/10.1021/acs.orglett.5b03712.

(8) Bergmann, A. M.; Oldham, A. M.; You, W.; Brown, M. K. Copper-Catalyzed Cross-Coupling of Aryl-, Primary Alkyl-, and Secondary Alkylboranes with Heteroaryl Bromides. *Chem. Commun.* **2018**, *54*, 5381–5384. https://doi.org/10.1039/c8cc03145b.

(9) Hickey, D. P.; Sandford, C.; Rhodes, Z.; Gensch, T.; Fries, L. R.; Sigman, M. S.; Minteer, S. D. Investigating the Role of Ligand Electronics on Stabilizing Electrocatalytically Relevant Low-Valent Co(I) Intermediates. *J. Am. Chem. Soc.* **2019**, *141*, 1382–1392. https://doi.org/10.1021/jacs.8b12634.

(10) Zhou, M.; Tsien, J.; Qin, T. Sulfur(IV)-Mediated Unsymmetrical Heterocycle Cross-Couplings. *Angew. Chem. Int. Ed.* **2020**, *59*, 7372–7376. https://doi.org/10.1002/anie.201915425.

(11) Kim, S. H.; Rieke, R. D. 2-Pyridyl and 3-Pyridylzinc Bromides: Direct Preparation and Coupling Reaction. *Tetrahedron* **2010**, *66*, 3135–3146. https://doi.org/10.1016/j.tet.2010.02.061.

(12) Simkovsky, N. M.; Ermann, M.; Roberts, S. M.; Parry, D. M.; Baxter, A. D. Some Regioselective Cross-Coupling Reactions of Halopyridines and Halopyrimidines. *J. Chem. Soc. Perkin 1* **2002**, *16*, 1847–1849. https://doi.org/10.1039/b205027g.

(13) Chen, X.; Zhou, L.; Li, Y.; Xie, T.; Zhou, S. Synthesis of Heteroaryl Compounds through Cross-Coupling Reaction of Aryl Bromides or Benzyl Halides with Thienyl and Pyridyl Aluminum Reagents. *J. Org. Chem.* **2014**, *79*, 230–239. https://doi.org/10.1021/jo4024123.

(14) Truong, T.; Mesgar, M.; Le, K. K. A.; Daugulis, O. General Method for Functionalized Polyaryl Synthesis via Aryne Intermediates. *J. Am. Chem. Soc.* **2014**, *136*, 8568–8576. https://doi.org/10.1021/ja504886x.

(15) Molander, G. A.; Canturk, B.; Kennedy, L. E. Scope of the Suzuki-Miyaura Cross-Coupling Reactions of Potassium Heteroaryltrifluoroborates. *J. Org. Chem.* **2009**, *74*, 973–980. https://doi.org/10.1021/jo802590b.

(16) Chen, W. C.; Hsu, Y. C.; Shih, W. C.; Lee, C. Y.; Chuang, W. H.; Tsai, Y. F.; Chen, P. P. Y.; Ong, T. G. Metal-Free Arylation of Benzene and Pyridine Promoted by Amino-Linked Nitrogen Heterocyclic Carbenes. *Chem. Commun.* **2012**, *48*, 6702–6704. https://doi.org/10.1039/c2cc32519e.

(17) Trecourt, F.; Gervais, B.; Mongin, O.; Gal, C. Le; Mongin, F.; Queguiner, G. First Syntheses of Caerulomycin E and Collismycins A and C . A New Synthesis of Caerulomycin A. *J. Org. Chem.* **1998**, *63*, 2892–2897. https://doi.org/10.1021/jo972022i.
